# Supplementary material for: Usual care and a self-management support programme versus usual care and a relaxation programme for people living with chronic headache disorders: a randomised controlled trial protocol (CHESS)
Source: BMJ Open. 2020 Apr 12;10(4):e033520. doi: 10.1136/bmjopen-2019-033520 (PMC7200026; doi:10.1136/bmjopen-2019-033520)
Supplement: Author's manuscript [file bmjopen-2019-033520.draft_revisions.pdf]

BMJ Open is committed to open peer review. As part of this commitment we make the peer review history of every article we publish publicly available.

When an article is published we post the peer reviewers' comments and the authors' responses online. We also post the versions of the paper that were used during peer review. These are the versions that the peer review comments apply to.

The versions of the paper that follow are the versions that were submitted during the peer review process. They are not the versions of record or the final published versions. They should not be cited or distributed as the published version of this manuscript.

BMJ Open is an open access journal and the full, final, typeset and author-corrected version of record of the manuscript is available on our site with no access controls, subscription charges or pay-per-view fees (http://bmjopen.bmj.com).

If you have any questions on BMJ Open's open peer review process please email info.bmjopen@bmj.com

BMJ Open

Usual care and a self-management support programme vs usual care and a relaxation programme for people living chronic headache disorders: a randomised controlled trial protocol (CHESS)

Journal:	*BMJ Open*
Manuscript ID	bmjopen-2019-033520
Article Type:	Protocol
Date Submitted by the Author:	08-Aug-2019

Complete List of Authors:	Patel, Shilpa; University of Warwick, Warwick Medical School, Clinical Trials Unit<br>Carnes, Dawn; Queen Mary University of London, Centre for Primary Care and Public Health<br>Eldridge, Sandra; Queen Mary University of London, Centre for Primary Care and Public Health<br>Ellard, David; University of Warwick, Warwick Medical School, Clinical Trials Unit<br>Griffiths, Frances; University of Warwick, Warwick Medical School, Division of Health Sciences<br>Haywood, Kirstie; University of Warwick, Warwick Medical School, Division of Health Sciences<br>Hee, Siew Wan; University of Warwick, Warwick Medical School<br>Mistry, Dipesh; University of Warwick, Warwick Medical School, Clinical Trials Unit<br>Mistry, Hema; University of Warwick, Warwick Medical School, Warwick Evidence<br>Nichols, Vivien; University of Warwick, Warwick Medical School, Clinical Trials Unit<br>Petrou, Stavros; University of Warwick, Warwick Medical School<br>Pincus, Tamar; Royal Holloway University of London, Psychology<br>Potter, Rachel; University of Warwick, Warwick Medical School, Clinical Trials Unit<br>Sandhu, Harbinder; University of Warwick Warwick Medical School, Clinical Trials Unit<br>Taylor, Stephanie; Queen Mary University of London, Centre for Primary Care and Public Health<br>Underwood, Martin; University of Warwick, Warwick Medical School, Clinical Trials Unit<br>White, Kimberley; University of Warwick, Warwick Medical School, Clinical Trials Unit<br>Matharu, Manjit; University College London Hospitals NHS Foundation Trust National Hospital for Neurology and Neurosurgery

Keywords:	chronic headache, tension type headache, Migraine < NEUROLOGY, self-management, PRIMARY CARE, study protocol

For peer review only - http://bmjopen.bmj.com/site/about/guidelines.xhtml

TITLE: Usual care and a self-management support programme vs usual care and a relaxation programme for people living chronic headache disorders: a randomised controlled trial protocol (CHESS)

AUTHORS:

Shilpa Patel, Warwick Clinical Trials Unit, Warwick Medical School, University of Warwick, Coventry. CV4 7AL, UK Shilpa.Patel@warwick.ac.uk

Dawn Carnes, Centre for Primary Care and Public Health, Blizard Institute Barts and The London School of Medicine and Dentistry, Queen Mary University of London, London, UK d.carnes@qmul.ac.uk

Sandra Eldridge, Centre for Primary Care and Public Health, Blizard Institute Barts and The London School of Medicine and Dentistry, Queen Mary University of London, London, UK s.eldridge@qmul.ac.uk

David Ellard, Warwick Clinical Trials Unit, Warwick Medical School, University of Warwick, Coventry, CV4 7AL, UK D.R.Ellard@warwick.ac.uk

Frances Griffiths, Health Sciences, Warwick Medical School, University of Warwick, CV4 7AL, UK f.e.griffiths@warwick.ac.uk

Kirstie Haywood, Division of Health Sciences, Warwick Medical School, University of Warwick, Coventry, CV4 7A, UK K.L.Haywood@warwick.ac.uk

Siew Wan Hee, Statistics and Epidemiology Unit, Warwick Medical School, University of Warwick, CV4 7AL, UK s.w.hee@warwick.ac.uk

Dipesh Mistry, Warwick Clinical Trials Unit, Warwick Medical School, University of Warwick, Coventry, CV4 7AL, UK D.Mistry@warwick.ac.uk

Hema Mistry, Population Evidence and Technologies, Warwick Medical School, University of Warwick, Coventry, CV4 7AL, UK Hema.Mistry@warwick.ac.uk

Vivien Nichols, Warwick Clinical Trials Unit, Warwick Medical School, University of Warwick, Coventry, CV4 7AL, UK V.P.Nichols@warwick.ac.uk

Stavros Petrou, Warwick Clinical Trials Unit, Warwick Medical School, University of Warwick, Coventry, CV4 7AL, UK S.Petrou@warwick.ac.uk

Tamar Pincus, Royal Holloway, University of London, Department of Psychology, Surrey, TW20 0EX, UK T.Pincus@rhul.ac.uk

Rachel Potter, Warwick Clinical Trials Unit, Warwick Medical School, University of Warwick, Coventry, CV4 7AL, UK r.potter@warwick.ac.uk

Harbinder Sandhu, Warwick Clinical Trials Unit, Warwick Medical School, University of Warwick, Coventry, CV4 7AL, UK Harbinder.K.Sandhu@warwick.ac.uk

For peer review only - http://bmjopen.bmj.com/site/about/guidelines.xhtml

Stephanie JC Taylor, Centre for Primary Care and Public Health, Blizard Institute Barts and The London School of Medicine and Dentistry, Queen Mary University of London, London, UK s.j.c.taylor@qmul.ac.uk

Martin Underwood, Warwick Clinical Trials Unit, Warwick Medical School, University of Warwick, Coventry, CV4 7AL, UK M.Underwood@warwick.ac.uk

Kimberley White, Warwick Clinical Trials Unit, Warwick Medical School, University of Warwick, Coventry, CV4 7AL, UK Kimberley.White@warwick.ac.uk

Manjit Matharu, Headache Group, UCL Institute of Neurology and The National Hospital for Neurology and Neurosurgery, Queen Square, London, WC1N 3BG, UK manjit.matharu@nhs.net

On behalf of the CHESS team.

Corresponding author:
Shilpa Patel, Warwick Clinical Trials Unit, Warwick Medical School, University of Warwick, Coventry. CV4 7AL Shilpa.Patel@warwick.ac.uk
Tel: 44 (0) 2476 150 405

For peer review only - http://bmjopen.bmj.com/site/about/guidelines.xhtml

ABSTRACT

Introduction: Chronic headaches are poorly diagnosed and managed, and can be exacerbated by medication overuse. There is insufficient evidence on the non-pharmacological approaches to helping people living with chronic headaches.

Methods and analysis: CHESS (Chronic Headache Education and Self-management Study) is a pragmatic randomised controlled trial to test the effectiveness and cost effectiveness of a self-management education support programme on top of usual care for patients with chronic headaches against a control of usual care and relaxation. The intervention is a two day group course based on education, personal reflection and a cognitive behavioural approach, plus a nurse-led one-to-one consultation and follow up over 8 weeks. We aim to recruit 689 participants (356 to the intervention arm and 333 to the control) from primary care and self-referral in London and the Midlands. The trial is powered to show a difference of 2.0 points on the Headache Impact Test (HIT6); a patient reported outcome measure at 12 months post randomisation. Secondary outcomes include health related quality of life, self-efficacy, social activation and engagement, anxiety and depression and health care utilisation. Outcomes are being measured at four, eight and 12 months. Cost-effectiveness will be expressed in terms of incremental cost per quality-adjusted life year gained.

Ethics and dissemination: This trial will provide data on effectiveness and cost-effectiveness of a self-management support programme for chronic headaches. The results will inform commissioning services and clinical practice. North West - Greater Manchester East Research Ethics Committee have approved the trial. The current protocol version is 3.6 date 7th March 2019.

Trial registration number: ISRCTN79708100. Registered 16th December 2015, http://www.isrctn.com/ISRCTN79708100

Keywords: Chronic headache, tension type headache, migraine, self-management, primary care, study protocol

For peer review only - http://bmjopen.bmj.com/site/about/guidelines.xhtml

ARTICLE SUMMARY

Strengths and limitations of the study

- This trial presents a high quality randomised controlled trial designed to determine the effectiveness and cost-effectiveness of a group self-management support programme for adults with chronic headaches.
- The intervention underpinning the trial is a complex intervention which has been designed using the best available evidence and theory. The full details of the intervention development and design have been peer reviewed and published.
- A separate mixed-methods process evaluation is running alongside the trial and will explore fidelity of the intervention and implementation. A detailed process evaluation protocol has been peer reviewed and published.
- The success of the trial will be dependent on the ability to deliver this complex group intervention, ensure fidelity and subsequently obtain follow-up data in this population.

For peer review only

INTRODUCTION

Headache disorders are a major cause of pain and disability.[1] The definition of a chronic headache is that the person has headache for 15 or more days per month for at least three months (https://www.ichd-3.org/). Chronic headache disorders mainly affect the young adult population, many of whom have both work and family commitments.[2] The most common chronic headache disorders are chronic tension type headache, chronic migraine, and medication overuse headaches (MOH).[1] Tension type headache and migraine are primary headaches. Medication overuse is a secondary headache that can develop in people with frequent acute headaches who take analgesics, or specific anti-migraine compounds (e.g. triptans) for ≥10 days per month.

Around 2-4% of the global population experience chronic headaches.[3] Between a quarter and half of those affected also have MOH, which has a prevalence of 1%.[4-6] Many people with chronic headache have undiagnosed chronic migraine.[7] More appropriate use of pharmacological treatment has the potential to improve outcomes for people living with chronic headache disorders. This might mean introducing migraine prophylactic drugs or using fewer analgesics/triptans. Depression, anxiety, poor sleep, stress and poor self-efficacy for managing headaches are prognostic markers for a poor outcome in chronic headache disorders.[8] Limited qualitative data indicate that chronic headache disorders can directly and indirectly drive behaviour: that people live with the 'spectre of headache' (an array of concerns people with chronic headaches have to take into account when forward planning) and that headaches lead to strained relationships.[9] For people living with chronic migraine or chronic tension type headache non-pharmacological self-management approaches may improve headache related symptoms, whilst not affecting headache frequency.[10] Group interventions using a cognitive behavioural approach, mindfulness and educational components may be more effective than alternative interventions.[10]

Supportive self-management approaches are well established in the management of several chronic painful disorders, but this is not the case for chronic headache disorders.[11-13] The National Institute for Health and Care Excellence (NICE) guidance in England, for example, only makes one positive recommendation for a non-pharmacological treatment for people living with chronic headaches; to consider a course of acupuncture for people with chronic migraine or chronic tension type headache.[14]

We describe here the protocol for a randomised controlled trial (RCT) to estimate the effectiveness and cost-effectiveness of a group self-management support programme for adults with chronic headaches arising from migraine or tension type headache, with or without medication overuse. The CHESS intervention consists of a group education and self-management support programme plus a tailored one-to-one headache consultation exploring medication optimisation, lifestyle factors and goals. This active arm of the trial is being compared to usual care plus a relaxation programme.

METHODS AND ANALYSIS

The methods section is structured in accordance with the SPIRIT 2013 recommendations.[15] Here we provide an overview of the trial plus a brief description of the proposed health economic evaluation, process evaluation and patient and public involvement.

The primary objective is to estimate the clinical and cost-effectiveness of a group education and self-management programme for people living with chronic headache recruited from primary care when compared to a GP care plus relaxation control group.

For peer review only - http://bmjopen.bmj.com/site/about/guidelines.xhtml

1. Participants, interventions and outcomes

Study setting

Primary care settings in two localities; London and the Midlands.

Eligibility criteria

The population of interest are adults living with chronic migraine or chronic tension type headache, with or without medication overuse. Part of the challenge in interpreting the findings of research in people living with chronic headaches is the often poor reporting of participants' phenotypic characteristics, meaning it is not possible to draw conclusions for specific chronic headache types.[9 16] For this trial, it is important that we are able to define our participants' headache type, or types, accurately and exclude those with non-eligible headache types. We, therefore developed and validated a logic model for use in a telephone interview by a nurse (non-headache expert) that would allow us to identify and classify people meeting our entry criteria, whilst also excluding people with secondary headaches, other than medication overuse and other causes of primary headaches.[17]

Table 1: Inclusion and exclusion criteria

Inclusion criteria	Exclusion criteria
1. Able and willing to comply with the study procedures and provide written informed consent<br>2. Aged ≥18 years<br>3. Living with chronic headache; defined as headache on 15 or more days per month for at least the preceding three months<br>4. The nurse telephone classification interview confirms headache type to be chronic migraine, or chronic tension type headache, with or without medication overuse headache<br>5. Fluent in written and spoken English.	1. Unable to attend the group sessions<br>2. No access to a telephone<br>3. Has an underlying serious psychological disorder with ongoing symptoms that preclude or significantly interfere with participation in the group intervention<br>4. Previous entry or randomisation in the present trial<br>5. Currently participating in another clinical trial of headache treatments or unregistered medicinal product, or less than 90 days have passed since completing participation in such a trial.

Pregnancy is not an exclusion criterion. However, any pregnant women randomised to the active intervention will be advised to speak to their General Practitioner (GP) with regards to medication and the CHESS intervention nurses will not discuss this with them during the consultation for safety reasons.

Our previous experience is that the challenges of running non-English language group interventions for chronic painful disorders are too great to do successfully within a randomised controlled trial.[18] Furthermore the main outcomes are not validated in those languages other than English which are relevant to a UK context. For these reasons we are excluding people who are not fluent in written and spoken English

For peer review only - http://bmjopen.bmj.com/site/about/guidelines.xhtml

Interventions

The intervention consists of a group education and self-management programme, an eight-week headache paper diary, a one-to-one nurse-led consultation and follow-up telephone calls, where necessary, for up to eight weeks. We have described its development in detail elsewhere.[19] We summarise it briefly here.

The programme is run over two days in a two week period for around 10 participants per group (target six to twelve participants), is facilitated by two intervention trained health care professionals, at least one of whom is a nurse. We originally planned to run sessions using a nurse, and a lay facilitator living with chronic headaches. However, in our feasibility study we found it difficult to recruit people living with chronic headaches to act as facilitators and of those that were recruited several found it difficult to commit to facilitation of courses because of the unpredictability of their condition. The intervention builds on a previously developed and tested educational and cognitive behavioural self-management intervention for people with chronic musculoskeletal pain.[20]

The aim of the course is to encourage and enable those with chronic headaches to recognise unhelpful thought patterns and behaviours that contribute to their headache burden and to do something about them. The course provides participants with an overall toolbox of strategies that could help in the management of their headaches. These strategies include psychological techniques to change perceptions and feelings about living with chronic headaches as well as more practical strategies around lifestyle factors and medication. The group intervention is delivered using a range of methods including: group discussions, ideas generation, sharing narratives and experiences, problem solving, role play and taster activity sessions. The programme includes a range of behaviour change techniques including; barrier identification, general encouragement, instruction from the group facilitators, provision of feedback, and allowing opportunities for social comparison in the group. We also include an educational video (available on disc and on-line) following feedback from our patient and public involvement (PPI) group on the importance of having something to show family and friends about their 'invisible' disorder.

The sessions take place on weekdays and, where possible, during school hours to accommodate those with child care responsibilities. Sessions are held in easily accessible community venues and GP practices.

Participants are asked to complete a paper headache diary for a period of up to eight weeks prior to the two-day programme. A one-to-one, individually tailored nurse-led consultation follows the two-day programme. During this session the nurse classifies the participant's headache type, discuss medication and lifestyle factors and explore participants' goals. This discussion is backed up by written information (for patient and GP), consistent with NICE guidance, to support shared informed decision making between the patient and their GP, about medication choices.[21] All participants will be offered telephone follow-up for up to eight weeks. The frequency of these follow-up calls will be individually negotiated and agreed with participants during the one-to-one consultation. Full details of the intervention content have been published elsewhere.[19]

Control intervention

Previous studies of this nature have reported that a usual care control arm was not an incentive to join the study.[11] We know from experience that patients enjoy the relaxation part of pain self-management programmes.[13] Therefore as an incentive to participate, the control group receive standard usual care plus a relaxation CD and instructions for use over the duration of the study, up until final follow-up at 12 months. Participants are free to continue using the relaxation CD thereafter should they wish.

For peer review only - http://bmjopen.bmj.com/site/about/guidelines.xhtml

As all participants in the trial have a headache classification interview prior to randomisation, we feed back the results of that classification interview to control participants, and their GPs, together with advice on headache management. In this way we ensure all participants receive best usual care.

The relaxation CD or downloadable MPG file (from the CHESS website) consists of a 17-minute relaxation audio script that starts by focusing on the breath before talking the participant through progressive relaxation of muscles. The participants in the control group choose when, where and how often they do the relaxation but are advised to try it two to three times a week, or as often as they feel appropriate over the course of the study.

Facilitator recruitment and training

All group sessions have two facilitators, one of whom must be a registered nurse, the other facilitator is any allied health care professional, registered with a regulatory body, with an interest in this patient population and condition. Facilitators were recruited via advertisements in allied health care profession organisations and trained over a two-day period to deliver the self-management intervention. Nurses received a further day of training to cover the one-to-one consultations. The training course covers the content of the intervention, facilitation skills, managing groups and trial procedures. Those undergoing the training were assessed using a learning assessment form at the end of the training course to check their knowledge and understanding. Those evaluated to be competent were asked to deliver the intervention. Those who either struggled with some elements of the content or expressed any concerns about delivering the intervention were provided with additional one to one support by the trainers.

Intervention quality assessment

Quality assurance of the intervention is important to ensure that the trial intervention is delivered consistently and in accordance with the trial protocol and manuals. As part of the intervention development we have produced two very detailed manuals, the first which covers the two days of group sessions and the second which details the process for the one to one consultations. All facilitators have been appropriately trained on the delivery of the intervention in accordance with the manuals. They have been instructed to use the manuals to guide them through delivery of the intervention.

To quality assure the course delivery we will aim to observe each facilitator. We will observe each facilitator early in their facilitation to allow any difficulties or challenges to be addressed. Subsequently they will be observed mid-way through. Observations will be by session and the number of sessions observed will depend on the ability of the observer to capture the required information.

The observations will be conducted by members of the CHESS team who have knowledge of the intervention and its delivery. Observers will complete an observation form which will address facilitator skills and adherence to content delivery. Feedback will be provided on the day where possible. If this is not possible the observer will arrange to contact the facilitator by phone. The study team will discuss any difficulties with the facilitator to minimise impact on the rest of the course and to help with the delivery of future courses. For any facilitators struggling the central research team will monitor their personal reflections for the remaining duration of the course and follow-up with phone calls if required.

Outcome measures - Primary outcome measure; headache related quality of life

Our primary outcomes is headache related quality of life assessed at the primary endpoint, 12 months after randomisation. For sample size determination we have specified the Headache Impact Test (HIT-6), a 6-item patient-reported outcome measure (PROM), as our measure of the primary outcome.[22] The HIT-6 provides a short overall assessment of headache impact – with items assessing fatigue, pain,

For peer review only - http://bmjopen.bmj.com/site/about/guidelines.xhtml

social functioning, emotional well-being and cognition. Prior to selecting our outcome measures, we did a systematic review of patient reported outcome measures for headaches.[23] Only for the HIT-6 did we find acceptable evidence supporting its use for our target population who may not have been given a headache diagnosis prior to study entry.

Another measure, shortlisted in the review, was the 14-item Migraine-Specific Quality of Life Questionnaire (MSQ v2.1) assesses the role restrictions, limitations and emotional impact of migraine.[23] The MSQv2.1 has acceptable evidence of psychometric properties following completion in a migraine population.[23-26]

To inform our selection of outcome measures we undertook interviews (n=14) with people with chronic headache. Our participants described greater perceived relevance of the MSQ(v2.1) compared to the HIT-6. With permission from GSK, the copyright holders, we modified the MSQ (v2.1), changing the focus from 'migraine' to 'headache'; the modified measure was renamed as the 'Chronic Headache Quality of Life Questionnaire' (CHQLQ v1.0). In our ongoing work we are assessing the performance of this modified instrument. In the event its performance is superior to the HIT-6 we will consider changing the primary outcome.

Both the HIT-6 and the CHQLQ v1.0 will be collected via postal questionnaire at baseline, four, eight and 12 months post randomisation.

Outcome measures - other secondary outcomes measures

The following secondary outcome measures are being collected:

1. *Headache days:* Our main outcome describing headaches days will be headaches days in the preceding month reported at baseline and follow-up postal questionnaires. We will also estimate total headaches days over the whole study period from patient reported data collected via a smartphone app.
2. *Headache impact:* We will assess headache impact based on their typical duration and severity reported in baseline and follow-up questionnaires
3. *Composite headache outcome:* We will produce a composite headache outcome of headache days x severity x duration using data from the smartphone app and questionnaires.
4. *Generic health-related quality of life*: We have included two standard measures of health-related quality of life – the SF-12 V2 and EQ-5D-5L.[27-29] There is limited, but acceptable, evidence supporting the use of the SF-12 V2 to assess overall quality of life in a headache population.[23] There is no such evidence for the EQ-5D-5L.[23] We will therefore, use EQ-5D-5L primarily for our health economic analyses.[28]
5. *Emotional well-being:* Hospital Anxiety and Depression Scale (HADs) - Psychological distress is extremely common in people living with chronic pain. HADs has been used in many previous studies of chronic pain, including the COPERS study where we identified positive effects on both anxiety and depression in a chronic pain population.[18 30]
6. *Self-Efficacy:* Pain Self-Efficacy Questionnaire (PSEQ) - Self-efficacy is an important mediator for how self-management interventions may improve patient outcomes. It is important, therefore, to measure change in self-efficacy as part of understanding the causal pathway for any change and informing our process evaluation.[31] We have previously reviewed measures of self-efficacy and concluded that PSEQ is the most appropriate choice for studies of this nature; although all current measures have limitations.[32]
7. *Social Activity: Social Integration Subscale of the Health Education Impact Questionnaire (heiQ) -* Chronic headache can result in a disrupted lifestyle and a reduced quality of life both during and between attacks; the impact of chronic headache on an individual's ability to commit to social plans is an important aspect of quality of life. Successful treatment should seek to improve both overall quality of life, as well as an individual's quality of life during the

For peer review only - http://bmjopen.bmj.com/site/about/guidelines.xhtml

attack, including their ability to integrate in society. A well-developed, condition-specific measure should seek to capture these distinctions. The five-item Social Integration Subscale (SIS) is one of eight domains contained within the heiQ, a measure of the impact of patient education programmes in chronic conditions.[33]

8. *Bodily pain:* Chronic headache commonly co-exists with other chronic painful disorders such as low back pain.[34-37] The CHESS intervention might affect the troublesomeness of other bodily pains. We will collect these data using a previously validated Troublesomeness Grid.[38]

At baseline we also collect data on age, gender, ethnic group, age at leaving full time education and current work status.

We will collect follow-up data at four, eight, and 12 months after randomisation by postal questionnaire survey. A £5 high street voucher is enclosed as a token of our appreciation at each initial time point. We will send out two postal follow-up reminders. In the event that no response is obtained, we will aim to collect our primary clinical outcome data by telephone. This includes the HIT-6 and EQ-5D-5L.

The smartphone data will be collected weekly for six months from initial eligibility and then monthly until 12 months after randomisation. A paper version of the app will be available to those that do not use a smartphone.

If there are missing data (for our key clinical outcomes), this will be followed up with the participant who completed the form, as soon as possible by a member of the team over the phone. We will phone the participant and enter the correct information onto the form, this will be initialled and dated.

Participant timelines

Figure 1 shows the recruitment flow and table 2 shows the study timelines.

Figure 1 – here

Table 2: Schedule of enrolment, interventions, and assessments

Activity	Pre-enrolment to allocation	Randomisation to 12 weeks	4 months	8 months	12 months
Expression of interest	X				
Trial information	X				
Download app to record headache patterns (weekly for six months and then monthly until the end of follow-up at 12 months)	X	X	X	X	X
Questionnaire Demographic information, HIT-6, CHQLQ, headache days, SF12, EQ-5D-5L, HADs, PSEQ, HeiQ, troublesomeness	X				
Consent	X				
Headache classification interview	X				
Allocation		X			

For peer review only - http://bmjopen.bmj.com/site/about/guidelines.xhtml

Intervention plus completion of paper headache diary for duration of up to eight weeks before attending group		X			
Control		X			
HIT-6, CHQLQ, headache days, SF12, EQ-5D-5L, HADs, PSEQ, HeiQ, troublesomeness			X		
2 postal follow up reminders and phone call for PO if needed			X		
HIT-6, CHQLQ, headache days, SF12, EQ-5D-5L, HADs, PSEQ, HeiQ, troublesomeness				X	
2 postal follow up reminders and phone call for PO if needed				X	
HIT-6, CHQLQ, headache days, SF12, EQ-5D-5L, HADs, PSEQ, HeiQ, troublesomeness					X
2 postal follow up reminders and phone call for PO if needed					X

HIT-6 (Headache Impact Test),[22] CHQLQ (Chronic Headache Quality of Life Questionnaire), SF12 (Short Form 12-item Health Survey),[27 29] EQ-5D-5L (EuroQoL),[28] HADs (Hospital Anxiety and Depression Scale),[30] PSEQ (Pain Self-Efficacy Questionnaire),[31] HeiQ (Health Education Impact Questionnaire)[33]

Sample size

For the purposes of our sample size calculation, the primary clinical outcome is the difference in mean HIT-6 score at 12 months post randomisation between the self-management group programme and the usual care relaxation therapy (control arm). The HIT-6 outcome measure is a continuous scale with higher values indicating more severe impact on daily life. From our systematic reviews, we anticipate a worthwhile difference to be 2.0, i.e. mean outcome in the control arm is 2.0 units higher than for the intervention.[23 39] In our feasibility study (N=114), the standard deviation of the HIT-6 score at baseline was 6.87.[40]

Participants are randomised to either the self-management group or usual care and relaxation therapy. In this design, there may be a clustering effect in the self-management group and not in the control arm, which needs to be allowed for in the sample size calculation. Based on similar trials we assume that the intra-class correlation coefficient (ICC) is 0.01.[18] The anticipated average size of the self-management programme is 10.

The minimum sample size required was estimated using Moerbeek and Wong's method to account for grouping in one arm.[41] To detect a between-group difference of 2.0 with standard deviation of 6.9 (a standardised mean difference of 0.29) and assuming that the ratio of the total variance in the self-management group to the relaxation therapy is 1 at the two-sided 5% significance level and at least 90% power, the sample size required is 523 participants (253 in the relaxation group and 270 in the self-management group).

In the feasibility study, the overwhelming majority of those recruited, approximately 95%, chronic migraine; just 5% had chronic tension type headache. We want to be able to draw definite conclusions on the specific subgroup of chronic migraine. Therefore, we will base our sample size and primary clinical outcome on the population with chronic migraine. Therefore, based on 95% of our sampled population with chronic migraine and accounting for a 20% loss to follow-up as above, the sample size we would require is 689 with 333 to the relaxation arm and 356 to the self-management programme.

In the feasibility study we recruited around 1/1,000 of practice population to take part,[40] we therefore anticipate we need to recruit from around 100 general practices with a combined list size of around 700,000.

For peer review only - http://bmjopen.bmj.com/site/about/guidelines.xhtml

In consultation with the Data Monitoring and Ethics Committee (DMEC), we will review the sample size around halfway through recruitment to ensure we have recruited sufficient participants with chronic migraine and revise our estimates using within trial data on the variance of our primary outcome at baseline. This review will be based on the headache classification and actual baseline standard deviation of our sampled population. We might also need to recruit some additional participants to ensure that the final group sessions at each site are adequately populated.

Recruitment

Participants are identified and invited into the study in two ways. Firstly, practices run electronic searches on their databases, to identify people who have consulted with headaches or have been prescribed migraine specific drugs (e.g. triptans, pizotifen) in the preceding two years. Practices then screen the lists for those it would be inappropriate to approach (e.g. poorly controlled serious mental illness, terminal illness, or known secondary causes of headache such as primary or secondary brain tumours), and send approach letters to the remainder.

People can self-refer to the trial. Participating general practices, the principal pharmacies used by their patients, are supplied with a study poster for display in patient areas inviting people to contact the study team if they have headaches and are interested in participating. Information about the trial is made available on the poster (in general practices and pharmacies), and websites (https://warwick.ac.uk/fac/med/research/ctu/trials/other/chess/ and https://warwick.ac.uk/fac/med/research/ctu/trials/chess. People who find about the trial through the internet or following media exposure, and can travel to sessions, can also self-refer to the trial.

Using both approaches will allow people receiving GP treatment for chronic headaches who are not coded in the GP system as having headaches, and those who are self-medicating their headaches, the opportunity to join the study. We anticipate that we will primarily recruit people registered with participating practices; however, we will not restrict recruitment to those registered with participating practices.

In addition to these two main recruitment strategies, a study press release was submitted in January 2018 which was picked up by various media outlets and generated further interest in the study from potential participants.

The study co-ordinating team contact people expressing an interest in the study and check that they are eligible, explain the study, and obtain participant's verbal consent to start completing an electronic headache symptom severity, duration & frequency diary (or paper version where there is no access to the internet). The electronic diary is completed weekly for the first six months and subsequently monthly until the end of follow-up at 12 months. The electronic diary is used to identify any early effects of rebound headache in those with medication overuse headache. At this time participants are sent a baseline questionnaire and consent form to return in a freepost envelope.

Following receipt of baseline questionnaire and consent form participants are contacted by phone for a nurse headache classification interview. People whose headache is confirmed to meet our entry criteria are then eligible for randomisation. If the nurse has a concern that the potential participant may have another headache type there is a second interview with a doctor skilled in the management of headache disorders from the National Migraine Centre (http://www.nationalmigrainecentre.org.uk/). This is to ensure no one is inappropriately excluded and that people with a headache type needing more specific treatment are directed towards appropriate treatment. For example, 2/108 people in our feasibility study had cluster headaches.[40]

For peer review only - http://bmjopen.bmj.com/site/about/guidelines.xhtml

In such cases we write to the GP and the participant with details on the excluded headache type and explain that they no longer fit the inclusion criteria for the CHESS trial.

2. Assignment of interventions

Randomisation

The randomisation is stratified by geographical locality (Midlands and Greater London) and headache type (six possible headache types: chronic tension type headache, probable chronic migraine and definite chronic migraine with or without medication overuse headache) using minimisation. Randomisation takes place using an online application specifically developed for the CHESS Study by the Warwick Clinical Trials Unit (WCTU) programming team.

Randomisation takes place when there are around 20 participants who would be able to attend a local group if randomised to the active intervention. Typically these will come from 4-5 proximate general practices. However, people from other localities who are unable to attend a more local group and people who have self-referred to the study who are willing and able to travel to the sessions will also be included. By randomising in groups around two weeks prior to the start of a group, we are able to minimise any delay between randomisation and start of the intervention and ensure everyone has adequate follow-up from when they might have been exposed to the intervention. For those that are subsequently unable to attend after randomisation to a group, we will offer them another group where possible.

Allocation concealment and protection from bias

Allocation concealment will be maintained by using Warwick CTU's centralised randomisation service. All baseline data will be collected prior to randomisation. Blinding will be impossible for participants, facilitators and the central co-ordinating CHESS team.

Our primary outcome is a participant completed outcome. Inevitably, participants will be aware of their treatment allocation. We will develop and sign off a detailed pre-specified statistical analysis plan before allocation codes are released.

3. Data collection, management and analysis

Data collection methods

Data will be collected using two methods, postal survey questionnaires and a smartphone application (App) for headaches. All participants will be asked to complete a smartphone app about their headaches. If they do not have access to a smartphone, or do not wish to use the app, a paper copy will be provided. Participants will initially complete the app weekly for up to six months, to cover any period of withdrawal from medication, then monthly thereafter (still requiring them to reflect over the previous 7 days) until the end of the study at 12 months after randomisation. We are working with Clinvivo Ltd a University of Warwick spin-out company specialising in electronic data collection, to capture data on headache frequency, duration and severity electronically using a smartphone App (Table 3 shows the questions completed by participants and mode of response required). The data from the questions in the electronic diary will be numerical and downloaded into a WCTU database using a randomly generated 256-bit AES (Advanced Encryption Standard) key. At the end of the participants 12 months they will be provided with a summary report of their headache application data.

For peer review only - http://bmjopen.bmj.com/site/about/guidelines.xhtml

A daily detailed paper headache diary is kept only by the intervention arm participants and the data will not be analysed by the study team but kept as a record by the participants and used during the one to one consultation where it will be collected and kept by the research team.

Table 3. Questions completed on the smartphone app.

Question	Mode of response
On how many of the last 7 days have you had a headache?	Insert number of headache days
On those days you had a headache, on average how long did they last?	Insert number of hours
On those days you had a headache on average how severe were they?	Scale of 0 to 10 (with 0 being no pain and 10 extremely severe pain)

Data management

All questionnaire data received by the trial team are reviewed for completeness and entered onto a secure, backed-up bespoke database held at WCTU which will be accessible only by authorised members of the team. Data from the smartphone App are downloaded securely (as described above) into the trial database. All data is checked when received, any queries relating to the HIT6 CHQLQ or EQ5D are raised with the participant by telephone.

Statistical analysis of effectiveness and harms

Our primary endpoint analyses will be based on the twelve month data.

Participants' characteristics and reported outcomes will be summarised as means and standard deviations (for continuous data) or frequencies and percentages (for categorical data) by treatment arms. Difference between baseline and the three follow-up time points (four-, eight- and 12-month post randomisation) will be computed for the primary and secondary outcomes by treatment arms.

The primary analysis approach will be intention to treat i.e. the data will be analysed according to the treatment the participant was originally allocated to, irrespective of what they actually received. We will explore the possibility of carrying out a complier averaged causal effect (CACE) analysis as a sensitivity analysis. Our primary analysis will be the difference between the self-management therapy (intervention) and the relaxation therapy (control) groups with a 95% confidence interval (CI) in the population with chronic migraine – if the proportion of participants with chronic tension type headache is ≤ 15%.The hypothesis testing of the primary outcome will be two-sided at the 5% level and the main analysis will estimate the treatment effect using a multilevel model to account for clustering (the model used to design this main trial). We will also present results for the whole population (all headache types). If the proportion of chronic tension type headache is >15% then the primary analysis will be according to the whole population of chronic headache (chronic migraine and tension type headache). Our experience is that NICE, was specifically interested in data on specific headache types; excluding data that reported mixed population of people with chronic headaches. We will, therefore in addition to our primary analyses present the results (mean difference and 95% CI) for each of the two headache types with or without medication overuse headache separately, and present results for those with or without medication overuse separately to facilitate future meta-analyses and inform future condition specific guidelines. All analyses will be adjusted for the randomisation stratification factors (types of headache and geographical locality), sex, age and the baseline value of the dependent variable.

For peer review only - http://bmjopen.bmj.com/site/about/guidelines.xhtml

Similar analyses will be performed for all the other secondary outcomes. Pre-specified subgroup analyses using formal statistical tests for interaction will examine whether baseline anxiety, depression and severity are moderators of treatment effect.[42] We will assess the level of missingness in the primary outcome and if required, we will use appropriate multiple imputation techniques to impute data and estimate the treatment effect as a form of sensitivity analysis. A full statistical analysis plan, including all primary and secondary analyses, will be written and signed off prior to releasing allocation codes to analysts and others involved in developing the plan.

Using the data from the smartphone App we will generate a composite score for headache impact over the one-year of follow up as the function of headaches days x average duration x average severity. Presenting these data graphically will allow any early benefits or harms from the intervention to be identified.

Health Economic Evaluation

Our economic evaluation will be conducted alongside the trial and we will initially adopt a one-year time horizon from both an NHS and personal social services perspective and, separately for the purpose of a sensitivity analysis a broader societal perspective, to estimate the cost-utility of the intervention. Resource use data will be collected to explore the costs of the delivery of the intervention and to estimate the key cost drivers. This will mainly consist of visits to the GP practice, medication usage and any adverse events or length of stay in the hospital. In terms of costs to society, we will estimate time off work and any productivity losses associated with chronic headaches. Resource use information will be collected using self-completed postal questionnaires completed at four, eight and 12 months after randomisation, as well as the use of routine health service data collected from general practice records. Resource input will be valued using national estimates of unit costs such as the Prescription Cost Analysis database or the Unit Costs of Health and Social Care compendium.[43] Preference-based health-related quality of life outcomes will primarily be assessed through the completion of the EQ-5D-5L at each follow-up point.[44] Quality-adjusted life-years (QALYs) will be calculated as the area under the baseline-adjusted utility curve, and will be calculated using linear interpolation between baseline and follow-up utility scores.

The results of the economic evaluation will be presented using incremental cost-effectiveness ratios, expressed in terms of incremental cost per QALY gained, and cost-effectiveness acceptability curves generated via non-parametric bootstrapping.

More extensive economic modelling using decision-analytic methods will extend the target population, the time horizon to five years as the long-term natural history is unclear and the decision context, drawing on best available information from the literature together with stakeholder consultations to supplement the trial data. Longer-term costs and consequences will be discounted to present values using nationally recommended discount rates recommended for health technology appraisal. We will use probabilistic sensitivity analysis to estimate the impact of uncertainty over model parameters. We will also use simple sensitivity analysis to assess the robustness of the results to changes in deterministic parameters such as medication dosages, costs, discount rate and time horizon for patients presenting with chronic headaches. We will also explore cost-effectiveness of the intervention by conducting subgroup analyses for the different headache types.

4. Monitoring, ethics and dissemination

Programme steering committee (PSC)

We have established a PSC to oversee the whole programme of work. The PSC functions specifically as a trial steering committee specifically for this trial. The PSC consists of seven members including

For peer review only - http://bmjopen.bmj.com/site/about/guidelines.xhtml

two patient representative. The PSC will be responsible for oversight and monitoring and will consider any information from surveillance activity of other research in the field.

Data monitoring and Ethics Committee (DMEC)
A data monitoring and ethics committee has been convened consisting of an independent statistician, a trialist, a clinician and a lay member. Confidential reports that summarise the trial data and safety data will be reviewed by the DMC. The DMC will advise the PSC as to whether to continue, amend or terminate the trial based primarily on safety and efficacy considerations

Harms / Adverse Events (AE)
An adverse event or serious adverse event is any event that takes place on the way to, during, or on the way home from the intervention course. This includes the two days of the group course and the one to one nurse appointment. Our experience across multiple studies of group interventions is that adverse events that are directly attributable to the intervention are rare.

Events during the session, such as severe psychological disturbance, any mild or moderate levels of emotional distress as a result of discussing experiences of living with chronic headache, a fall during travel to and from the venue or any other adverse events, occurring during the delivery of the intervention will be reported to the trial co-coordinator by the intervention team. Any short-term increase in headaches as a consequence of medication withdrawal will be captured using the smartphone app (or paper version for those without access to a smartphone).

All adverse events will be managed in line with Warwick CTU's standard operating procedures.

Process Evaluation
A separate mixed-methods process evaluation is running alongside the trial. This will explore both the process of implementation the trial and the process of delivering and receiving the intervention. The process evaluation protocol is reported elsewhere.[45]

The fidelity of the delivery of the intervention will be assessed by this team in three ways, through: audio recording of the groups, facilitator reflection and participant feedback. This will assess facilitator competence, adherence to the course manual and trial procedures and participant interaction and engagement.

Patient and Public Involvement (PPI)
We have had substantial patient and public involvement in the feasibility study prior to finalising this protocol. Lay members were involved in the development of the classification interview, development of the intervention, PROM selection, and conduct and management of the study via the independent programme steering and trial management group.

Our trial management group includes our lay co-applicants who are representatives of three leading UK migraine charities (The Migraine Trust, Migraine Action (before their merger with Migraine Trust), and National Migraine Centre).

We have developed a lay steering group who are and will be collaboratively involved during the study. At key points in the programme we will approach the lay steering group for input, this will include at the end of the trial when results are disseminated.

ETHICS AND DISSEMINATION

The University of Warwick (Research Impact Services, University of Warwick, Coventry CV4 7AL) are the Sponsor for the study.

For peer review only - http://bmjopen.bmj.com/site/about/guidelines.xhtml

Ethics approval was provided by: North West - Greater Manchester East Research Ethics Committee (REC REF: 16/NW/0890) The trial is conducted in accordance with the principles outlined in the Declaration of Helsinki and conforms to GCP and SOPs set-out by the WCTU. As reported above we have convened a PSC and DMC to oversee the trial.

All identifiable data is anonymised and treated as confidential. Participants are informed that they are free to withdraw at any time during any phase of the work.

The findings from this trial will be disseminated via a final report to the NIHR, presentations at conferences and publications in high quality peer-reviewed journals.

For the healthcare professionals involved in the study we will disseminate results of the study through the study website. We will also host a meeting to present the trial results to commissioners and clinicians. For the participants, we will provide a written lay summary of the findings and also publish these on a study specific website; with contact information should they wish to discuss the findings. Our charity partners will be involved with feedback to the organisations they represent.

At the time of writing (08/08/2019) a total of 736 participants have been randomised (380 to the intervention arm and 356 to the control arm) and the trial is now in the follow-up phase.
A comprehensive programme of preparatory work has allowed this substantial trial of an education and self-management intervention for people living with chronic headaches to be developed. There is a clear interest in taking part in the trial. Our follow up will be complete in early 2020 and the final results published in 2021.

For peer review only

For peer review only - http://bmjopen.bmj.com/site/about/guidelines.xhtml

REFERENCES

1. Global, regional, and national burden of neurological disorders during 1990-2015: a systematic analysis for the Global Burden of Disease Study 2015. The Lancet. Neurology 2017;**16**(11):877-97.
2. Steiner TJ, Scher AI, Stewart WF, Kolodner K, Liberman J, Lipton RB. The prevalence and disability burden of adult migraine in England and their relationships to age, gender and ethnicity. Cephalalgia : an international journal of headache 2003;**23**(7):519-27.
3. Stovner L, Hagen K, Jensen R, et al. The global burden of headache: a documentation of headache prevalence and disability worldwide. Cephalalgia : an international journal of headache 2007;**27**(3):193-210.
4. Castillo J, Munoz P, Guitera V, Pascual J. Epidemiology of chronic daily headache in the general population. Headache 1999;**39**(3):190-6.
5. Lu SR, Fuh JL, Chen WT, Juang KD, Wang SJ. Chronic daily headache in Taipei, Taiwan: prevalence, follow-up and outcome predictors. Cephalalgia : an international journal of headache 2001;**21**(10):980-6.
6. Wang SJ, Fuh JL, Lu SR, et al. Chronic daily headache in Chinese elderly: prevalence, risk factors, and biannual follow-up. Neurology 2000;**54**(2):314-9.
7. Mehuys E, Paemeleire K, Van Hees T, et al. Self-medication of regular headache: a community pharmacy-based survey. European journal of neurology 2012;**19**(8):1093-9.
8. Probyn K, Bowers H, Caldwell F, et al. Prognostic factors for chronic headache: A systematic review. Neurology 2017;**89**(3):291-301.
9. Nichols VP, Ellard DR, Griffiths FE, Kamal A, Underwood M, Taylor SJC. The lived experience of chronic headache: a systematic review and synthesis of the qualitative literature. BMJ open 2017;**7**(12):e019929.
10. Probyn K, Bowers H, Mistry D, et al. Non-pharmacological self-management for people living with migraine or tension-type headache: a systematic review including analysis of intervention components. BMJ open 2017;**7**(8):e016670.
11. Carnes D, Homer K, Underwood M, Pincus T, Rahman A, Taylor SJ. Pain management for chronic musculoskeletal conditions: the development of an evidence-based and theory-informed pain self-management course. BMJ open 2013;**3**(11):e003534.
12. Pinnock H, Parke HL, Panagioti M, et al. Systematic meta-review of supported self-management for asthma: a healthcare perspective. BMC medicine 2017;**15**(1):64.
13. Taylor SJC, Pinnock H, Epiphaniou E, et al. Health Services and Delivery Research. A rapid synthesis of the evidence on interventions supporting self-management for people with long-term conditions: PRISMS - Practical systematic Review of Self-Management Support for long-term conditions. Southampton (UK): NIHR Journals Library, 2014.
14. Carville S, Padhi S, Reason T, Underwood M. Diagnosis and management of headaches in young people and adults: summary of NICE guidance. BMJ (Clinical research ed.) 2012;**345**:e5765.
15. Chan AW, Tetzlaff JM, Altman DG, et al. SPIRIT 2013 statement: defining standard protocol items for clinical trials. Annals of internal medicine 2013;**158**(3):200-7.
16. Louter MA, Ward TN, Terwindt GM. Chronic headache: What's in a name? Neurology 2017;**89**(3):224-25.
17. Potter R, Hee SW, Griffiths F, et al. Development and validation of a telephone classification interview for common chronic headache disorders. J Headache Pain 2019;**20**(1):2.
18. Taylor SJC, Carnes D, Homer K, et al. Programme Grants for Applied Research. Improving the self-management of chronic pain: COping with persistent Pain, Effectiveness Research in Self-management (COPERS). Southampton (UK): NIHR Journals Library, 2016.
19. Patel S, Potter R, Matharu M, et al. Development of an education and self-management intervention for chronic headache - CHESS trial (Chronic Headache Education and Self-management Study). J Headache Pain 2019;**20**(1):28.

For peer review only - http://bmjopen.bmj.com/site/about/guidelines.xhtml

20. Taylor SJ, Carnes D, Homer K, et al. Novel Three-Day, Community-Based, Nonpharmacological Group Intervention for Chronic Musculoskeletal Pain (COPERS): A Randomised Clinical Trial. PLoS medicine 2016;**13**(6):e1002040.
21. NICE. Headaches in over 12s: diagnosis and management - Clinical guideline [CG150], 2012.
22. Kosinski M, Bayliss MS, Bjorner JB, et al. A six-item short-form survey for measuring headache impact: the HIT-6. Quality of life research : an international journal of quality of life aspects of treatment, care and rehabilitation 2003;**12**(8):963-74.
23. Haywood KL, Mars TS, Potter R, Patel S, Matharu M, Underwood M. Assessing the impact of headaches and the outcomes of treatment: A systematic review of patient-reported outcome measures (PROMs). Cephalalgia : an international journal of headache 2018;**38**(7):1374-86.
24. Jhingran P, Osterhaus JT, Miller DW, Lee JT, Kirchdoerfer L. Development and validation of the Migraine-Specific Quality of Life Questionnaire. Headache 1998;**38**(4):295-302.
25. Martin BC, Pathak DS, Sharfman MI, et al. Validity and reliability of the migraine-specific quality of life questionnaire (MSQ Version 2.1). Headache 2000;**40**(3):204-15.
26. McKenna SP, Doward LC, Davey KM. The Development and Psychometric Properties of the MSQOL: A Migraine-Specific Quality-of-Life Instrument. Clinical drug investigation 1998;**15**(5):413-23.
27. Gandek B, Ware JE, Aaronson NK, et al. Cross-validation of item selection and scoring for the SF-12 Health Survey in nine countries: results from the IQOLA Project. International Quality of Life Assessment. Journal of clinical epidemiology 1998;**51**(11):1171-8.
28. Herdman M, Gudex C, Lloyd A, et al. Development and preliminary testing of the new five-level version of EQ-5D (EQ-5D-5L). Quality of life research : an international journal of quality of life aspects of treatment, care and rehabilitation 2011;**20**(10):1727-36.
29. Jenkinson C, Layte R, Lawrence K. Development and testing of the Medical Outcomes Study 36-Item Short Form Health Survey summary scale scores in the United Kingdom. Results from a large-scale survey and a clinical trial. Medical care 1997;**35**(4):410-6.
30. Zigmond AS, Snaith RP. The hospital anxiety and depression scale. Acta psychiatrica Scandinavica 1983;**67**(6):361-70.
31. Nicholas MK. The pain self-efficacy questionnaire: Taking pain into account. European journal of pain (London, England) 2007;**11**(2):153-63.
32. Miles CL, Pincus T, Carnes D, Taylor SJ, Underwood M. Measuring pain self-efficacy. The Clinical journal of pain 2011;**27**(5):461-70.
33. Osborne RH, Elsworth GR, Whitfield K. The Health Education Impact Questionnaire (heiQ): an outcomes and evaluation measure for patient education and self-management interventions for people with chronic conditions. Patient education and counseling 2007;**66**(2):192-201.
34. Hagen K, Linde M, Steiner TJ, Zwart JA, Stovner LJ. The bidirectional relationship between headache and chronic musculoskeletal complaints: an 11-year follow-up in the Nord-Trondelag Health Study (HUNT). European journal of neurology 2012;**19**(11):1447-54.
35. Jimenez-Sanchez S, Fernandez-de-Las-Penas C, Carrasco-Garrido P, et al. Prevalence of chronic head, neck and low back pain and associated factors in women residing in the Autonomous Region of Madrid (Spain). Gaceta sanitaria 2012;**26**(6):534-40.
36. Plesh O, Adams SH, Gansky SA. Self-reported comorbid pains in severe headaches or migraines in a US national sample. Headache 2012;**52**(6):946-56.
37. Yoon MS, Manack A, Schramm S, et al. Chronic migraine and chronic tension-type headache are associated with concomitant low back pain: results of the German Headache Consortium study. Pain 2013;**154**(3):484-92.
38. Parsons S, Carnes D, Pincus T, et al. Measuring troublesomeness of chronic pain by location. BMC musculoskeletal disorders 2006;**7**:34.
39. Cousins S, Ridsdale L, Goldstein LH, Noble AJ, Moorey S, Seed P. A pilot study of cognitive behavioural therapy and relaxation for migraine headache: a randomised controlled trial. Journal of neurology 2015;**262**(12):2764-72.

For peer review only - http://bmjopen.bmj.com/site/about/guidelines.xhtml

40. White K, Potter R, Patel S, et al. Chronic Headache Education and Self-management Study (CHESS) - a mixed method feasibility study to inform the design of a randomised controlled trial. Submitted. 2019;**19**(30).
41. Moerbeek M, Wong WK. Sample size formulae for trials comparing group and individual treatments in a multilevel model. Statistics in medicine 2008;**27**(15):2850-64.
42. Brookes ST, Whitely E, Egger M, Smith GD, Mulheran PA, Peters TJ. Subgroup analyses in randomized trials: risks of subgroup-specific analyses; power and sample size for the interaction test. Journal of clinical epidemiology 2004;**57**(3):229-36.
43. Authority NBS. Prescription Cost Analysis (PCA) data. Secondary Prescription Cost Analysis (PCA) data. https://www.nhsbsa.nhs.uk/prescription-data/dispensing-data/prescription-cost-analysis-pca-data.
44. van Hout B, Janssen MF, Feng YS, et al. Interim scoring for the EQ-5D-5L: mapping the EQ-5D-5L to EQ-5D-3L value sets. Value in health : the journal of the International Society for Pharmacoeconomics and Outcomes Research 2012;**15**(5):708-15.
45. Nichols VP, Ellard DR, Griffiths FE, Underwood M, Taylor SJC, Patel S. The CHESS trial: protocol for the process evaluation of a randomised trial of an education and self-management intervention for people with chronic headache. Trials 2019;**20**(1):323.

For peer review only

For peer review only - http://bmjopen.bmj.com/site/about/guidelines.xhtml

ABBREVIATIONS

Abbreviation	Full form
CHESS	Chronic Headache Education and Self-management Study
HIT6	Headache Impact Test
MOH	Medication Overuse Headaches
NICE	National Institute for Health and Care Excellence
RCT	Randomised Controlled Trial
GP	General Practitioner
PPI	Patient and Public Involvement
PROMs	Patient-Reported Outcome Measures
MSQ	Migraine-Specific Quality of Life Questionnaire
CHQLQ	Chronic Headache Quality of Life Questionnaire
SF12	Short Form 12-item Health Survey
EQ-5D-5L	EuroQoL
HADs	Hospital Anxiety and Depression Scale
PSEQ	Pain Self-Efficacy Questionnaire
HeiQ	Health Education Impact Questionnaire
ICC	intra-class correlation coefficient
DMEC	Data Monitoring and Ethics Committee
WCTU	Warwick Clinical Trials Unit
App	Application
CACE	Complier Averaged Causal Effect
CI	Confidence Interval
QALYs	Quality-Adjusted Life-Years
PSC	Programme Steering Committee
AE	Adverse Event
DCM	Definite Chronic Migraine
PCM	Probable Chronic Migraine
TTH	Tension Type Headache
MOH	Medication Overuse Headache

DECLARATIONS

Ethics approval and consent to participate
Ethics approval was given on the 17th Of February 2017 by North West - Greater Manchester East Research Ethics Committee (REC REF: 16/NW/0890). Written consent was taken for participation.

Competing interests statements
MU is a director and shareholder of Clinvivo Ltd. Use of this company was specified in original application for funding to NIHR. MU has recused himself from all discussion regarding the use of the App in this study. All contracting processes have been in accord with University of Warwick financial regulations.

MU was Chair of the NICE accreditation advisory committee until March 2017 for which he received a fee. He is chief investigator or co-investigator on multiple previous and current research grants from the UK National Institute for Health Research, Arthritis Research UK and is a co-investigator on grants funded by Arthritis Australia and Australian NHMRC. He has received travel expenses for speaking at conferences from the professional organisations hosting the conferences He is part of an academic

For peer review only - http://bmjopen.bmj.com/site/about/guidelines.xhtml

partnership with Serco Ltd related to return to work initiatives. He is an editor of the NIHR journal series, and a member of the NIHR Journal Editors group for which he receives a fee. He has published multiple papers on chronic pain some of which are referenced in this paper.

MSM serves on the advisory board for Allergan, Medtronic, Novartis and TEVA and has received payment for the development of educational presentations from Allergan, electroCore, Medtronic, Novartis and TEVA.

SP and HS are directors of Health Psychology Services Ltd which in part provides psychological treatments for those with chronic pain.

Funding statement
This research was funded by the NIHR Programme Grants for Applied Research programme (RP-PG-1212-20018). The views expressed in this publication are those of the author(s) and not necessarily those of the NHS, the NIHR or the Department of Health.

Data access
The final trial dataset will be available for sharing with other research teams after publication of the main trial paper. All data will be anonymised and provided after assessment of any further research proposals and evidence of ethical approvals.

Authors contribution
SP - study concept and design, supported writing of the first draft of the manuscript and finalised the manuscript for submission.
DC – study concept and design, supported writing of the first draft of the manuscript, provided critical revisions to the manuscript
SE - study concept and design, provided critical revisions to the manuscript
DE - study concept and design, provided critical revisions to the manuscript
FG - study concept and design, provided critical revisions to the manuscript
KH - study concept and design, provided critical revisions to the manuscript
SWH - study concept and design, provided critical revisions to the manuscript
DM - study concept and design, provided critical revisions to the manuscript
HM - study concept and design, provided critical revisions to the manuscript
VN - study concept and design, provided critical revisions to the manuscript
SPe - study concept and design, provided critical revisions to the manuscript
TP - study concept and design, provided critical revisions to the manuscript
RP - study concept and design, provided critical revisions to the manuscript
HS - study concept and design, provided critical revisions to the manuscript
SJT - study concept and design, provided critical revisions to the manuscript
MU – conceived the original study design and supported writing of the first draft of the manuscript
KW - study concept and design, provided critical revisions to the manuscript
MSM - study concept and design, provided critical revisions to the manuscript

DC, SE, DE, FG, KH, SWH, HM, SPe, TP, HS, SJT, MU, MSM - original grant holders

All authors have read and approved the final manuscript.

Acknowledgements
The manuscript was written on behalf of the CHESS co-applicants, study team and PPI representatives.

For peer review only - http://bmjopen.bmj.com/site/about/guidelines.xhtml

Figure 1: Trial flow diagram

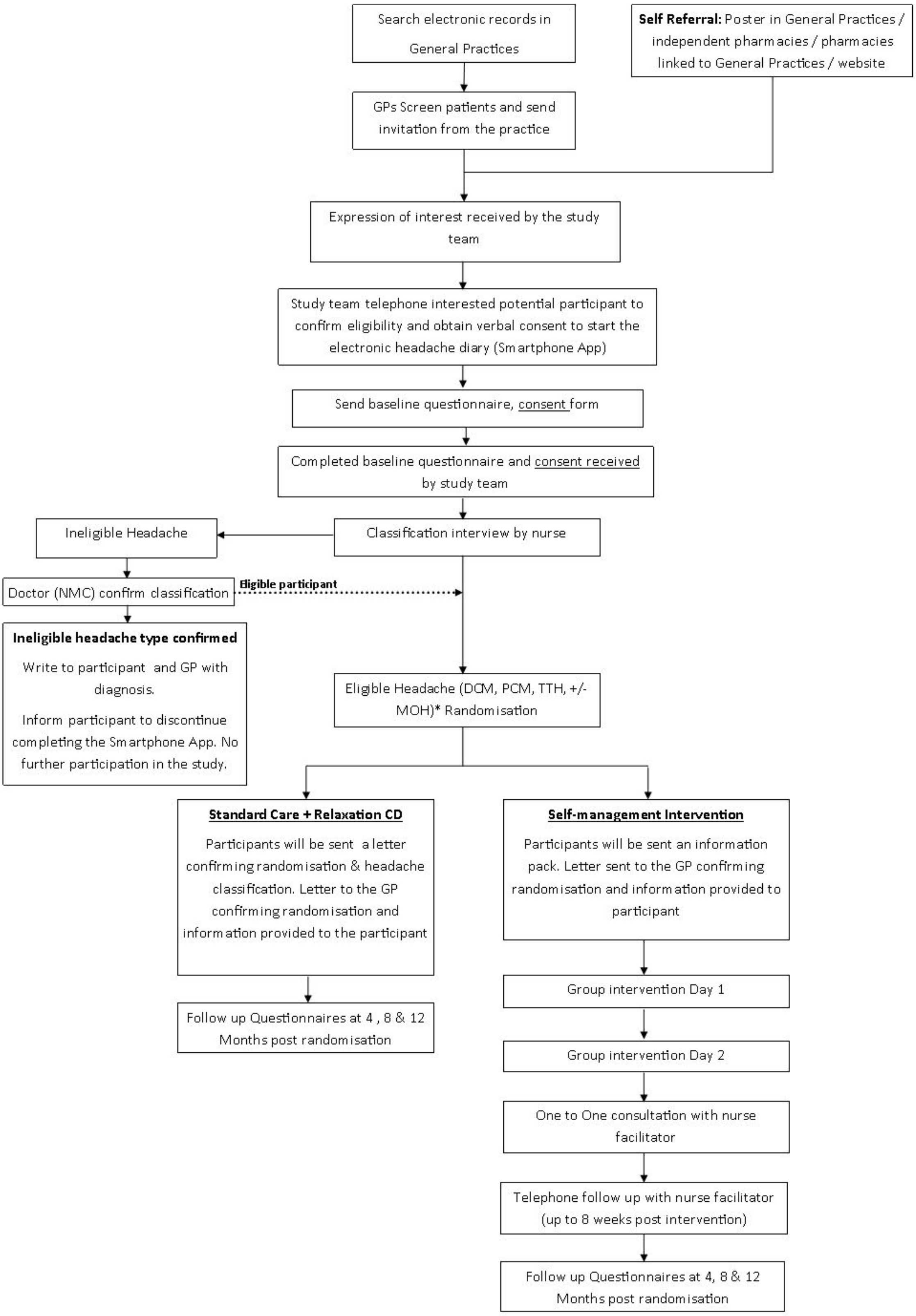

*DMC—Definite Chronic Migraine; PCM— Probable Chronic Migraine; TTH— Tension Type Headache; MOH— Medication Overuse Headache

CHESS
Chronic Headache Education and Self-management Study

WARWICK
CLINICAL TRIALS UNIT

CONSENT FORM

Study	Chronic Headache Education and Self-management Study (CHESS)	Name of Investigator	Prof Martin Underwood
Practice number		Participant ID number	

Please tick the boxes below if you agree ↓

1. I confirm that I have read the information sheet 28.Jun.2018, version 1.5 for the above study. I have had the opportunity to consider the information and ask questions and have had these answered satisfactorily.	☐
2. I understand that my participation is voluntary and that I am free to withdraw at any time without giving any reason, without my medical care or legal rights being affected.	☐
3. I agree for my contact details to be held at Warwick Clinical Trials Unit for the purpose of contacting me regarding the study processes or any study queries, to send me questionnaires and other study related material.	☐
4. I give permission for the study team to use data related to my headaches from my GP records, where it is relevant to my taking part in this research.	☐
5. I understand that all information that is collected during the study will be kept confidential at all times and held in compliance with the Data Protection Act 2018.	☐
6. I understand that relevant sections of my medical notes and data collected during the study, may be looked at by individuals from the research team at Warwick Clinical Trials Unit, from the regulatory authorities or from the NHS Trust, where it is relevant to my taking part in this research. I give permission for these individuals to have access to my records.	☐
7. I understand that the information collected about me will be used to support other research in the future, and may be shared anonymously with other researchers.	☐
8. I agree to my General Practitioner being informed of my participation in the study.	☐
9. I understand I will be asked to complete an electronic smartphone application diary developed by Clinvivo Ltd who are a third party provider; and that my data will be anonymous.	☐
10. I understand and agree that the self-management programme may be audio recorded and transcribed for quality control and process evaluation purposes. I understand transcripts will be held securely and only accessed by authorised study personnel.	☐
11. I understand that transcripts will be made anonymous by a member of the research team or a third party transcription service contracted to work on the study. I understand anonymised transcripts may be shared with other carefully selected researchers for further analyses.	☐
12. I understand I may subsequently be contacted by the research team to take part in an interview study as part of CHESS; and that I might be approached again after the completion of the study to take part in further headache research.	☐
13. I agree to take part in the above study.	☐

Name of Participant (PRINT NAME)		Signature of participant		Date signed	

When completed: *1 signed* ***original*** *for Investigator on site file (****ISF****), 1* ***copy*** *of signed original* ***to the participant, 1 copy*** *for participant Medical Record*

This letter presents independent research funded by the National Institute for Health Research (NIHR) under the Programme Grants for Applied Research programme [project number RP-PG-1212-20018]. The views expressed in this letter are those of the author(s) and not necessarily those of the NIHR or the Department of Health and Social Care. IRAS ID: 215304

FUNDED BY NIHR | National Institute for Health Research

For peer review only - http://bmjopen.bmj.com/site/about/guidelines.xhtml

Reporting checklist for protocol of a clinical trial.

Based on the SPIRIT guidelines.

Instructions to authors

Complete this checklist by entering the page numbers from your manuscript where readers will find each of the items listed below.

Your article may not currently address all the items on the checklist. Please modify your text to include the missing information. If you are certain that an item does not apply, please write "n/a" and provide a short explanation.

Upload your completed checklist as an extra file when you submit to a journal.

In your methods section, say that you used the SPIRITreporting guidelines, and cite them as:

Chan A-W, Tetzlaff JM, Altman DG, Laupacis A, Gøtzsche PC, Krleža-Jerić K, Hróbjartsson A, Mann H, Dickersin K, Berlin J, Doré C, Parulekar W, Summerskill W, Groves T, Schulz K, Sox H, Rockhold FW, Rennie D, Moher D. SPIRIT 2013 Statement: Defining standard protocol items for clinical trials. Ann Intern Med. 2013;158(3):200-207

		Reporting Item	Page Number
**Administrative information**			
Title	#1	Descriptive title identifying the study design, population, interventions, and, if applicable, trial acronym	1

For peer review only

Trial registration	#2a	Trial identifier and registry name. If not yet registered, name of intended registry	3
Trial registration: data set	#2b	All items from the World Health Organization Trial Registration Data Set	N/A
Protocol version	#3	Date and version identifier	3
Funding	#4	Sources and types of financial, material, and other support	22
Roles and responsibilities: contributorship	#5a	Names, affiliations, and roles of protocol contributors	1 & 2
Roles and responsibilities: sponsor contact information	#5b	Name and contact information for the trial sponsor	17
Roles and responsibilities: sponsor and funder	#5c	Role of study sponsor and funders, if any, in study design; collection, management, analysis, and interpretation of data; writing of the report; and the decision to submit the report for publication, including whether they will have ultimate authority over any of these activities	N/A
Roles and responsibilities: committees	#5d	Composition, roles, and responsibilities of the coordinating centre, steering committee, endpoint adjudication committee, data management team, and	15-17

For peer review only

		other individuals or groups overseeing the trial, if applicable (see Item 21a for data monitoring committee)	
**Introduction**			
Background and rationale	#6a	Description of research question and justification for undertaking the trial, including summary of relevant studies (published and unpublished) examining benefits and harms for each intervention	5
Background and rationale: choice of comparators	#6b	Explanation for choice of comparators	5
Objectives	#7	Specific objectives or hypotheses	5
Trial design	#8	Description of trial design including type of trial (eg, parallel group, crossover, factorial, single group), allocation ratio, and framework (eg, superiority, equivalence, non-inferiority, exploratory)	5
**Methods: Participants, interventions, and outcomes**			
Study setting	#9	Description of study settings (eg, community clinic, academic hospital) and list of countries where data will	6

For peer review only

		be collected. Reference to where list of study sites can be obtained	
Eligibility criteria	#10	Inclusion and exclusion criteria for participants. If applicable, eligibility criteria for study centres and individuals who will perform the interventions (eg, surgeons, psychotherapists)	6
Interventions: description	#11a	Interventions for each group with sufficient detail to allow replication, including how and when they will be administered	7
Interventions: modifications	#11b	Criteria for discontinuing or modifying allocated interventions for a given trial participant (eg, drug dose change in response to harms, participant request, or improving / worsening disease)	N/A
Interventions: adherance	#11c	Strategies to improve adherence to intervention protocols, and any procedures for monitoring adherence (eg, drug tablet return; laboratory tests)	8 & 16
Interventions: concomitant care	#11d	Relevant concomitant care and interventions that are permitted or prohibited during the trial	N/A
Outcomes	#12	Primary, secondary, and other outcomes, including the specific measurement variable (eg, systolic blood pressure), analysis metric (eg, change from baseline, final value, time to event), method of aggregation (eg, median, proportion), and time point for each outcome.	8-11

For peer review only

		Explanation of the clinical relevance of chosen efficacy and harm outcomes is strongly recommended	
Participant timeline	#13	Time schedule of enrolment, interventions (including any run-ins and washouts), assessments, and visits for participants. A schematic diagram is highly recommended (see Figure)	10 & FIG 1
Sample size	#14	Estimated number of participants needed to achieve study objectives and how it was determined, including clinical and statistical assumptions supporting any sample size calculations	11-12
Recruitment	#15	Strategies for achieving adequate participant enrolment to reach target sample size	12-13
**Methods: Assignment of interventions (for controlled trials)**			
Allocation: sequence generation	#16a	Method of generating the allocation sequence (eg, computer-generated random numbers), and list of any factors for stratification. To reduce predictability of a random sequence, details of any planned restriction (eg, blocking) should be provided in a separate document that is unavailable to those who enrol participants or assign interventions	13

For peer review only

Allocation concealment mechanism	#16b	Mechanism of implementing the allocation sequence (eg, central telephone; sequentially numbered, opaque, sealed envelopes), describing any steps to conceal the sequence until interventions are assigned	13
Allocation: implementation	#16c	Who will generate the allocation sequence, who will enrol participants, and who will assign participants to interventions	13
Blinding (masking)	#17a	Who will be blinded after assignment to interventions (eg, trial participants, care providers, outcome assessors, data analysts), and how	N/A
Blinding (masking): emergency unblinding	#17b	If blinded, circumstances under which unblinding is permissible, and procedure for revealing a participant's allocated intervention during the trial	N/A
**Methods: Data collection, management, and analysis**			
Data collection plan	#18a	Plans for assessment and collection of outcome, baseline, and other trial data, including any related processes to promote data quality (eg, duplicate measurements, training of assessors) and a description of study instruments (eg, questionnaires, laboratory tests) along with their reliability and validity,	13-14

For peer review only

		if known. Reference to where data collection forms can be found, if not in the protocol	
Data collection plan: retention	#18b	Plans to promote participant retention and complete follow-up, including list of any outcome data to be collected for participants who discontinue or deviate from intervention protocols	13-14
Data management	#19	Plans for data entry, coding, security, and storage, including any related processes to promote data quality (eg, double data entry; range checks for data values). Reference to where details of data management procedures can be found, if not in the protocol	14
Statistics: outcomes	#20a	Statistical methods for analysing primary and secondary outcomes. Reference to where other details of the statistical analysis plan can be found, if not in the protocol	14-15
Statistics: additional analyses	#20b	Methods for any additional analyses (eg, subgroup and adjusted analyses)	14-15
Statistics: analysis population and missing data	#20c	Definition of analysis population relating to protocol non-adherence (eg, as randomised analysis), and any statistical methods to handle missing data (eg, multiple imputation)	14-15

Methods: Monitoring

For peer review only

Data monitoring: formal committee	#21a	Composition of data monitoring committee (DMC); summary of its role and reporting structure; statement of whether it is independent from the sponsor and competing interests; and reference to where further details about its charter can be found, if not in the protocol. Alternatively, an explanation of why a DMC is not needed	15-16
Data monitoring: interim analysis	#21b	Description of any interim analyses and stopping guidelines, including who will have access to these interim results and make the final decision to terminate the trial	15-16
Harms	#22	Plans for collecting, assessing, reporting, and managing solicited and spontaneously reported adverse events and other unintended effects of trial interventions or trial conduct	16
Auditing	#23	Frequency and procedures for auditing trial conduct, if any, and whether the process will be independent from investigators and the sponsor	15-16
**Ethics and dissemination**			
Research ethics approval	#24	Plans for seeking research ethics committee / institutional review board (REC / IRB) approval	16-17

For peer review only

Protocol amendments	#25	Plans for communicating important protocol modifications (eg, changes to eligibility criteria, outcomes, analyses) to relevant parties (eg, investigators, REC / IRBs, trial participants, trial registries, journals, regulators)	16-17
Consent or assent	#26a	Who will obtain informed consent or assent from potential trial participants or authorised surrogates, and how (see Item 32)	12-13
Consent or assent: ancillary studies	#26b	Additional consent provisions for collection and use of participant data and biological specimens in ancillary studies, if applicable	N/A
Confidentiality	#27	How personal information about potential and enrolled participants will be collected, shared, and maintained in order to protect confidentiality before, during, and after the trial	17
Declaration of interests	#28	Financial and other competing interests for principal investigators for the overall trial and each study site	21-22
Data access	#29	Statement of who will have access to the final trial dataset, and disclosure of contractual agreements that limit such access for investigators	22
Ancillary and post trial care	#30	Provisions, if any, for ancillary and post-trial care, and for compensation to those who suffer harm from trial participation	N/A

For peer review only

Dissemination policy: trial results	#31a	Plans for investigators and sponsor to communicate trial results to participants, healthcare professionals, the public, and other relevant groups (eg, via publication, reporting in results databases, or other data sharing arrangements), including any publication restrictions	16-17
Dissemination policy: authorship	#31b	Authorship eligibility guidelines and any intended use of professional writers	22
Dissemination policy: reproducible research	#31c	Plans, if any, for granting public access to the full protocol, participant-level dataset, and statistical code	N/A
**Appendices**			
Informed consent materials	#32	Model consent form and other related documentation given to participants and authorised surrogates	APPENDIX 1
Biological specimens	#33	Plans for collection, laboratory evaluation, and storage of biological specimens for genetic or molecular analysis in the current trial and for future use in ancillary studies, if applicable	N/A

None The SPIRIT checklist is distributed under the terms of the Creative Commons Attribution License CC-BY-ND 3.0. This checklist can be completed online using https://www.goodreports.org/, a tool made by the EQUATOR Network in collaboration with Penelope.ai

For peer review only

BMJ Open

Usual care and a self-management support programme vs usual care and a relaxation programme for people living chronic headache disorders: a randomised controlled trial protocol (CHESS)

Journal:	*BMJ Open*
Manuscript ID	bmjopen-2019-033520.R1
Article Type:	Protocol
Date Submitted by the Author:	28-Nov-2019
Complete List of Authors:	Patel, Shilpa; University of Warwick, Warwick Medical School, Clinical Trials Unit<br>Carnes, Dawn; Queen Mary University of London, Centre for Primary Care and Public Health<br>Eldridge, Sandra; Queen Mary University of London, Centre for Primary Care and Public Health<br>Ellard, David; University of Warwick, Warwick Medical School, Clinical Trials Unit<br>Griffiths, Frances; University of Warwick, Warwick Medical School, Division of Health Sciences<br>Haywood, Kirstie; University of Warwick, Warwick Medical School, Division of Health Sciences<br>Hee, Siew Wan; University of Warwick, Warwick Medical School<br>Mistry, Dipesh; University of Warwick, Warwick Medical School, Clinical Trials Unit<br>Mistry, Hema; University of Warwick, Warwick Medical School, Warwick Evidence<br>Nichols, Vivien; University of Warwick, Warwick Medical School, Clinical Trials Unit<br>Petrou, Stavros; University of Warwick, Warwick Medical School<br>Pincus, Tamar; Royal Holloway University of London, Psychology<br>Potter, Rachel; University of Warwick, Warwick Medical School, Clinical Trials Unit<br>Sandhu, Harbinder; University of Warwick Warwick Medical School, Clinical Trials Unit<br>Taylor, Stephanie; Queen Mary University of London, Centre for Primary Care and Public Health<br>Underwood, Martin; University of Warwick, Warwick Medical School, Clinical Trials Unit<br>White, Kimberley; University of Warwick, Warwick Medical School, Clinical Trials Unit<br>Matharu, Manjit; University College London Hospitals NHS Foundation Trust National Hospital for Neurology and Neurosurgery
**Primary Subject Heading**:	Neurology
Secondary Subject Heading:	General practice / Family practice, Health services research

Keywords:	chronic headache, tension type headache, Migraine < NEUROLOGY, self-management, PRIMARY CARE, study protocol

SCHOLARONE™
Manuscripts

TITLE: Usual care and a self-management support programme vs usual care and a relaxation programme for people living chronic headache disorders: a randomised controlled trial protocol (CHESS)

AUTHORS:
Shilpa Patel, Warwick Clinical Trials Unit, Warwick Medical School, University of Warwick, Coventry. CV4 7AL, UK Shilpa.Patel@warwick.ac.uk

Dawn Carnes, Centre for Primary Care and Public Health, Blizard Institute Barts and The London School of Medicine and Dentistry, Queen Mary University of London, London, UK d.carnes@qmul.ac.uk

Sandra Eldridge, Centre for Primary Care and Public Health, Blizard Institute Barts and The London School of Medicine and Dentistry, Queen Mary University of London, London, UK s.eldridge@qmul.ac.uk

David Ellard, Warwick Clinical Trials Unit, Warwick Medical School, University of Warwick, Coventry, CV4 7AL, UK D.R.Ellard@warwick.ac.uk

Frances Griffiths, Health Sciences, Warwick Medical School, University of Warwick, CV4 7AL, UK f.e.griffiths@warwick.ac.uk

Kirstie Haywood, Division of Health Sciences, Warwick Medical School, University of Warwick, Coventry, CV4 7A, UK K.L.Haywood@warwick.ac.uk

Siew Wan Hee, Statistics and Epidemiology Unit, Warwick Medical School, University of Warwick, CV4 7AL, UK s.w.hee@warwick.ac.uk

Dipesh Mistry, Warwick Clinical Trials Unit, Warwick Medical School, University of Warwick, Coventry, CV4 7AL, UK D.Mistry@warwick.ac.uk

Hema Mistry, Population Evidence and Technologies, Warwick Medical School, University of Warwick, Coventry, CV4 7AL, UK Hema.Mistry@warwick.ac.uk

Vivien Nichols, Warwick Clinical Trials Unit, Warwick Medical School, University of Warwick, Coventry, CV4 7AL, UK V.P.Nichols@warwick.ac.uk

Stavros Petrou, Warwick Clinical Trials Unit, Warwick Medical School, University of Warwick, Coventry, CV4 7AL, UK S.Petrou@warwick.ac.uk

Tamar Pincus, Royal Holloway, University of London, Department of Psychology, Surrey, TW20 0EX, UK T.Pincus@rhul.ac.uk

Rachel Potter, Warwick Clinical Trials Unit, Warwick Medical School, University of Warwick, Coventry, CV4 7AL, UK r.potter@warwick.ac.uk

Harbinder Sandhu, Warwick Clinical Trials Unit, Warwick Medical School, University of Warwick, Coventry, CV4 7AL, UK Harbinder.K.Sandhu@warwick.ac.uk

For peer review only

Stephanie JC Taylor, Centre for Primary Care and Public Health, Blizard Institute Barts and The London School of Medicine and Dentistry, Queen Mary University of London, London, UK s.j.c.taylor@qmul.ac.uk

Martin Underwood, Warwick Clinical Trials Unit, Warwick Medical School, University of Warwick, Coventry, CV4 7AL, UK M.Underwood@warwick.ac.uk

Kimberley White, Warwick Clinical Trials Unit, Warwick Medical School, University of Warwick, Coventry, CV4 7AL, UK Kimberley.White@warwick.ac.uk

Manjit Matharu, Headache Group, UCL Institute of Neurology and The National Hospital for Neurology and Neurosurgery, Queen Square, London, WC1N 3BG, UK manjit.matharu@nhs.net

On behalf of the CHESS team.

Corresponding author:
Shilpa Patel, Warwick Clinical Trials Unit, Warwick Medical School, University of Warwick, Coventry. CV4 7AL Shilpa.Patel@warwick.ac.uk
Tel: 44 (0) 2476 150 405

For peer review only

ABSTRACT

Introduction: Chronic headaches are poorly diagnosed and managed, and can be exacerbated by medication overuse. There is insufficient evidence on the non-pharmacological approaches to helping people living with chronic headaches.

Methods and analysis: CHESS (Chronic Headache Education and Self-management Study) is a pragmatic randomised controlled trial to test the effectiveness and cost effectiveness of a self-management education support programme on top of usual care for patients with chronic headaches against a control of usual care and relaxation. The intervention is a two day group course based on education, personal reflection and a cognitive behavioural approach, plus a nurse-led one-to-one consultation and follow up over 8 weeks. We aim to recruit 689 participants (356 to the intervention arm and 333 to the control) from primary care and self-referral in London and the Midlands. The trial is powered to show a difference of 2.0 points on the Headache Impact Test (HIT6); a patient reported outcome measure at 12 months post randomisation. Secondary outcomes include health related quality of life, self-efficacy, social activation and engagement, anxiety and depression and health care utilisation. Outcomes are being measured at four, eight and 12 months. Cost-effectiveness will be expressed in terms of incremental cost per quality-adjusted life year gained.

Ethics and dissemination: This trial will provide data on effectiveness and cost-effectiveness of a self-management support programme for chronic headaches. The results will inform commissioning of services and clinical practice. North West - Greater Manchester East Research Ethics Committee have approved the trial. The current protocol version is 3.6 date 7th March 2019.

Trial registration number: ISRCTN79708100. Registered 16th December 2015, http://www.isrctn.com/ISRCTN79708100

Keywords: Chronic headache, tension type headache, migraine, self-management, primary care, study protocol

For peer review only - http://bmjopen.bmj.com/site/about/guidelines.xhtml

ARTICLE SUMMARY

Strengths and limitations of the study

- This trial presents a high quality randomised controlled trial designed to determine the effectiveness and cost-effectiveness of a group self-management support programme for adults with chronic headaches.
- The intervention underpinning the trial is a complex intervention which has been designed using the best available evidence and theory. The full details of the intervention development and design have been peer reviewed and published.
- A separate mixed-methods process evaluation is running alongside the trial and will explore fidelity of the intervention and implementation. A detailed process evaluation protocol has been peer reviewed and published.
- The success of the trial will be dependent on the ability to deliver this complex group intervention, ensure fidelity and subsequently obtain follow-up data in this population.

For peer review only

INTRODUCTION

Headache disorders are a major cause of pain and disability.[1] The definition of a chronic headache is that the person has headache for 15 or more days per month for at least three months (https://www.ichd-3.org/). Chronic headache disorders mainly affect the young adult population, many of whom have both work and family commitments.[2] The most common chronic headache disorders are chronic tension type headache, chronic migraine, and medication overuse headaches (MOH).[1] Tension type headache and migraine are primary headaches. Medication overuse is a secondary headache that can develop in people with frequent acute headaches who take analgesics, or specific anti-migraine compounds (e.g. triptans) for ≥10 days per month.

Around 2-4% of the global population experience chronic headaches; ; that is headache on 15 or more days per month for greater than three months.[3] Between a quarter and half of those affected also have MOH, which has a prevalence of 1%.[4-6] Many people with chronic headache have undiagnosed chronic migraine.[7] More appropriate use of pharmacological treatment has the potential to improve outcomes for people living with chronic headache disorders. This might mean introducing migraine prophylactic drugs or using fewer analgesics/triptans. Depression, anxiety, poor sleep, stress and poor self-efficacy for managing headaches are prognostic markers for a poor outcome in chronic headache disorders.[8] Limited qualitative data indicate that chronic headache disorders can directly and indirectly drive behaviour: that people live with the 'spectre of headache' (an array of concerns people with chronic headaches have to take into account when forward planning) and that headaches lead to strained relationships.[9] For people living with chronic migraine or chronic tension type headache non-pharmacological self-management approaches may improve headache related symptoms, whilst not affecting headache frequency.[10] Group interventions using a cognitive behavioural approach, mindfulness and educational components may be more effective than alternative interventions.[10]

Supportive self-management approaches are well established in the management of several chronic painful disorders, but this is not the case for chronic headache disorders.[11-13] The National Institute for Health and Care Excellence (NICE) guidance in England, for example, only makes one positive recommendation for a non-pharmacological treatment for people living with chronic headaches; to consider a course of acupuncture for people with chronic migraine or chronic tension type headache.[14]

We describe here the protocol for a randomised controlled trial (RCT) to estimate the effectiveness and cost-effectiveness of a group self-management support programme for adults with chronic headaches arising from migraine or tension type headache, with or without medication overuse. The CHESS intervention consists of a group education and self-management support programme plus a tailored one-to-one headache consultation exploring medication optimisation, lifestyle factors and goals. This active arm of the trial is being compared to usual care plus a relaxation programme.

METHODS AND ANALYSIS

The methods section is structured in accordance with the SPIRIT 2013 recommendations.[15] Here we provide an overview of the trial plus a brief description of the proposed health economic evaluation, process evaluation and patient and public involvement.

The primary objective is to estimate the clinical and cost-effectiveness of a group education and self-management programme for people living with chronic headache recruited from primary care when compared to a GP care plus relaxation control group.

For peer review only - http://bmjopen.bmj.com/site/about/guidelines.xhtml

1. Participants, interventions and outcomes

Study setting

Primary care settings in two localities; London and the Midlands.

Eligibility criteria

The population of interest are adults living with chronic migraine or chronic tension type headache, with or without medication overuse. Part of the challenge in interpreting the findings of research in people living with chronic headaches is the often poor reporting of participants' phenotypic characteristics, meaning it is not possible to draw conclusions for specific chronic headache types.[9 16] For this trial, it is important that we are able to define our participants' headache type, or types, accurately and exclude those with non-eligible headache types. We, therefore developed and validated a logic model for use in a telephone interview by a nurse (non-headache expert) that would allow us to identify and classify people meeting our entry criteria, whilst also excluding people with secondary headaches, other than medication overuse and other causes of primary headaches (Table 1).[17]

Table 1: Inclusion and exclusion criteria

Inclusion criteria	Exclusion criteria
1. Able and willing to comply with the study procedures and provide written informed consent<br>2. Aged ≥18 years (no upper limit)<br>3. Living with chronic headache; defined as headache on 15 or more days per month for at least the preceding three months<br>4. The nurse telephone classification interview confirms headache type to be chronic migraine, or chronic tension type headache, with or without medication overuse headache<br>5. Fluent in written and spoken English.	1. Unable to attend the group sessions<br>2. No access to a telephone (for classification interview)<br>3. Has an underlying serious psychological disorder with ongoing symptoms that preclude or significantly interfere with participation in the group intervention<br>4. Previous entry or randomisation in the present trial<br>5. Currently participating in another clinical trial of headache treatments or unregistered medicinal product, or less than 90 days have passed since completing participation in such a trial.

Pregnancy is not an exclusion criterion. However, any pregnant women randomised to the active intervention will be advised to speak to their General Practitioner (GP) with regards to medication and the CHESS intervention nurses will not discuss this with them during the consultation for safety reasons.

Our previous experience is that the challenges of running non-English language group interventions for chronic painful disorders are too great to do successfully within a randomised controlled trial.[18] Furthermore the main outcomes are not validated in those languages other than English which are

For peer review only - http://bmjopen.bmj.com/site/about/guidelines.xhtml

relevant to a UK context. For these reasons we are excluding people who are not fluent in written and spoken English.

In this pragmatic trial our entry criteria reflect the point in the care pathway where our intervention will be offered. Specifically, general practitioners will refer people they identify with chronic headaches into the service. Thus, there is not the pre-randomisation run-in one might expect in a more explanatory drug trial and we base assessment of the presence of chronic headache on a single telephone assessment and include a mixture of headache types.

Interventions

The intervention consists of a group education and self-management programme, an eight-week headache paper diary, a one-to-one nurse-led consultation and follow-up telephone calls, where necessary, for up to eight weeks. We have described its development in detail elsewhere.[19] We summarise it briefly here.

The programme is run over two days in a two week period for around 10 participants per group (target six to twelve participants), is facilitated by two intervention trained health care professionals, at least one of whom is a nurse (the second facilitator could be a nurse or other registered allied health professional such as psychologist, physiotherapist, chiropractor, occupational therapist). We originally planned to run sessions using a nurse, and a lay facilitator living with chronic headaches. However, in our feasibility study we found it difficult to recruit people living with chronic headaches to act as facilitators and of those that were recruited several found it difficult to commit to facilitation of courses because of the unpredictability of their condition. The intervention builds on a previously developed and tested educational and cognitive behavioural self-management intervention for people with chronic musculoskeletal pain.[20]

The aim of the course is to encourage and enable those with chronic headaches to recognise unhelpful thought patterns and behaviours that contribute to their headache burden and to do something about them. The course provides participants with an overall toolbox of strategies that could help in the management of their headaches. These strategies include psychological techniques to change perceptions and feelings about living with chronic headaches as well as more practical strategies around lifestyle factors and medication. The group intervention is delivered using a range of methods including: group discussions, ideas generation, sharing narratives and experiences, problem solving, role play and taster activity sessions. The programme includes a range of behaviour change techniques including; barrier identification, general encouragement, instruction from the group facilitators, provision of feedback, and allowing opportunities for social comparison in the group. We also include an educational video (available on disc and on-line) following feedback from our patient and public involvement (PPI) group on the importance of having something to show family and friends about their 'invisible' disorder.

The sessions take place on weekdays and, where possible, during school hours to accommodate those with child care responsibilities. Sessions are held in easily accessible community venues and GP practices.

Participants in the intervention arm are asked to complete a paper headache diary for a period of up to eight weeks prior to the two-day programme. A one-to-one, individually tailored nurse-led consultation follows the two-day programme. During this session the nurse classifies the participant's headache type, discuss medication and lifestyle factors and explore participants' goals. This discussion is backed up by written information (for patient and GP), consistent with NICE guidance, to support shared informed decision making between the patient and their GP, about medication choices.[21] All participants will be offered telephone follow-up for up to eight weeks. The frequency of these follow-

For peer review only - http://bmjopen.bmj.com/site/about/guidelines.xhtml

up calls will be individually negotiated and agreed with participants during the one-to-one consultation. Full details of the intervention content have been published elsewhere.[19]

Control intervention

Previous studies of this nature have reported that a usual care control arm was not an incentive to join the study.[11] We know from experience that patients enjoy the relaxation part of pain self-management programmes.[13] Therefore as an incentive to participate, the control group receive standard usual care plus a relaxation CD and instructions for use over the duration of the study, up until final follow-up at 12 months. Participants are free to continue using the relaxation CD thereafter should they wish.

As all participants in the trial have a headache classification interview prior to randomisation, we feed back the results of that classification interview to control participants, and their GPs, together with advice on headache management. In this way we ensure all participants receive best usual care.

The relaxation CD or downloadable MPG file (from the CHESS website) consists of a 17-minute relaxation audio script that starts by focusing on the breath before talking the participant through progressive relaxation of muscles. The participants in the control group choose when, where and how often they do the relaxation but are advised to try it two to three times a week, or as often as they feel appropriate over the course of the study.

Facilitator recruitment and training

All group sessions have two facilitators, one of whom must be a registered nurse, the other facilitator is any allied health care professional, registered with a regulatory body, with an interest in this patient population and condition. Facilitators were recruited via advertisements in allied health care profession organisations and trained over a two-day period to deliver the self-management intervention. Nurses received a further day of training to cover the one-to-one consultations. The training course covers the content of the intervention, facilitation skills, managing groups and trial procedures. Those undergoing the training were assessed using a learning assessment form at the end of the training course to check their knowledge and understanding. Those evaluated to be competent were asked to deliver the intervention. Those who either struggled with some elements of the content or expressed any concerns about delivering the intervention were provided with additional one to one support by the trainers.

Intervention quality assessment

Quality assurance of the intervention is important to ensure that the trial intervention is delivered consistently and in accordance with the trial protocol and manuals. As part of the intervention development we have produced two very detailed manuals, the first which covers the two days of group sessions and the second which details the process for the one to one consultations. All facilitators have been appropriately trained on the delivery of the intervention in accordance with the manuals. They have been instructed to use the manuals to guide them through delivery of the intervention.

To quality assure the course delivery we will aim to observe each facilitator. We will observe each facilitator early in their facilitation to allow any difficulties or challenges to be addressed. Subsequently they will be observed mid-way through. Observations will be by session and the number of sessions observed will depend on the ability of the observer to capture the required information.

The observations will be conducted by members of the CHESS team who have knowledge of the intervention and its delivery. Observers will complete an observation form which will address facilitator skills and adherence to content delivery. Feedback will be provided on the day where possible. If this is not possible the observer will arrange to contact the facilitator by phone. The study

For peer review only - http://bmjopen.bmj.com/site/about/guidelines.xhtml

team will discuss any difficulties with the facilitator to minimise impact on the rest of the course and to help with the delivery of future courses. For any facilitators struggling the central research team will monitor their personal reflections for the remaining duration of the course and follow-up with phone calls if required.

Outcome measures - Primary outcome measure; headache related quality of life

Our primary outcomes is headache related quality of life assessed at the primary endpoint, 12 months after randomisation. For sample size determination we have specified the Headache Impact Test (HIT-6), a 6-item patient-reported outcome measure (PROM), as our measure of the primary outcome.[22] The HIT-6 provides a short overall assessment of headache impact – with items assessing fatigue, pain, social functioning, emotional well-being and cognition. Prior to selecting our outcome measures, we did a systematic review of patient reported outcome measures for headaches.[23] Only for the HIT-6 did we find acceptable evidence supporting its use for our target population who may not have been given a headache diagnosis prior to study entry.

Another measure, shortlisted in the review, was the 14-item Migraine-Specific Quality of Life Questionnaire (MSQ v2.1) assesses the role restrictions, limitations and emotional impact of migraine.[23] The MSQv2.1 has acceptable evidence of psychometric properties following completion in a migraine population.[23-26]

To inform our selection of outcome measures we undertook interviews (n=14) with people with chronic headache. Our participants described greater perceived relevance of the MSQ(v2.1) compared to the HIT-6. With permission from GSK, the copyright holders, we modified the MSQ (v2.1), changing the focus from 'migraine' to 'headache'; the modified measure was renamed as the 'Chronic Headache Quality of Life Questionnaire' (CHQLQ v1.0). In our ongoing work we are assessing the performance of this modified instrument. In the event its performance is superior to the HIT-6 we will consider changing the primary outcome.

Both the HIT-6 and the CHQLQ v1.0 will be collected via postal questionnaire at baseline, four, eight and 12 months post randomisation.

Outcome measures - other secondary outcomes measures

The following secondary outcome measures are being collected:

1. *Headache days:* Our main outcome describing headaches days will be headaches days in the preceding month reported at baseline and follow-up postal questionnaires. We will also estimate total headaches days over the whole study period from patient reported data collected via a smartphone app.
2. *Headache impact:* We will assess headache impact based on their typical duration and severity reported in baseline and follow-up questionnaires
3. *Composite headache outcome:* We will produce a composite headache outcome of headache days x severity x duration using data from the smartphone app and questionnaires.
4. *Generic health-related quality of life*: We have included two standard measures of health-related quality of life – the SF-12 V2 and EQ-5D-5L.[27-29] There is limited, but acceptable, evidence supporting the use of the SF-12 V2 to assess overall quality of life in a headache population.[23] There is no such evidence for the EQ-5D-5L.[23] We will therefore, use EQ-5D-5L primarily for our health economic analyses.[28]
5. *Emotional well-being:* Hospital Anxiety and Depression Scale (HADs) - Psychological distress is extremely common in people living with chronic pain. HADs has been used in many previous studies of chronic pain, including the COPERS study where we identified positive effects on both anxiety and depression in a chronic pain population.[18 30]

For peer review only - http://bmjopen.bmj.com/site/about/guidelines.xhtml

6. *Self-Efficacy:* Pain Self-Efficacy Questionnaire (PSEQ) - Self-efficacy is an important mediator for how self-management interventions may improve patient outcomes. It is important, therefore, to measure change in self-efficacy as part of understanding the causal pathway for any change and informing our process evaluation.[31] We have previously reviewed measures of self-efficacy and concluded that PSEQ is the most appropriate choice for studies of this nature; although all current measures have limitations.[32]
7. *Social Activity: Social Integration Subscale of the Health Education Impact Questionnaire (heiQ)* - Chronic headache can result in a disrupted lifestyle and a reduced quality of life both during and between attacks; the impact of chronic headache on an individual's ability to commit to social plans is an important aspect of quality of life. Successful treatment should seek to improve both overall quality of life, as well as an individual's quality of life during the attack, including their ability to integrate in society. A well-developed, condition-specific measure should seek to capture these distinctions. The five-item Social Integration Subscale (SIS) is one of eight domains contained within the heiQ, a measure of the impact of patient education programmes in chronic conditions.[33]
8. *Bodily pain:* Chronic headache commonly co-exists with other chronic painful disorders such as low back pain.[34-37] The CHESS intervention might affect the troublesomeness of other bodily pains. We will collect these data using a previously validated Troublesomeness Grid.[38]

At baseline we also collect data on age, gender, ethnic group, age at leaving full time education and current work status.

We will collect follow-up data at four, eight, and 12 months after randomisation by postal questionnaire survey. A £5 high street voucher is enclosed as a token of our appreciation at each initial time point. We will send out two postal follow-up reminders. In the event that no response is obtained, we will aim to collect our primary clinical outcome data by telephone. This includes the HIT-6 and EQ-5D-5L.

The smartphone data will be collected weekly for six months from initial eligibility and then monthly until 12 months after randomisation. A paper version of the app will be available to those that do not use a smartphone.

If there are missing data (for our key clinical outcomes), this will be followed up with the participant who completed the form, as soon as possible by a member of the team over the phone. We will phone the participant and enter the correct information onto the form, this will be initialled and dated.

Participant timelines

Figure 1 shows the recruitment flow and table 2 shows the study timelines.

Figure 1 – here

Table 2: Schedule of enrolment, interventions, and assessments

**Activity**	**Pre-enrolment to allocation**	**Randomisation to 12 weeks**	**4 months**	**8 months**	**12 months**
Expression of interest	X				

For peer review only - http://bmjopen.bmj.com/site/about/guidelines.xhtml

Trial information	X				
Download app to record headache patterns (weekly for six months and then monthly until the end of follow-up at 12 months)	X	X	X	X	X
Questionnaire Demographic information, HIT-6, CHQLQ, headache days, SF12, EQ-5D-5L, HADs, PSEQ, HeiQ, troublesomeness	X				
Consent	X				
Headache classification interview	X				
Allocation		X			
Intervention plus completion of paper headache diary for duration of up to eight weeks before attending group		X			
Control		X			
HIT-6, CHQLQ, headache days, SF12, EQ-5D-5L, HADs, PSEQ, HeiQ, troublesomeness			X		
2 postal follow up reminders and phone call for PO if needed			X		
HIT-6, CHQLQ, headache days, SF12, EQ-5D-5L, HADs, PSEQ, HeiQ, troublesomeness				X	
2 postal follow up reminders and phone call for PO if needed				X	
HIT-6, CHQLQ, headache days, SF12, EQ-5D-5L, HADs, PSEQ, HeiQ, troublesomeness					X
2 postal follow up reminders and phone call for PO if needed					X

HIT-6 (Headache Impact Test),[22] CHQLQ (Chronic Headache Quality of Life Questionnaire), SF12 (Short Form 12-item Health Survey),[27 29] EQ-5D-5L (EuroQoL),[28] HADs (Hospital Anxiety and Depression Scale),[30] PSEQ (Pain Self-Efficacy Questionnaire),[31] HeiQ (Health Education Impact Questionnaire)[33]

Sample size

For the purposes of our sample size calculation, the primary clinical outcome is the difference in mean HIT-6 score at 12 months post randomisation between the self-management group programme and the usual care relaxation therapy (control arm). The HIT-6 outcome measure is a continuous scale with higher values indicating more severe impact on daily life. From our systematic reviews, we anticipate a worthwhile difference to be 2.0, i.e. mean outcome in the control arm is 2.0 units higher than for the intervention.[23 39] In our feasibility study (N=114), the standard deviation of the HIT-6 score at baseline was 6.87.[40]

Participants are randomised to either the self-management group or usual care and relaxation therapy. In this design, there may be a clustering effect in the self-management group and not in the control arm, which needs to be allowed for in the sample size calculation. Based on similar trials we assume that the intra-class correlation coefficient (ICC) is 0.01.[18] The anticipated average size of the self-management programme is 10.

The minimum sample size required was estimated using Moerbeek and Wong's method to account for grouping in one arm.[41] To detect a between-group difference of 2.0 with standard deviation of 6.9 (a standardised mean difference of 0.29) and assuming that the ratio of the total variance in the self-management group to the relaxation therapy is 1 at the two-sided 5% significance level and at least 90% power, the sample size required is 523 participants (253 in the relaxation group and 270 in the self-management group).

For peer review only - http://bmjopen.bmj.com/site/about/guidelines.xhtml

In the feasibility study, the overwhelming majority of those recruited, approximately 95%, chronic migraine; just 5% had chronic tension type headache. We want to be able to draw definite conclusions on the specific subgroup of chronic migraine. Therefore, we will base our sample size and primary clinical outcome on the population with chronic migraine. Therefore, based on 95% of our sampled population with chronic migraine and accounting for a 20% loss to follow-up as above, the sample size we would require is 689 with 333 to the relaxation arm and 356 to the self-management programme.

In the feasibility study we recruited around 1/1,000 of practice population to take part,[40] we therefore anticipate we need to recruit from around 100 general practices with a combined list size of around 700,000.

In consultation with the Data Monitoring and Ethics Committee (DMEC), we will review the sample size around halfway through recruitment to ensure we have recruited sufficient participants with chronic migraine and revise our estimates using within trial data on the variance of our primary outcome at baseline. This review will be based on the headache classification and actual baseline standard deviation of our sampled population. We might also need to recruit some additional participants to ensure that the final group sessions at each site are adequately populated.

Recruitment

Participants are identified and invited into the study in two ways. Firstly, practices run electronic searches on their databases, to identify people who have consulted with headaches or have been prescribed migraine specific drugs (e.g. triptans, pizotifen) in the preceding two years. Practices then screen the lists for those it would be inappropriate to approach (e.g. poorly controlled serious mental illness, terminal illness, or known secondary causes of headache such as primary or secondary brain tumours), and send approach letters to the remainder.

People can self-refer to the trial. Participating general practices, the principal pharmacies used by their patients, are supplied with a study poster for display in patient areas inviting people to contact the study team if they have headaches and are interested in participating. Information about the trial is made available on the poster (in general practices and pharmacies), and websites (https://warwick.ac.uk/fac/med/research/ctu/trials/other/chess/ and https://warwick.ac.uk/fac/med/research/ctu/trials/chess. People who find about the trial through the internet or following media exposure, and can travel to sessions, can also self-refer to the trial.

Using both approaches will allow people receiving GP treatment for chronic headaches who are not coded in the GP system as having headaches, and those who are self-medicating their headaches, the opportunity to join the study. We anticipate that we will primarily recruit people registered with participating practices; however, we will not restrict recruitment to those registered with participating practices.

In addition to these two main recruitment strategies, a study press release was submitted in January 2018 which was picked up by various media outlets and generated further interest in the study from potential participants.

The study co-ordinating team contact people expressing an interest in the study and check that they are eligible, explain the study, and obtain participant's verbal consent to start completing an electronic headache symptom severity, duration & frequency diary (or paper version where there is no access to the internet). The electronic diary is completed weekly for the first six months and subsequently monthly until the end of follow-up at 12 months. The electronic diary is used to identify any early

For peer review only - http://bmjopen.bmj.com/site/about/guidelines.xhtml

effects of rebound headache in those with medication overuse headache. At this time participants are sent a baseline questionnaire and consent form to return in a freepost envelope.

Following receipt of baseline questionnaire and consent form participants are contacted by phone for a nurse headache classification interview. People whose headache is confirmed to meet our entry criteria are then eligible for randomisation. If the nurse has a concern that the potential participant may have another headache type there is a second interview with a doctor skilled in the management of headache disorders from the National Migraine Centre (http://www.nationalmigrainecentre.org.uk/). This is to ensure no one is inappropriately excluded and that people with a headache type needing more specific treatment are directed towards appropriate treatment. For example, 2/108 people in our feasibility study had cluster headaches.[40] In such cases we write to the GP and the participant with details on the excluded headache type and explain that they no longer fit the inclusion criteria for the CHESS trial.

2. Assignment of interventions

Randomisation

The randomisation is stratified by geographical locality (Midlands and Greater London) and headache type (six possible headache types: chronic tension type headache, probable chronic migraine and definite chronic migraine with or without medication overuse headache) using minimisation. Randomisation takes place using an online application specifically developed for the CHESS Study by the Warwick Clinical Trials Unit (WCTU) programming team.

Randomisation takes place when there are around 20 participants who would be able to attend a local group if randomised to the active intervention. Typically these will come from 4-5 proximate general practices. However, people from other localities who are unable to attend a more local group and people who have self-referred to the study who are willing and able to travel to the sessions will also be included. By randomising in groups around two weeks prior to the start of a group, we are able to minimise any delay between randomisation and start of the intervention and ensure everyone has adequate follow-up from when they might have been exposed to the intervention. For those that are subsequently unable to attend after randomisation to a group, we will offer them another group where possible.

Allocation concealment and protection from bias

Allocation concealment will be maintained by using Warwick CTU's centralised randomisation service. All baseline data will be collected prior to randomisation. Blinding will be impossible for participants, facilitators and the central co-ordinating CHESS team. All data entry and checking is done blind to treatment allocation. The unbalanced randomisation means it is not possible blind the trial statistician to treatment allocation.

Our primary outcome is a participant completed outcome. Inevitably, participants will be aware of their treatment allocation. We will develop and sign off a detailed pre-specified statistical analysis plan before allocation codes are released.

3. Data collection, management and analysis

Data collection methods

Data will be collected using two methods, postal survey questionnaires and a smartphone application (App) for headaches. All participants will be asked to complete a smartphone app about their

For peer review only - http://bmjopen.bmj.com/site/about/guidelines.xhtml

headaches. If they do not have access to a smartphone, or do not wish to use the app, a paper copy will be provided. Participants will initially complete the app weekly for up to six months, to cover any period of withdrawal from medication, then monthly thereafter (still requiring them to reflect over the previous 7 days) until the end of the study at 12 months after randomisation. We are working with Clinvivo Ltd a University of Warwick spin-out company specialising in electronic data collection, to capture data on headache frequency, duration and severity electronically using a smartphone App (Table 3 shows the questions completed by participants and mode of response required). The data from the questions in the electronic diary will be numerical and downloaded into a WCTU database using a randomly generated 256-bit AES (Advanced Encryption Standard) key. At the end of the participants 12 months they will be provided with a summary report of their headache application data.

Reminders are sent to those participants who fail to downloaded the app or have not responded for more than three weeks. Reminders are sent by email or by post if there is no email address given.

A daily detailed paper headache diary is kept only by the intervention arm participants and the data will not be analysed by the study team but kept as a record by the participants and used during the one to one consultation where it will be collected and kept by the research team.

Table 3. Questions completed on the smartphone app.

Question	Mode of response
On how many of the last 7 days have you had a headache?	Insert number of headache days
On those days you had a headache, on average how long did they last?	Insert number of hours
On those days you had a headache on average how severe were they?	Scale of 0 to 10 (with 0 being no pain and 10 extremely severe pain)

Data management

All questionnaire data received by the trial team are reviewed for completeness and entered onto a secure, backed-up bespoke database held at WCTU which will be accessible only by authorised members of the team. Data from the smartphone App are downloaded securely (as described above) into the trial database. All data is checked when received, any queries relating to the HIT6 CHQLQ or EQ5D are raised with the participant by telephone.

Statistical analysis of effectiveness and harms

Our primary endpoint analyses will be based on the twelve month data.

Participants' characteristics and reported outcomes will be summarised as means and standard deviations (for continuous data) or frequencies and percentages (for categorical data) by treatment arms. Difference between baseline and the three follow-up time points (four-, eight- and 12-month post randomisation) will be computed for the primary and secondary outcomes by treatment arms.

The primary analysis approach will be intention to treat i.e. the data will be analysed according to the treatment the participant was originally allocated to, irrespective of what they actually received. We will explore the possibility of carrying out a complier averaged causal effect (CACE) analysis as a sensitivity analysis. Our primary analysis will be the difference between the self-management therapy

For peer review only - http://bmjopen.bmj.com/site/about/guidelines.xhtml

(intervention) and the relaxation therapy (control) groups with a 95% confidence interval (CI) in the population with chronic migraine – if the proportion of participants with chronic tension type headache is ≤ 15%.The hypothesis testing of the primary outcome will be two-sided at the 5% level and the main analysis will estimate the treatment effect using a multilevel model to account for clustering (the model used to design this main trial). We will also present results for the whole population (all headache types). If the proportion of chronic tension type headache is >15% then the primary analysis will be according to the whole population of chronic headache (chronic migraine and tension type headache). Our experience is that NICE, was specifically interested in data on specific headache types; excluding data that reported mixed population of people with chronic headaches. We will, therefore in addition to our primary analyses present the results (mean difference and 95% CI) for each of the two headache types with or without medication overuse headache separately, and present results for those with or without medication overuse separately to facilitate future meta-analyses and inform future condition specific guidelines. All analyses will be adjusted for the randomisation stratification factors (types of headache and geographical locality), sex, age and the baseline value of the dependent variable.

Similar analyses will be performed for all the other secondary outcomes. Pre-specified subgroup analyses using formal statistical tests for interaction will examine whether baseline anxiety, depression and severity are moderators of treatment effect.[42] We will assess the level of missingness in the primary outcome and if required, we will use appropriate multiple imputation techniques to impute data and estimate the treatment effect as a form of sensitivity analysis. A full statistical analysis plan, including all primary and secondary analyses, will be written and signed off prior to releasing allocation codes to analysts and others involved in developing the plan.

Using the data from the smartphone App we will generate a composite score for headache impact over the one-year of follow up as the function of headaches days x average duration x average severity. Presenting these data graphically will allow any early benefits or harms from the intervention to be identified.

Health Economic Evaluation

Our economic evaluation will be conducted alongside the trial and we will initially adopt a one-year time horizon from both an NHS and personal social services perspective and, separately for the purpose of a sensitivity analysis a broader societal perspective, to estimate the cost-utility of the intervention. Resource use data will be collected to explore the costs of the delivery of the intervention and to estimate the key cost drivers. This will mainly consist of visits to the GP practice, medication usage and any adverse events or length of stay in the hospital. In terms of costs to society, we will estimate time off work and any productivity losses associated with chronic headaches. Resource use information will be collected using self-completed postal questionnaires completed at four, eight and 12 months after randomisation, as well as the use of routine health service data collected from general practice records. Resource input will be valued using national estimates of unit costs such as the Prescription Cost Analysis database or the Unit Costs of Health and Social Care compendium.[43] Preference-based health-related quality of life outcomes will primarily be assessed through the completion of the EQ-5D-5L at each follow-up point.[44] Quality-adjusted life-years (QALYs) will be calculated as the area under the baseline-adjusted utility curve, and will be calculated using linear interpolation between baseline and follow-up utility scores.

The results of the economic evaluation will be presented using incremental cost-effectiveness ratios, expressed in terms of incremental cost per QALY gained, and cost-effectiveness acceptability curves generated via non-parametric bootstrapping.

For peer review only - http://bmjopen.bmj.com/site/about/guidelines.xhtml

More extensive economic modelling using decision-analytic methods will extend the target population, the time horizon to five years as the long-term natural history is unclear and the decision context, drawing on best available information from the literature together with stakeholder consultations to supplement the trial data. Longer-term costs and consequences will be discounted to present values using nationally recommended discount rates recommended for health technology appraisal. We will use probabilistic sensitivity analysis to estimate the impact of uncertainty over model parameters. We will also use simple sensitivity analysis to assess the robustness of the results to changes in deterministic parameters such as medication dosages, costs, discount rate and time horizon for patients presenting with chronic headaches. We will also explore cost-effectiveness of the intervention by conducting subgroup analyses for the different headache types.

4. Monitoring, ethics and dissemination

Programme steering committee (PSC)

We have established a PSC to oversee the whole programme of work. The PSC functions specifically as a trial steering committee specifically for this trial. The PSC consists of seven members including two patient representative. The PSC will be responsible for oversight and monitoring and will consider any information from surveillance activity of other research in the field.

Data monitoring and Ethics Committee (DMEC)

A data monitoring and ethics committee has been convened consisting of an independent statistician, a trialist, a clinician and a lay member. Confidential reports that summarise the trial data and safety data will be reviewed by the DMC. The DMC will advise the PSC as to whether to continue, amend or terminate the trial based primarily on safety and efficacy considerations

Harms / Adverse Events (AE)

An adverse event or serious adverse event is any event that takes place on the way to, during, or on the way home from the intervention course. This includes the two days of the group course and the one to one nurse appointment. Our experience across multiple studies of group interventions is that adverse events that are directly attributable to the intervention are rare.

Events during the session, such as severe psychological disturbance, any mild or moderate levels of emotional distress as a result of discussing experiences of living with chronic headache, a fall during travel to and from the venue or any other adverse events, occurring during the delivery of the intervention will be reported to the trial co-coordinator by the intervention team. Any short-term increase in headaches as a consequence of medication withdrawal will be captured using the smartphone app (or paper version for those without access to a smartphone).

All adverse events will be managed in line with Warwick CTU's standard operating procedures.

Process Evaluation

A separate mixed-methods process evaluation is running alongside the trial. This will explore both the process of implementation the trial and the process of delivering and receiving the intervention. The process evaluation protocol is reported elsewhere.[45]

The fidelity of the delivery of the intervention will be assessed by this team in three ways, through: audio recording of the groups, facilitator reflection and participant feedback. This will assess facilitator competence, adherence to the course manual and trial procedures and participant interaction and engagement.

Patient and Public Involvement (PPI)

For peer review only - http://bmjopen.bmj.com/site/about/guidelines.xhtml

We have had substantial patient and public involvement in the feasibility study prior to finalising this protocol. Lay members were involved in the development of the classification interview, development of the intervention, PROM selection, and conduct and management of the study via the independent programme steering and trial management group.

Our trial management group includes our lay co-applicants who are representatives of three leading UK migraine charities (The Migraine Trust, Migraine Action (before their merger with Migraine Trust), and National Migraine Centre).

We have developed a lay steering group who are and will be collaboratively involved during the study. At key points in the programme we will approach the lay steering group for input, this will include at the end of the trial when results are disseminated.

ETHICS AND DISSEMINATION

The University of Warwick (Research Impact Services, University of Warwick, Coventry CV4 7AL) are the Sponsor for the study.

Ethics approval was provided by: North West - Greater Manchester East Research Ethics Committee (REC REF: 16/NW/0890) The trial is conducted in accordance with the principles outlined in the Declaration of Helsinki and conforms to GCP and SOPs set-out by the WCTU. As reported above we have convened a PSC and DMC to oversee the trial.

All identifiable data is anonymised and treated as confidential. Participants are informed that they are free to withdraw at any time during any phase of the work.

The findings from this trial will be disseminated via a final report to the NIHR, presentations at conferences and publications in high quality peer-reviewed journals.

For the healthcare professionals involved in the study we will disseminate results of the study through the study website. We will also host a meeting to present the trial results to commissioners and clinicians. For the participants, we will provide a written lay summary of the findings and also publish these on a study specific website; with contact information should they wish to discuss the findings. Our charity partners will be involved with feedback to the organisations they represent.

CONCLUSION

At the time of writing (08/08/2019) a total of 736 participants have been randomised (380 to the intervention arm and 356 to the control arm) and the trial is now in the follow-up phase.
A comprehensive programme of preparatory work has allowed this substantial trial of an education and self-management intervention for people living with chronic headaches to be developed. There is a clear interest in taking part in the trial. Our follow up will be complete in early 2020 and the final results published in 2021.

For peer review only - http://bmjopen.bmj.com/site/about/guidelines.xhtml

REFERENCES

1. Global, regional, and national burden of neurological disorders during 1990-2015: a systematic analysis for the Global Burden of Disease Study 2015. The Lancet. Neurology 2017;**16**(11):877-97.
2. Steiner TJ, Scher AI, Stewart WF, Kolodner K, Liberman J, Lipton RB. The prevalence and disability burden of adult migraine in England and their relationships to age, gender and ethnicity. Cephalalgia : an international journal of headache 2003;**23**(7):519-27.
3. Stovner L, Hagen K, Jensen R, et al. The global burden of headache: a documentation of headache prevalence and disability worldwide. Cephalalgia : an international journal of headache 2007;**27**(3):193-210.
4. Castillo J, Munoz P, Guitera V, Pascual J. Epidemiology of chronic daily headache in the general population. Headache 1999;**39**(3):190-6.
5. Lu SR, Fuh JL, Chen WT, Juang KD, Wang SJ. Chronic daily headache in Taipei, Taiwan: prevalence, follow-up and outcome predictors. Cephalalgia : an international journal of headache 2001;**21**(10):980-6.
6. Wang SJ, Fuh JL, Lu SR, et al. Chronic daily headache in Chinese elderly: prevalence, risk factors, and biannual follow-up. Neurology 2000;**54**(2):314-9.
7. Mehuys E, Paemeleire K, Van Hees T, et al. Self-medication of regular headache: a community pharmacy-based survey. European journal of neurology 2012;**19**(8):1093-9.
8. Probyn K, Bowers H, Caldwell F, et al. Prognostic factors for chronic headache: A systematic review. Neurology 2017;**89**(3):291-301.
9. Nichols VP, Ellard DR, Griffiths FE, Kamal A, Underwood M, Taylor SJC. The lived experience of chronic headache: a systematic review and synthesis of the qualitative literature. BMJ open 2017;**7**(12):e019929.
10. Probyn K, Bowers H, Mistry D, et al. Non-pharmacological self-management for people living with migraine or tension-type headache: a systematic review including analysis of intervention components. BMJ open 2017;**7**(8):e016670.
11. Carnes D, Homer K, Underwood M, Pincus T, Rahman A, Taylor SJ. Pain management for chronic musculoskeletal conditions: the development of an evidence-based and theory-informed pain self-management course. BMJ open 2013;**3**(11):e003534.
12. Pinnock H, Parke HL, Panagioti M, et al. Systematic meta-review of supported self-management for asthma: a healthcare perspective. BMC medicine 2017;**15**(1):64.
13. Taylor SJC, Pinnock H, Epiphaniou E, et al. Health Services and Delivery Research. A rapid synthesis of the evidence on interventions supporting self-management for people with long-term conditions: PRISMS - Practical systematic Review of Self-Management Support for long-term conditions. Southampton (UK): NIHR Journals Library, 2014.
14. Carville S, Padhi S, Reason T, Underwood M. Diagnosis and management of headaches in young people and adults: summary of NICE guidance. BMJ (Clinical research ed.) 2012;**345**:e5765.
15. Chan AW, Tetzlaff JM, Altman DG, et al. SPIRIT 2013 statement: defining standard protocol items for clinical trials. Annals of internal medicine 2013;**158**(3):200-7.
16. Louter MA, Ward TN, Terwindt GM. Chronic headache: What's in a name? Neurology 2017;**89**(3):224-25.
17. Potter R, Hee SW, Griffiths F, et al. Development and validation of a telephone classification interview for common chronic headache disorders. J Headache Pain 2019;**20**(1):2.
18. Taylor SJC, Carnes D, Homer K, et al. Programme Grants for Applied Research. Improving the self-management of chronic pain: COping with persistent Pain, Effectiveness Research in Self-management (COPERS). Southampton (UK): NIHR Journals Library, 2016.
19. Patel S, Potter R, Matharu M, et al. Development of an education and self-management intervention for chronic headache - CHESS trial (Chronic Headache Education and Self-management Study). J Headache Pain 2019;**20**(1):28.

For peer review only - http://bmjopen.bmj.com/site/about/guidelines.xhtml

20. Taylor SJ, Carnes D, Homer K, et al. Novel Three-Day, Community-Based, Nonpharmacological Group Intervention for Chronic Musculoskeletal Pain (COPERS): A Randomised Clinical Trial. PLoS medicine 2016;**13**(6):e1002040.
21. NICE. Headaches in over 12s: diagnosis and management - Clinical guideline [CG150], 2012.
22. Kosinski M, Bayliss MS, Bjorner JB, et al. A six-item short-form survey for measuring headache impact: the HIT-6. Quality of life research : an international journal of quality of life aspects of treatment, care and rehabilitation 2003;**12**(8):963-74.
23. Haywood KL, Mars TS, Potter R, Patel S, Matharu M, Underwood M. Assessing the impact of headaches and the outcomes of treatment: A systematic review of patient-reported outcome measures (PROMs). Cephalalgia : an international journal of headache 2018;**38**(7):1374-86.
24. Jhingran P, Osterhaus JT, Miller DW, Lee JT, Kirchdoerfer L. Development and validation of the Migraine-Specific Quality of Life Questionnaire. Headache 1998;**38**(4):295-302.
25. Martin BC, Pathak DS, Sharfman MI, et al. Validity and reliability of the migraine-specific quality of life questionnaire (MSQ Version 2.1). Headache 2000;**40**(3):204-15.
26. McKenna SP, Doward LC, Davey KM. The Development and Psychometric Properties of the MSQOL: A Migraine-Specific Quality-of-Life Instrument. Clinical drug investigation 1998;**15**(5):413-23.
27. Gandek B, Ware JE, Aaronson NK, et al. Cross-validation of item selection and scoring for the SF-12 Health Survey in nine countries: results from the IQOLA Project. International Quality of Life Assessment. Journal of clinical epidemiology 1998;**51**(11):1171-8.
28. Herdman M, Gudex C, Lloyd A, et al. Development and preliminary testing of the new five-level version of EQ-5D (EQ-5D-5L). Quality of life research : an international journal of quality of life aspects of treatment, care and rehabilitation 2011;**20**(10):1727-36.
29. Jenkinson C, Layte R, Lawrence K. Development and testing of the Medical Outcomes Study 36-Item Short Form Health Survey summary scale scores in the United Kingdom. Results from a large-scale survey and a clinical trial. Medical care 1997;**35**(4):410-6.
30. Zigmond AS, Snaith RP. The hospital anxiety and depression scale. Acta psychiatrica Scandinavica 1983;**67**(6):361-70.
31. Nicholas MK. The pain self-efficacy questionnaire: Taking pain into account. European journal of pain (London, England) 2007;**11**(2):153-63.
32. Miles CL, Pincus T, Carnes D, Taylor SJ, Underwood M. Measuring pain self-efficacy. The Clinical journal of pain 2011;**27**(5):461-70.
33. Osborne RH, Elsworth GR, Whitfield K. The Health Education Impact Questionnaire (heiQ): an outcomes and evaluation measure for patient education and self-management interventions for people with chronic conditions. Patient education and counseling 2007;**66**(2):192-201.
34. Hagen K, Linde M, Steiner TJ, Zwart JA, Stovner LJ. The bidirectional relationship between headache and chronic musculoskeletal complaints: an 11-year follow-up in the Nord-Trondelag Health Study (HUNT). European journal of neurology 2012;**19**(11):1447-54.
35. Jimenez-Sanchez S, Fernandez-de-Las-Penas C, Carrasco-Garrido P, et al. Prevalence of chronic head, neck and low back pain and associated factors in women residing in the Autonomous Region of Madrid (Spain). Gaceta sanitaria 2012;**26**(6):534-40.
36. Plesh O, Adams SH, Gansky SA. Self-reported comorbid pains in severe headaches or migraines in a US national sample. Headache 2012;**52**(6):946-56.
37. Yoon MS, Manack A, Schramm S, et al. Chronic migraine and chronic tension-type headache are associated with concomitant low back pain: results of the German Headache Consortium study. Pain 2013;**154**(3):484-92.
38. Parsons S, Carnes D, Pincus T, et al. Measuring troublesomeness of chronic pain by location. BMC musculoskeletal disorders 2006;**7**:34.
39. Cousins S, Ridsdale L, Goldstein LH, Noble AJ, Moorey S, Seed P. A pilot study of cognitive behavioural therapy and relaxation for migraine headache: a randomised controlled trial. Journal of neurology 2015;**262**(12):2764-72.

For peer review only - http://bmjopen.bmj.com/site/about/guidelines.xhtml

40. White K, Potter R, Patel S, et al. Chronic Headache Education and Self-management Study (CHESS) - a mixed method feasibility study to inform the design of a randomised controlled trial. Submitted. 2019;**19**(30).
41. Moerbeek M, Wong WK. Sample size formulae for trials comparing group and individual treatments in a multilevel model. Statistics in medicine 2008;**27**(15):2850-64.
42. Brookes ST, Whitely E, Egger M, Smith GD, Mulheran PA, Peters TJ. Subgroup analyses in randomized trials: risks of subgroup-specific analyses; power and sample size for the interaction test. Journal of clinical epidemiology 2004;**57**(3):229-36.
43. Authority NBS. Prescription Cost Analysis (PCA) data. Secondary Prescription Cost Analysis (PCA) data. https://www.nhsbsa.nhs.uk/prescription-data/dispensing-data/prescription-cost-analysis-pca-data.
44. van Hout B, Janssen MF, Feng YS, et al. Interim scoring for the EQ-5D-5L: mapping the EQ-5D-5L to EQ-5D-3L value sets. Value in health : the journal of the International Society for Pharmacoeconomics and Outcomes Research 2012;**15**(5):708-15.
45. Nichols VP, Ellard DR, Griffiths FE, Underwood M, Taylor SJC, Patel S. The CHESS trial: protocol for the process evaluation of a randomised trial of an education and self-management intervention for people with chronic headache. Trials 2019;**20**(1):323.

Figure legend

Figure 1 - Shows the recruitment flow chart

For peer review only

ABBREVIATIONS

Abbreviation	Full form
CHESS	Chronic Headache Education and Self-management Study
HIT6	Headache Impact Test
MOH	Medication Overuse Headaches
NICE	National Institute for Health and Care Excellence
RCT	Randomised Controlled Trial
GP	General Practitioner
PPI	Patient and Public Involvement
PROMs	Patient-Reported Outcome Measures
MSQ	Migraine-Specific Quality of Life Questionnaire
CHQLQ	Chronic Headache Quality of Life Questionnaire
SF12	Short Form 12-item Health Survey
EQ-5D-5L	EuroQoL
HADs	Hospital Anxiety and Depression Scale
PSEQ	Pain Self-Efficacy Questionnaire
HeiQ	Health Education Impact Questionnaire
ICC	intra-class correlation coefficient
DMEC	Data Monitoring and Ethics Committee
WCTU	Warwick Clinical Trials Unit
App	Application
CACE	Complier Averaged Causal Effect
CI	Confidence Interval
QALYs	Quality-Adjusted Life-Years
PSC	Programme Steering Committee
AE	Adverse Event
DCM	Definite Chronic Migraine
PCM	Probable Chronic Migraine
TTH	Tension Type Headache

DECLARATIONS

Ethics approval and consent to participate

Ethics approval was given on the 17th Of February 2017 by North West - Greater Manchester East Research Ethics Committee (REC REF: 16/NW/0890). Written consent was taken for participation.

Competing interests statements

MU is a director and shareholder of Clinvivo Ltd. Use of this company was specified in original application for funding to NIHR. MU has recused himself from all discussion regarding the use of the App in this study. All contracting processes have been in accord with University of Warwick financial regulations.

MU was Chair of the NICE accreditation advisory committee until March 2017 for which he received a fee. He is chief investigator or co-investigator on multiple previous and current research grants from the UK National Institute for Health Research, Arthritis Research UK and is a co-investigator on grants funded by Arthritis Australia and Australian NHMRC. He has received travel expenses for speaking at conferences from the professional organisations hosting the conferences He is part of an academic partnership with Serco Ltd related to return to work initiatives. He is an editor of the NIHR journal

For peer review only - http://bmjopen.bmj.com/site/about/guidelines.xhtml

series, and a member of the NIHR Journal Editors group for which he receives a fee. He has published multiple papers on chronic pain some of which are referenced in this paper.

MSM serves on the advisory board for Allergan, Medtronic, Novartis and TEVA and has received payment for the development of educational presentations from Allergan, electroCore, Medtronic, Novartis and TEVA.

SP and HS are directors of Health Psychology Services Ltd which in part provides psychological treatments for those with chronic pain.

Funding statement
This research was funded by the NIHR Programme Grants for Applied Research programme (RP-PG-1212-20018). The views expressed in this publication are those of the author(s) and not necessarily those of the NHS, the NIHR or the Department of Health.

Data access
The final trial dataset will be available for sharing with other research teams after publication of the main trial paper. All data will be anonymised and provided after assessment of any further research proposals and evidence of ethical approvals.

Authors contribution
SP - study concept and design, supported writing of the first draft of the manuscript and finalised the manuscript for submission.
DC – study concept and design, supported writing of the first draft of the manuscript, provided critical revisions to the manuscript
SE - study concept and design, provided critical revisions to the manuscript
DE - study concept and design, provided critical revisions to the manuscript
FG - study concept and design, provided critical revisions to the manuscript
KH - study concept and design, provided critical revisions to the manuscript
SWH - study concept and design, provided critical revisions to the manuscript
DM - study concept and design, provided critical revisions to the manuscript
HM - study concept and design, provided critical revisions to the manuscript
VN - study concept and design, provided critical revisions to the manuscript
SPe - study concept and design, provided critical revisions to the manuscript
TP - study concept and design, provided critical revisions to the manuscript
RP - study concept and design, provided critical revisions to the manuscript
HS - study concept and design, provided critical revisions to the manuscript
SJT - study concept and design, provided critical revisions to the manuscript
MU – conceived the original study design and supported writing of the first draft of the manuscript
KW - study concept and design, provided critical revisions to the manuscript
MSM - study concept and design, provided critical revisions to the manuscript

DC, SE, DE, FG, KH, SWH, HM, SPe, TP, HS, SJT, MU, MSM - original grant holders

All authors have read and approved the final manuscript.

Acknowledgements
The manuscript was written on behalf of the CHESS co-applicants, study team and PPI representatives.

For peer review only - http://bmjopen.bmj.com/site/about/guidelines.xhtml

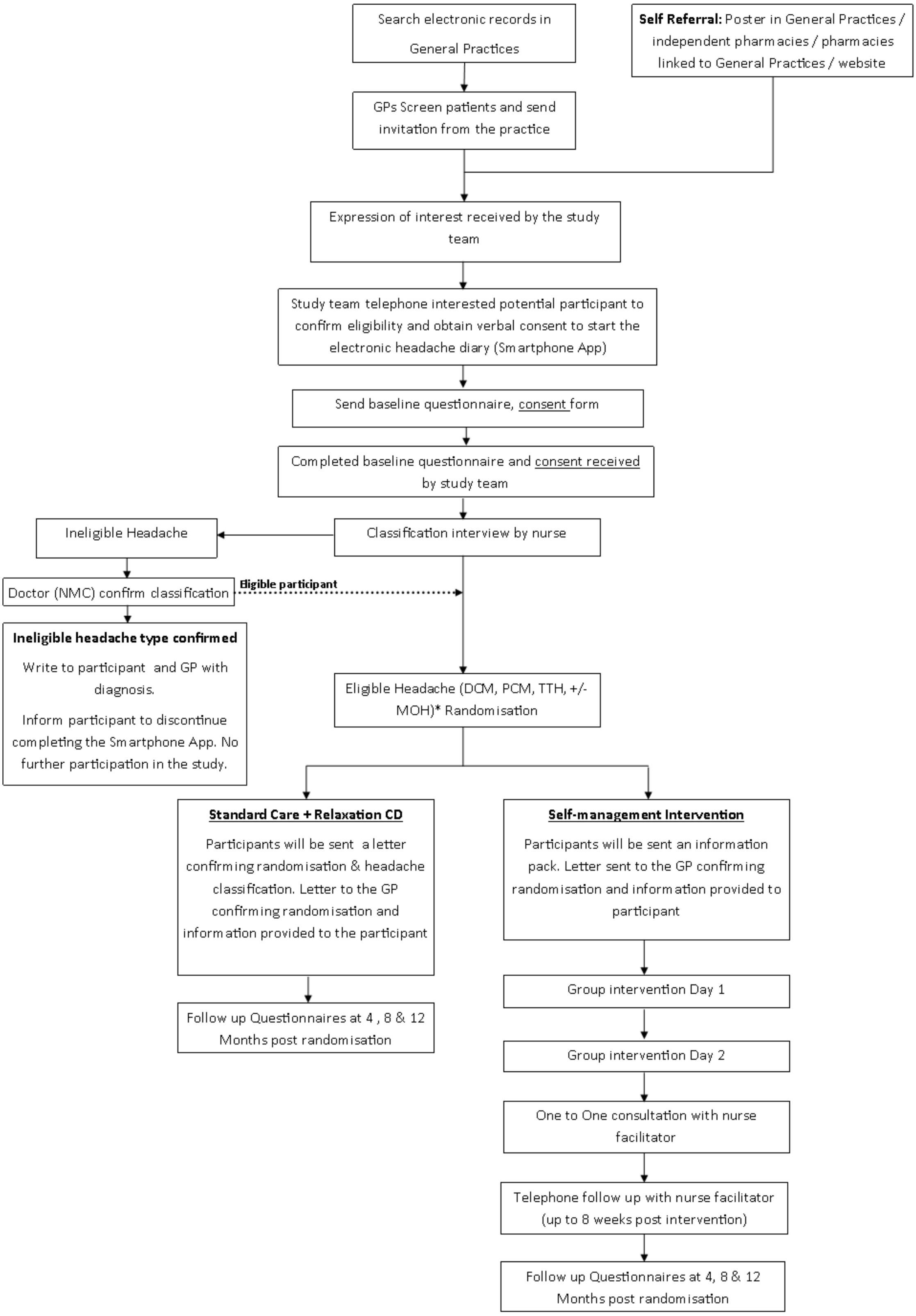

*DMC—Definite Chronic Migraine; PCM— Probable Chronic Migraine; TTH— Tension Type Headache; MOH— Medication Overuse Headache

Reporting checklist for protocol of a clinical trial.

Based on the SPIRIT guidelines.

Instructions to authors

Complete this checklist by entering the page numbers from your manuscript where readers will find each of the items listed below.

Your article may not currently address all the items on the checklist. Please modify your text to include the missing information. If you are certain that an item does not apply, please write "n/a" and provide a short explanation.

Upload your completed checklist as an extra file when you submit to a journal.

In your methods section, say that you used the SPIRITreporting guidelines, and cite them as:

Chan A-W, Tetzlaff JM, Altman DG, Laupacis A, Gøtzsche PC, Krleža-Jerić K, Hróbjartsson A, Mann H, Dickersin K, Berlin J, Doré C, Parulekar W, Summerskill W, Groves T, Schulz K, Sox H, Rockhold FW, Rennie D, Moher D. SPIRIT 2013 Statement: Defining standard protocol items for clinical trials. Ann Intern Med. 2013;158(3):200-207

		Reporting Item	Page Number
**Administrative information**			
Title	#1	Descriptive title identifying the study design, population, interventions, and, if applicable, trial acronym	1

For peer review only

Trial registration	#2a	Trial identifier and registry name. If not yet registered, name of intended registry	3
Trial registration: data set	#2b	All items from the World Health Organization Trial Registration Data Set	N/A
Protocol version	#3	Date and version identifier	3
Funding	#4	Sources and types of financial, material, and other support	22
Roles and responsibilities: contributorship	#5a	Names, affiliations, and roles of protocol contributors	1 & 2
Roles and responsibilities: sponsor contact information	#5b	Name and contact information for the trial sponsor	17
Roles and responsibilities: sponsor and funder	#5c	Role of study sponsor and funders, if any, in study design; collection, management, analysis, and interpretation of data; writing of the report; and the decision to submit the report for publication, including whether they will have ultimate authority over any of these activities	N/A
Roles and responsibilities: committees	#5d	Composition, roles, and responsibilities of the coordinating centre, steering committee, endpoint adjudication committee, data management team, and	15-17

For peer review only

		other individuals or groups overseeing the trial, if applicable (see Item 21a for data monitoring committee)	
**Introduction**			
Background and rationale	#6a	Description of research question and justification for undertaking the trial, including summary of relevant studies (published and unpublished) examining benefits and harms for each intervention	5
Background and rationale: choice of comparators	#6b	Explanation for choice of comparators	5
Objectives	#7	Specific objectives or hypotheses	5
Trial design	#8	Description of trial design including type of trial (eg, parallel group, crossover, factorial, single group), allocation ratio, and framework (eg, superiority, equivalence, non-inferiority, exploratory)	5
**Methods: Participants, interventions, and outcomes**			
Study setting	#9	Description of study settings (eg, community clinic, academic hospital) and list of countries where data will	6

For peer review only

		be collected. Reference to where list of study sites can be obtained	
Eligibility criteria	#10	Inclusion and exclusion criteria for participants. If applicable, eligibility criteria for study centres and individuals who will perform the interventions (eg, surgeons, psychotherapists)	6
Interventions: description	#11a	Interventions for each group with sufficient detail to allow replication, including how and when they will be administered	7
Interventions: modifications	#11b	Criteria for discontinuing or modifying allocated interventions for a given trial participant (eg, drug dose change in response to harms, participant request, or improving / worsening disease)	N/A
Interventions: adherance	#11c	Strategies to improve adherence to intervention protocols, and any procedures for monitoring adherence (eg, drug tablet return; laboratory tests)	8 & 16
Interventions: concomitant care	#11d	Relevant concomitant care and interventions that are permitted or prohibited during the trial	N/A
Outcomes	#12	Primary, secondary, and other outcomes, including the specific measurement variable (eg, systolic blood pressure), analysis metric (eg, change from baseline, final value, time to event), method of aggregation (eg, median, proportion), and time point for each outcome.	8-11

For peer review only

		Explanation of the clinical relevance of chosen efficacy and harm outcomes is strongly recommended	
Participant timeline	#13	Time schedule of enrolment, interventions (including any run-ins and washouts), assessments, and visits for participants. A schematic diagram is highly recommended (see Figure)	10 & FIG 1
Sample size	#14	Estimated number of participants needed to achieve study objectives and how it was determined, including clinical and statistical assumptions supporting any sample size calculations	11-12
Recruitment	#15	Strategies for achieving adequate participant enrolment to reach target sample size	12-13
**Methods: Assignment of interventions (for controlled trials)**			
Allocation: sequence generation	#16a	Method of generating the allocation sequence (eg, computer-generated random numbers), and list of any factors for stratification. To reduce predictability of a random sequence, details of any planned restriction (eg, blocking) should be provided in a separate document that is unavailable to those who enrol participants or assign interventions	13

For peer review only

Allocation concealment mechanism	#16b	Mechanism of implementing the allocation sequence (eg, central telephone; sequentially numbered, opaque, sealed envelopes), describing any steps to conceal the sequence until interventions are assigned	13
Allocation: implementation	#16c	Who will generate the allocation sequence, who will enrol participants, and who will assign participants to interventions	13
Blinding (masking)	#17a	Who will be blinded after assignment to interventions (eg, trial participants, care providers, outcome assessors, data analysts), and how	N/A
Blinding (masking): emergency unblinding	#17b	If blinded, circumstances under which unblinding is permissible, and procedure for revealing a participant's allocated intervention during the trial	N/A
**Methods: Data collection, management, and analysis**			
Data collection plan	#18a	Plans for assessment and collection of outcome, baseline, and other trial data, including any related processes to promote data quality (eg, duplicate measurements, training of assessors) and a description of study instruments (eg, questionnaires, laboratory tests) along with their reliability and validity,	13-14

For peer review only

		if known. Reference to where data collection forms can be found, if not in the protocol	
Data collection plan: retention	#18b	Plans to promote participant retention and complete follow-up, including list of any outcome data to be collected for participants who discontinue or deviate from intervention protocols	13-14
Data management	#19	Plans for data entry, coding, security, and storage, including any related processes to promote data quality (eg, double data entry; range checks for data values). Reference to where details of data management procedures can be found, if not in the protocol	14
Statistics: outcomes	#20a	Statistical methods for analysing primary and secondary outcomes. Reference to where other details of the statistical analysis plan can be found, if not in the protocol	14-15
Statistics: additional analyses	#20b	Methods for any additional analyses (eg, subgroup and adjusted analyses)	14-15
Statistics: analysis population and missing data	#20c	Definition of analysis population relating to protocol non-adherence (eg, as randomised analysis), and any statistical methods to handle missing data (eg, multiple imputation)	14-15

Methods: Monitoring

For peer review only

Data monitoring: formal committee	#21a	Composition of data monitoring committee (DMC); summary of its role and reporting structure; statement of whether it is independent from the sponsor and competing interests; and reference to where further details about its charter can be found, if not in the protocol. Alternatively, an explanation of why a DMC is not needed	15-16
Data monitoring: interim analysis	#21b	Description of any interim analyses and stopping guidelines, including who will have access to these interim results and make the final decision to terminate the trial	15-16
Harms	#22	Plans for collecting, assessing, reporting, and managing solicited and spontaneously reported adverse events and other unintended effects of trial interventions or trial conduct	16
Auditing	#23	Frequency and procedures for auditing trial conduct, if any, and whether the process will be independent from investigators and the sponsor	15-16
**Ethics and dissemination**			
Research ethics approval	#24	Plans for seeking research ethics committee / institutional review board (REC / IRB) approval	16-17

For peer review only

Protocol amendments	#25	Plans for communicating important protocol modifications (eg, changes to eligibility criteria, outcomes, analyses) to relevant parties (eg, investigators, REC / IRBs, trial participants, trial registries, journals, regulators)	16-17
Consent or assent	#26a	Who will obtain informed consent or assent from potential trial participants or authorised surrogates, and how (see Item 32)	12-13
Consent or assent: ancillary studies	#26b	Additional consent provisions for collection and use of participant data and biological specimens in ancillary studies, if applicable	N/A
Confidentiality	#27	How personal information about potential and enrolled participants will be collected, shared, and maintained in order to protect confidentiality before, during, and after the trial	17
Declaration of interests	#28	Financial and other competing interests for principal investigators for the overall trial and each study site	21-22
Data access	#29	Statement of who will have access to the final trial dataset, and disclosure of contractual agreements that limit such access for investigators	22
Ancillary and post trial care	#30	Provisions, if any, for ancillary and post-trial care, and for compensation to those who suffer harm from trial participation	N/A

For peer review only

Dissemination policy: trial results	#31a	Plans for investigators and sponsor to communicate trial results to participants, healthcare professionals, the public, and other relevant groups (eg, via publication, reporting in results databases, or other data sharing arrangements), including any publication restrictions	16-17
Dissemination policy: authorship	#31b	Authorship eligibility guidelines and any intended use of professional writers	22
Dissemination policy: reproducible research	#31c	Plans, if any, for granting public access to the full protocol, participant-level dataset, and statistical code	N/A

Appendices

Informed consent materials	#32	Model consent form and other related documentation given to participants and authorised surrogates	APPENDIX 1
Biological specimens	#33	Plans for collection, laboratory evaluation, and storage of biological specimens for genetic or molecular analysis in the current trial and for future use in ancillary studies, if applicable	N/A

None The SPIRIT checklist is distributed under the terms of the Creative Commons Attribution License CC-BY-ND 3.0. This checklist can be completed online using https://www.goodreports.org/, a tool made by the EQUATOR Network in collaboration with Penelope.ai

For peer review only - http://bmjopen.bmj.com/site/about/guidelines.xhtml

BMJ Open

Usual care and a self-management support programme vs usual care and a relaxation programme for people living chronic headache disorders: a randomised controlled trial protocol (CHESS)

Journal:	*BMJ Open*
Manuscript ID	bmjopen-2019-033520.R2
Article Type:	Protocol
Date Submitted by the Author:	17-Dec-2019
Complete List of Authors:	Patel, Shilpa; University of Warwick, Warwick Medical School, Clinical Trials Unit<br>Carnes, Dawn; Queen Mary University of London, Centre for Primary Care and Public Health<br>Eldridge, Sandra; Queen Mary University of London, Centre for Primary Care and Public Health<br>Ellard, David; University of Warwick, Warwick Medical School, Clinical Trials Unit<br>Griffiths, Frances; University of Warwick, Warwick Medical School, Division of Health Sciences<br>Haywood, Kirstie; University of Warwick, Warwick Medical School, Division of Health Sciences<br>Hee, Siew Wan; University of Warwick, Warwick Medical School<br>Mistry, Dipesh; University of Warwick, Warwick Medical School, Clinical Trials Unit<br>Mistry, Hema; University of Warwick, Warwick Medical School, Warwick Evidence<br>Nichols, Vivien; University of Warwick, Warwick Medical School, Clinical Trials Unit<br>Petrou, Stavros; University of Warwick, Warwick Medical School<br>Pincus, Tamar; Royal Holloway University of London, Psychology<br>Potter, Rachel; University of Warwick, Warwick Medical School, Clinical Trials Unit<br>Sandhu, Harbinder; University of Warwick Warwick Medical School, Clinical Trials Unit<br>Taylor, Stephanie; Queen Mary University of London, Centre for Primary Care and Public Health<br>Underwood, Martin; University of Warwick, Warwick Medical School, Clinical Trials Unit<br>White, Kimberley; University of Warwick, Warwick Medical School, Clinical Trials Unit<br>Matharu, Manjit; University College London Hospitals NHS Foundation Trust National Hospital for Neurology and Neurosurgery
**Primary Subject Heading**:	Neurology
Secondary Subject Heading:	General practice / Family practice, Health services research

For peer review only - http://bmjopen.bmj.com/site/about/guidelines.xhtml

Keywords:	chronic headache, tension type headache, Migraine < NEUROLOGY, self-management, PRIMARY CARE, study protocol

SCHOLARONE™
Manuscripts

TITLE: Usual care and a self-management support programme vs usual care and a relaxation programme for people living chronic headache disorders: a randomised controlled trial protocol (CHESS)

AUTHORS:
Shilpa Patel, Warwick Clinical Trials Unit, Warwick Medical School, University of Warwick, Coventry. CV4 7AL, UK Shilpa.Patel@warwick.ac.uk

Dawn Carnes, Centre for Primary Care and Public Health, Blizard Institute Barts and The London School of Medicine and Dentistry, Queen Mary University of London, London, UK d.carnes@qmul.ac.uk

Sandra Eldridge, Centre for Primary Care and Public Health, Blizard Institute Barts and The London School of Medicine and Dentistry, Queen Mary University of London, London, UK s.eldridge@qmul.ac.uk

David Ellard, Warwick Clinical Trials Unit, Warwick Medical School, University of Warwick, Coventry, CV4 7AL, UK D.R.Ellard@warwick.ac.uk

Frances Griffiths, Health Sciences, Warwick Medical School, University of Warwick, CV4 7AL, UK f.e.griffiths@warwick.ac.uk

Kirstie Haywood, Division of Health Sciences, Warwick Medical School, University of Warwick, Coventry, CV4 7A, UK K.L.Haywood@warwick.ac.uk

Siew Wan Hee, Statistics and Epidemiology Unit, Warwick Medical School, University of Warwick, CV4 7AL, UK s.w.hee@warwick.ac.uk

Dipesh Mistry, Warwick Clinical Trials Unit, Warwick Medical School, University of Warwick, Coventry, CV4 7AL, UK D.Mistry@warwick.ac.uk

Hema Mistry, Population Evidence and Technologies, Warwick Medical School, University of Warwick, Coventry, CV4 7AL, UK Hema.Mistry@warwick.ac.uk

Vivien Nichols, Warwick Clinical Trials Unit, Warwick Medical School, University of Warwick, Coventry, CV4 7AL, UK V.P.Nichols@warwick.ac.uk

Stavros Petrou, Warwick Clinical Trials Unit, Warwick Medical School, University of Warwick, Coventry, CV4 7AL, UK S.Petrou@warwick.ac.uk

Tamar Pincus, Royal Holloway, University of London, Department of Psychology, Surrey, TW20 0EX, UK T.Pincus@rhul.ac.uk

Rachel Potter, Warwick Clinical Trials Unit, Warwick Medical School, University of Warwick, Coventry, CV4 7AL, UK r.potter@warwick.ac.uk

Harbinder Sandhu, Warwick Clinical Trials Unit, Warwick Medical School, University of Warwick, Coventry, CV4 7AL, UK Harbinder.K.Sandhu@warwick.ac.uk

For peer review only - http://bmjopen.bmj.com/site/about/guidelines.xhtml

Stephanie JC Taylor, Centre for Primary Care and Public Health, Blizard Institute Barts and The London School of Medicine and Dentistry, Queen Mary University of London, London, UK s.j.c.taylor@qmul.ac.uk

Martin Underwood, Warwick Clinical Trials Unit, Warwick Medical School, University of Warwick, Coventry, CV4 7AL, UK M.Underwood@warwick.ac.uk

Kimberley White, Warwick Clinical Trials Unit, Warwick Medical School, University of Warwick, Coventry, CV4 7AL, UK Kimberley.White@warwick.ac.uk

Manjit S Matharu, Headache Group, UCL Institute of Neurology and The National Hospital for Neurology and Neurosurgery, Queen Square, London, WC1N 3BG, UK manjit.matharu@nhs.net

On behalf of the CHESS team.

Corresponding author:
Shilpa Patel, Warwick Clinical Trials Unit, Warwick Medical School, University of Warwick, Coventry. CV4 7AL Shilpa.Patel@warwick.ac.uk
Tel: 44 (0) 2476 150 405

For peer review only

ABSTRACT

Introduction: Chronic headaches are poorly diagnosed and managed, and can be exacerbated by medication overuse. There is insufficient evidence on the non-pharmacological approaches to helping people living with chronic headaches.

Methods and analysis: CHESS (Chronic Headache Education and Self-management Study) is a pragmatic randomised controlled trial to test the effectiveness and cost effectiveness of a self-management education support programme on top of usual care for patients with chronic headaches against a control of usual care and relaxation. The intervention is a two day group course based on education, personal reflection and a cognitive behavioural approach, plus a nurse-led one-to-one consultation and follow up over 8 weeks. We aim to recruit 689 participants (356 to the intervention arm and 333 to the control) from primary care and self-referral in London and the Midlands. The trial is powered to show a difference of 2.0 points on the Headache Impact Test (HIT6); a patient reported outcome measure at 12 months post randomisation. Secondary outcomes include health related quality of life, self-efficacy, social activation and engagement, anxiety and depression and health care utilisation. Outcomes are being measured at four, eight and 12 months. Cost-effectiveness will be expressed in terms of incremental cost per quality-adjusted life year gained.

Ethics and dissemination: This trial will provide data on effectiveness and cost-effectiveness of a self-management support programme for chronic headaches. The results will inform commissioning of services and clinical practice. North West - Greater Manchester East Research Ethics Committee have approved the trial. The current protocol version is 3.6 date 7th March 2019.

Trial registration number: ISRCTN79708100. Registered 16th December 2015, http://www.isrctn.com/ISRCTN79708100

Keywords: Chronic headache, tension type headache, migraine, self-management, primary care, study protocol

For peer review only

For peer review only - http://bmjopen.bmj.com/site/about/guidelines.xhtml

ARTICLE SUMMARY

Strengths and limitations of the study

- This trial presents a high quality randomised controlled trial designed to determine the effectiveness and cost-effectiveness of a group self-management support programme for adults with chronic headaches.
- The intervention underpinning the trial is a complex intervention which has been designed using the best available evidence and theory. The full details of the intervention development and design have been peer reviewed and published.
- A separate mixed-methods process evaluation is running alongside the trial and will explore fidelity of the intervention and implementation. A detailed process evaluation protocol has been peer reviewed and published.
- The success of the trial will be dependent on the ability to deliver this complex group intervention, ensure fidelity and subsequently obtain follow-up data in this population.

For peer review only

INTRODUCTION

Headache disorders are a major cause of pain and disability.[1] A definition of a chronic headache disorder used in epidemiological studies is that the person has headache for 15 or more days per month for at least three months.[2-4] Other authors prefer the term chronic daily headache but this is less clearly defined.[5] Chronic headache disorders mainly affect the young adult population, many of whom have both work and family commitments.[6] The most common chronic headache disorders are chronic tension type headache, chronic migraine, and medication overuse headaches (MOH).[1] Tension type headache and migraine are primary headaches. Medication overuse is a secondary headache that can develop in people with frequent acute headaches who take analgesics, or specific anti-migraine compounds (e.g. triptans) for ≥10 days per month.

Around 2-4% of the global population experience chronic daily headaches.[7] Between a quarter and half of those affected also have MOH, which has a prevalence of 1%.[8-10] Many people with chronic headache have undiagnosed chronic migraine.[11] More appropriate use of pharmacological treatment has the potential to improve outcomes for people living with chronic headache disorders. This might mean introducing migraine prophylactic drugs or using fewer analgesics/triptans. Depression, anxiety, poor sleep, stress and poor self-efficacy for managing headaches are prognostic markers for a poor outcome in chronic headache disorders.[12] Limited qualitative data indicate that chronic headache disorders can directly and indirectly drive behaviour: that people live with the 'spectre of headache' (an array of concerns people with chronic headaches have to take into account when forward planning) and that headaches lead to strained relationships.[13] For people living with chronic migraine or chronic tension type headache non-pharmacological self-management approaches may improve headache related symptoms, whilst not affecting headache frequency.[14] Group interventions using a cognitive behavioural approach, mindfulness and educational components may be more effective than alternative interventions.[14]

Supportive self-management approaches are well established in the management of several chronic painful disorders, but this is not the case for chronic headache disorders.[15-17] The National Institute for Health and Care Excellence (NICE) guidance in England, for example, only makes one positive recommendation for a non-pharmacological treatment for people living with chronic migraine or chronic tension type headache; to consider a course of acupuncture for people with chronic migraine or chronic tension type headache.[18]

We describe here the protocol for a randomised controlled trial (RCT) to estimate the effectiveness and cost-effectiveness of a group self-management support programme for adults with chronic headaches arising from migraine or tension type headache, with or without medication overuse. The CHESS intervention consists of a group education and self-management support programme plus a tailored one-to-one headache consultation exploring medication optimisation, lifestyle factors and goals. This active arm of the trial is being compared to usual care plus a relaxation programme.

METHODS AND ANALYSIS

The methods section is structured in accordance with the SPIRIT 2013 recommendations.[19] Here we provide an overview of the trial plus a brief description of the proposed health economic evaluation, process evaluation and patient and public involvement.

The primary objective is to estimate the clinical and cost-effectiveness of a group education and self-management programme for people living with chronic headache recruited from primary care when compared to a GP care plus relaxation control group.

1. Participants, interventions and outcomes

For peer review only - http://bmjopen.bmj.com/site/about/guidelines.xhtml

Study setting
Primary care settings in two localities; London and the Midlands.

Eligibility criteria
The population of interest are adults living with chronic migraine or chronic tension type headache, with or without medication overuse. Part of the challenge in interpreting the findings of research in people living with chronic headaches is the often poor reporting of participants' phenotypic characteristics, meaning it is not possible to draw conclusions for specific chronic headache types.[13 20] For this trial, it is important that we are able to define our participants' headache type, or types, accurately and exclude those with non-eligible headache types. We, therefore developed and validated a logic model for use in a telephone interview by a nurse (non-headache expert) that would allow us to identify and classify people meeting our entry criteria, whilst also excluding people with secondary headaches, other than medication overuse and other causes of primary headaches (Table 1).[21]

Table 1: Inclusion and exclusion criteria

Inclusion criteria	Exclusion criteria
1. Able and willing to comply with the study procedures and provide written informed consent<br><br>2. Aged ≥18 years (no upper limit)<br><br>3. Living with chronic headache; defined as headache on 15 or more days per month for at least the preceding three months<br><br>4. The nurse telephone classification interview confirms headache type to be chronic migraine, or chronic tension type headache, with or without medication overuse headache<br><br>5. Fluent in written and spoken English.	1. Unable to attend the group sessions<br><br>2. No access to a telephone (for classification interview)<br><br>3. Has an underlying serious psychological disorder with ongoing symptoms that preclude or significantly interfere with participation in the group intervention<br><br>4. Previous entry or randomisation in the present trial<br><br>5. Currently participating in another clinical trial of headache treatments or unregistered medicinal product, or less than 90 days have passed since completing participation in such a trial.

Pregnancy is not an exclusion criterion. However, any pregnant women randomised to the active intervention will be advised to speak to their General Practitioner (GP) with regards to medication and the CHESS intervention nurses will not discuss this with them during the consultation for safety reasons.

Our previous experience is that the challenges of running non-English language group interventions for chronic painful disorders are too great to do successfully within a randomised controlled trial.[22] Furthermore the main outcomes are not validated in those languages other than English which are relevant to a UK context. For these reasons we are excluding people who are not fluent in written and spoken English.

For peer review only - http://bmjopen.bmj.com/site/about/guidelines.xhtml

In this pragmatic trial our entry criteria reflect the point in the care pathway where our intervention will be offered. Specifically, general practitioners will refer people they identify with chronic headaches into the service. Thus, there is not the pre-randomisation run-in one might expect in a more explanatory drug trial and we base assessment of the presence of chronic headache on a single telephone assessment and include a mixture of headache types.

Interventions

The intervention consists of a group education and self-management programme, an eight-week headache paper diary, a one-to-one nurse-led consultation and follow-up telephone calls, where necessary, for up to eight weeks. We have described its development in detail elsewhere.[23] We summarise it briefly here.

The programme is run over two days in a two week period for around 10 participants per group (target six to twelve participants), is facilitated by two intervention trained health care professionals, at least one of whom is a nurse (the second facilitator could be a nurse or other registered allied health professional such as psychologist, physiotherapist, chiropractor, occupational therapist). We originally planned to run sessions using a nurse, and a lay facilitator living with chronic headaches. However, in our feasibility study we found it difficult to recruit people living with chronic headaches to act as facilitators and of those that were recruited several found it difficult to commit to facilitation of courses because of the unpredictability of their condition. The intervention builds on a previously developed and tested educational and cognitive behavioural self-management intervention for people with chronic musculoskeletal pain.[24]

The aim of the course is to encourage and enable those with chronic headaches to recognise unhelpful thought patterns and behaviours that contribute to their headache burden and to do something about them. The course provides participants with an overall toolbox of strategies that could help in the management of their headaches. These strategies include psychological techniques to change perceptions and feelings about living with chronic headaches as well as more practical strategies around lifestyle factors and medication. The group intervention is delivered using a range of methods including: group discussions, ideas generation, sharing narratives and experiences, problem solving, role play and taster activity sessions. The programme includes a range of behaviour change techniques including; barrier identification, general encouragement, instruction from the group facilitators, provision of feedback, and allowing opportunities for social comparison in the group. We also include an educational video (available on disc and on-line) following feedback from our patient and public involvement (PPI) group on the importance of having something to show family and friends about their 'invisible' disorder.

The sessions take place on weekdays and, where possible, during school hours to accommodate those with child care responsibilities. Sessions are held in easily accessible community venues and GP practices.

Participants in the intervention arm are asked to complete a paper headache diary for a period of up to eight weeks prior to the two-day programme. A one-to-one, individually tailored nurse-led consultation follows the two-day programme. During this session the nurse classifies the participant's headache type, discuss medication and lifestyle factors and explore participants' goals. This discussion is backed up by written information (for patient and GP), consistent with NICE guidance, to support shared informed decision making between the patient and their GP, about medication choices.[25] All participants will be offered telephone follow-up for up to eight weeks. The frequency of these follow-up calls will be individually negotiated and agreed with participants during the one-to-one consultation. Full details of the intervention content have been published elsewhere.[23]

Control intervention

For peer review only - http://bmjopen.bmj.com/site/about/guidelines.xhtml

Previous studies of this nature have reported that a usual care control arm was not an incentive to join the study.[15] We know from experience that patients enjoy the relaxation part of pain self-management programmes.[17] Therefore as an incentive to participate, the control group receive standard usual care plus a relaxation CD and instructions for use over the duration of the study, up until final follow-up at 12 months. Participants are free to continue using the relaxation CD thereafter should they wish.

As all participants in the trial have a headache classification interview prior to randomisation, we feed back the results of that classification interview to control participants, and their GPs, together with advice on headache management. In this way we ensure all participants receive best usual care.

The relaxation CD or downloadable MPG file (from the CHESS website) consists of a 17-minute relaxation audio script that starts by focusing on the breath before talking the participant through progressive relaxation of muscles. The participants in the control group choose when, where and how often they do the relaxation but are advised to try it two to three times a week, or as often as they feel appropriate over the course of the study.

Facilitator recruitment and training
All group sessions have two facilitators, one of whom must be a registered nurse, the other facilitator is any allied health care professional, registered with a regulatory body, with an interest in this patient population and condition. Facilitators were recruited via advertisements in allied health care profession organisations and trained over a two-day period to deliver the self-management intervention. Nurses received a further day of training to cover the one-to-one consultations. The training course covers the content of the intervention, facilitation skills, managing groups and trial procedures. Those undergoing the training were assessed using a learning assessment form at the end of the training course to check their knowledge and understanding. Those evaluated to be competent were asked to deliver the intervention. Those who either struggled with some elements of the content or expressed any concerns about delivering the intervention were provided with additional one to one support by the trainers.

Intervention quality assessment
Quality assurance of the intervention is important to ensure that the trial intervention is delivered consistently and in accordance with the trial protocol and manuals. As part of the intervention development we have produced two very detailed manuals, the first which covers the two days of group sessions and the second which details the process for the one to one consultations. All facilitators have been appropriately trained on the delivery of the intervention in accordance with the manuals. They have been instructed to use the manuals to guide them through delivery of the intervention.

To quality assure the course delivery we will aim to observe each facilitator. We will observe each facilitator early in their facilitation to allow any difficulties or challenges to be addressed. Subsequently they will be observed mid-way through. Observations will be by session and the number of sessions observed will depend on the ability of the observer to capture the required information.

The observations will be conducted by members of the CHESS team who have knowledge of the intervention and its delivery. Observers will complete an observation form which will address facilitator skills and adherence to content delivery. Feedback will be provided on the day where possible. If this is not possible the observer will arrange to contact the facilitator by phone. The study team will discuss any difficulties with the facilitator to minimise impact on the rest of the course and to help with the delivery of future courses. For any facilitators struggling the central research team

For peer review only - http://bmjopen.bmj.com/site/about/guidelines.xhtml

will monitor their personal reflections for the remaining duration of the course and follow-up with phone calls if required.

Outcome measures - Primary outcome measure; headache related quality of life

Our primary outcomes is headache related quality of life assessed at the primary endpoint, 12 months after randomisation. For sample size determination we have specified the Headache Impact Test (HIT-6), a 6-item patient-reported outcome measure (PROM), as our measure of the primary outcome.[26] The HIT-6 provides a short overall assessment of headache impact – with items assessing fatigue, pain, social functioning, emotional well-being and cognition. Prior to selecting our outcome measures, we did a systematic review of patient reported outcome measures for headaches.[27] Only for the HIT-6 did we find acceptable evidence supporting its use for our target population who may not have been given a headache diagnosis prior to study entry.

Another measure, shortlisted in the review, was the 14-item Migraine-Specific Quality of Life Questionnaire (MSQ v2.1) assesses the role restrictions, limitations and emotional impact of migraine.[27] The MSQv2.1 has acceptable evidence of psychometric properties following completion in a migraine population.[27-30]

To inform our selection of outcome measures we undertook interviews (n=14) with people with chronic headache. Our participants described greater perceived relevance of the MSQ(v2.1) compared to the HIT-6. With permission from GSK, the copyright holders, we modified the MSQ (v2.1), changing the focus from 'migraine' to 'headache'; the modified measure was renamed as the 'Chronic Headache Quality of Life Questionnaire' (CHQLQ v1.0). In our ongoing work we are assessing the performance of this modified instrument. In the event its performance is superior to the HIT-6 we will consider changing the primary outcome.

Both the HIT-6 and the CHQLQ v1.0 will be collected via postal questionnaire at baseline, four, eight and 12 months post randomisation.

Outcome measures - other secondary outcomes measures

The following secondary outcome measures are being collected:

1. *Headache days:* Our main outcome describing headaches days will be headaches days in the preceding month reported at baseline and follow-up postal questionnaires. We will also estimate total headaches days over the whole study period from patient reported data collected via a smartphone app.
2. *Headache impact:* We will assess headache impact based on their typical duration and severity reported in baseline and follow-up questionnaires
3. *Composite headache outcome:* We will produce a composite headache outcome of headache days x severity x duration using data from the smartphone app and questionnaires.
4. *Generic health-related quality of life*: We have included two standard measures of health-related quality of life – the SF-12 V2 and EQ-5D-5L.[31-33] There is limited, but acceptable, evidence supporting the use of the SF-12 V2 to assess overall quality of life in a headache population.[27] There is no such evidence for the EQ-5D-5L.[27] We will therefore, use EQ-5D-5L primarily for our health economic analyses.[32]
5. *Emotional well-being:* Hospital Anxiety and Depression Scale (HADs) - Psychological distress is extremely common in people living with chronic pain. HADs has been used in many previous studies of chronic pain, including the COPERS study where we identified positive effects on both anxiety and depression in a chronic pain population.[22 34]
6. *Self-Efficacy:* Pain Self-Efficacy Questionnaire (PSEQ) - Self-efficacy is an important mediator for how self-management interventions may improve patient outcomes. It is important, therefore, to measure change in self-efficacy as part of understanding the causal pathway for

For peer review only - http://bmjopen.bmj.com/site/about/guidelines.xhtml

any change and informing our process evaluation.[35] We have previously reviewed measures of self-efficacy and concluded that PSEQ is the most appropriate choice for studies of this nature; although all current measures have limitations.[36]

7. *Social Activity: Social Integration Subscale of the Health Education Impact Questionnaire (heiQ)* - Chronic headache can result in a disrupted lifestyle and a reduced quality of life both during and between attacks; the impact of chronic headache on an individual's ability to commit to social plans is an important aspect of quality of life. Successful treatment should seek to improve both overall quality of life, as well as an individual's quality of life during the attack, including their ability to integrate in society. A well-developed, condition-specific measure should seek to capture these distinctions. The five-item Social Integration Subscale (SIS) is one of eight domains contained within the heiQ, a measure of the impact of patient education programmes in chronic conditions.[37]
8. *Bodily pain:* Chronic headache commonly co-exists with other chronic painful disorders such as low back pain.[38-41] The CHESS intervention might affect the troublesomeness of other bodily pains. We will collect these data using a previously validated Troublesomeness Grid.[42]

At baseline we also collect data on age, gender, ethnic group, age at leaving full time education and current work status.

We will collect follow-up data at four, eight, and 12 months after randomisation by postal questionnaire survey. A £5 high street voucher is enclosed as a token of our appreciation at each initial time point. We will send out two postal follow-up reminders. In the event that no response is obtained, we will aim to collect our primary clinical outcome data by telephone. This includes the HIT-6 and EQ-5D-5L.

The smartphone data will be collected weekly for six months from initial eligibility and then monthly until 12 months after randomisation. A paper version of the app will be available to those that do not use a smartphone.

If there are missing data (for our key clinical outcomes), this will be followed up with the participant who completed the form, as soon as possible by a member of the team over the phone. We will phone the participant and enter the correct information onto the form, this will be initialled and dated.

Participant timelines

Figure 1 shows the recruitment flow and table 2 shows the study timelines.

Figure 1 – here

Table 2: Schedule of enrolment, interventions, and assessments

Activity	Pre-enrolment to allocation	Randomisation to 12 weeks	4 months	8 months	12 months
Expression of interest	X				
Trial information	X				

For peer review only - http://bmjopen.bmj.com/site/about/guidelines.xhtml

Download app to record headache patterns (weekly for six months and then monthly until the end of follow-up at 12 months)	X	X	X	X	X
Questionnaire Demographic information, HIT-6, CHQLQ, headache days, SF12, EQ-5D-5L, HADs, PSEQ, HeiQ, troublesomeness	X				
Consent	X				
Headache classification interview	X				
Allocation		X			
Intervention plus completion of paper headache diary for duration of up to eight weeks before attending group		X			
Control		X			
HIT-6, CHQLQ, headache days, SF12, EQ-5D-5L, HADs, PSEQ, HeiQ, troublesomeness			X		
2 postal follow up reminders and phone call for PO if needed			X		
HIT-6, CHQLQ, headache days, SF12, EQ-5D-5L, HADs, PSEQ, HeiQ, troublesomeness				X	
2 postal follow up reminders and phone call for PO if needed				X	
HIT-6, CHQLQ, headache days, SF12, EQ-5D-5L, HADs, PSEQ, HeiQ, troublesomeness					X
2 postal follow up reminders and phone call for PO if needed					X

HIT-6 (Headache Impact Test),[26] CHQLQ (Chronic Headache Quality of Life Questionnaire), SF12 (Short Form 12-item Health Survey),[31 33] EQ-5D-5L (EuroQoL),[32] HADs (Hospital Anxiety and Depression Scale),[34] PSEQ (Pain Self-Efficacy Questionnaire),[35] HeiQ (Health Education Impact Questionnaire)[37]

Sample size

For the purposes of our sample size calculation, the primary clinical outcome is the difference in mean HIT-6 score at 12 months post randomisation between the self-management group programme and the usual care relaxation therapy (control arm). The HIT-6 outcome measure is a continuous scale with higher values indicating more severe impact on daily life. From our systematic reviews, we anticipate a worthwhile difference to be 2.0, i.e. mean outcome in the control arm is 2.0 units higher than for the intervention.[27 43] In our feasibility study (N=114), the standard deviation of the HIT-6 score at baseline was 6.87.[44]

Participants are randomised to either the self-management group or usual care and relaxation therapy. In this design, there may be a clustering effect in the self-management group and not in the control arm, which needs to be allowed for in the sample size calculation. Based on similar trials we assume that the intra-class correlation coefficient (ICC) is 0.01.[22] The anticipated average size of the self-management programme is 10.

The minimum sample size required was estimated using Moerbeek and Wong's method to account for grouping in one arm.[45] To detect a between-group difference of 2.0 with standard deviation of 6.9 (a standardised mean difference of 0.29) and assuming that the ratio of the total variance in the self-management group to the relaxation therapy is 1 at the two-sided 5% significance level and at least 90% power, the sample size required is 523 participants (253 in the relaxation group and 270 in the self-management group).

For peer review only

In the feasibility study, the overwhelming majority of those recruited, approximately 95%, chronic migraine; just 5% had chronic tension type headache. We want to be able to draw definite conclusions on the specific subgroup of chronic migraine. Therefore, we will base our sample size and primary clinical outcome on the population with chronic migraine. Therefore, based on 95% of our sampled population with chronic migraine and accounting for a 20% loss to follow-up as above, the sample size we would require is 689 with 333 to the relaxation arm and 356 to the self-management programme.

In the feasibility study we recruited around 1/1,000 of practice population to take part,[44] we therefore anticipate we need to recruit from around 100 general practices with a combined list size of around 700,000.

In consultation with the Data Monitoring and Ethics Committee (DMEC), we will review the sample size around halfway through recruitment to ensure we have recruited sufficient participants with chronic migraine and revise our estimates using within trial data on the variance of our primary outcome at baseline. This review will be based on the headache classification and actual baseline standard deviation of our sampled population. We might also need to recruit some additional participants to ensure that the final group sessions at each site are adequately populated.

Recruitment

Participants are identified and invited into the study in two ways. Firstly, practices run electronic searches on their databases, to identify people who have consulted with headaches or have been prescribed migraine specific drugs (e.g. triptans, pizotifen) in the preceding two years. Practices then screen the lists for those it would be inappropriate to approach (e.g. poorly controlled serious mental illness, terminal illness, or known secondary causes of headache such as primary or secondary brain tumours), and send approach letters to the remainder.

People can self-refer to the trial. Participating general practices, the principal pharmacies used by their patients, are supplied with a study poster for display in patient areas inviting people to contact the study team if they have headaches and are interested in participating. Information about the trial is made available on the poster (in general practices and pharmacies), and websites (https://warwick.ac.uk/fac/med/research/ctu/trials/other/chess/ and https://warwick.ac.uk/fac/med/research/ctu/trials/chess. People who find about the trial through the internet or following media exposure, and can travel to sessions, can also self-refer to the trial.

Using both approaches will allow people receiving GP treatment for chronic headaches who are not coded in the GP system as having headaches, and those who are self-medicating their headaches, the opportunity to join the study. We anticipate that we will primarily recruit people registered with participating practices; however, we will not restrict recruitment to those registered with participating practices.

In addition to these two main recruitment strategies, a study press release was submitted in January 2018 which was picked up by various media outlets and generated further interest in the study from potential participants.

The study co-ordinating team contact people expressing an interest in the study and check that they are eligible, explain the study, and obtain participant's verbal consent to start completing an electronic headache symptom severity, duration & frequency diary (or paper version where there is no access to the internet). The electronic diary is completed weekly for the first six months and subsequently monthly until the end of follow-up at 12 months. The electronic diary is used to identify any early effects of rebound headache in those with medication overuse headache. At this time participants are sent a baseline questionnaire and consent form to return in a freepost envelope.

For peer review only - http://bmjopen.bmj.com/site/about/guidelines.xhtml

Following receipt of baseline questionnaire and consent form participants are contacted by phone for a nurse headache classification interview. People whose headache is confirmed to meet our entry criteria are then eligible for randomisation. If the nurse has a concern that the potential participant may have another headache type there is a second interview with a doctor skilled in the management of headache disorders from the National Migraine Centre (http://www.nationalmigrainecentre.org.uk/). This is to ensure no one is inappropriately excluded and that people with a headache type needing more specific treatment are directed towards appropriate treatment. For example, 2/108 people in our feasibility study had cluster headaches.[44] In such cases we write to the GP and the participant with details on the excluded headache type and explain that they no longer fit the inclusion criteria for the CHESS trial.

2. Assignment of interventions

Randomisation

The randomisation is stratified by geographical locality (Midlands and Greater London) and headache type (six possible headache types: chronic tension type headache, probable chronic migraine and definite chronic migraine with or without medication overuse headache) using minimisation. Randomisation takes place using an online application specifically developed for the CHESS Study by the Warwick Clinical Trials Unit (WCTU) programming team.

Randomisation takes place when there are around 20 participants who would be able to attend a local group if randomised to the active intervention. Typically these will come from 4-5 proximate general practices. However, people from other localities who are unable to attend a more local group and people who have self-referred to the study who are willing and able to travel to the sessions will also be included. By randomising in groups around two weeks prior to the start of a group, we are able to minimise any delay between randomisation and start of the intervention and ensure everyone has adequate follow-up from when they might have been exposed to the intervention. For those that are subsequently unable to attend after randomisation to a group, we will offer them another group where possible.

Allocation concealment and protection from bias

Allocation concealment will be maintained by using Warwick CTU's centralised randomisation service. All baseline data will be collected prior to randomisation. Blinding will be impossible for participants, facilitators and the central co-ordinating CHESS team. All data entry and checking is done blind to treatment allocation. The unbalanced randomisation means it is not possible blind the trial statistician to treatment allocation.

Our primary outcome is a participant completed outcome. Inevitably, participants will be aware of their treatment allocation. We will develop and sign off a detailed pre-specified statistical analysis plan before allocation codes are released.

3. Data collection, management and analysis

Data collection methods

Data will be collected using two methods, postal survey questionnaires and a smartphone application (App) for headaches. All participants will be asked to complete a smartphone app about their headaches. If they do not have access to a smartphone, or do not wish to use the app, a paper copy will be provided. Participants will initially complete the app weekly for up to six months, to cover any

For peer review only - http://bmjopen.bmj.com/site/about/guidelines.xhtml

period of withdrawal from medication, then monthly thereafter (still requiring them to reflect over the previous 7 days) until the end of the study at 12 months after randomisation. We are working with Clinvivo Ltd a University of Warwick spin-out company specialising in electronic data collection, to capture data on headache frequency, duration and severity electronically using a smartphone App (Table 3 shows the questions completed by participants and mode of response required). The data from the questions in the electronic diary will be numerical and downloaded into a WCTU database using a randomly generated 256-bit AES (Advanced Encryption Standard) key. At the end of the participants 12 months they will be provided with a summary report of their headache application data.

Reminders are sent to those participants who fail to downloaded the app or have not responded for more than three weeks. Reminders are sent by email or by post if there is no email address given.

A daily detailed paper headache diary is kept only by the intervention arm participants and the data will not be analysed by the study team but kept as a record by the participants and used during the one to one consultation where it will be collected and kept by the research team.

Table 3. Questions completed on the smartphone app.

Question	Mode of response
On how many of the last 7 days have you had a headache?	Insert number of headache days
On those days you had a headache, on average how long did they last?	Insert number of hours
On those days you had a headache on average how severe were they?	Scale of 0 to 10 (with 0 being no pain and 10 extremely severe pain)

Data management

All questionnaire data received by the trial team are reviewed for completeness and entered onto a secure, backed-up bespoke database held at WCTU which will be accessible only by authorised members of the team. Data from the smartphone App are downloaded securely (as described above) into the trial database. All data is checked when received, any queries relating to the HIT6 CHQLQ or EQ5D are raised with the participant by telephone.

Statistical analysis of effectiveness and harms

Our primary endpoint analyses will be based on the twelve month data.

Participants' characteristics and reported outcomes will be summarised as means and standard deviations (for continuous data) or frequencies and percentages (for categorical data) by treatment arms. Difference between baseline and the three follow-up time points (four-, eight- and 12-month post randomisation) will be computed for the primary and secondary outcomes by treatment arms.

The primary analysis approach will be intention to treat i.e. the data will be analysed according to the treatment the participant was originally allocated to, irrespective of what they actually received. We will explore the possibility of carrying out a complier averaged causal effect (CACE) analysis as a sensitivity analysis. Our primary analysis will be the difference between the self-management therapy (intervention) and the relaxation therapy (control) groups with a 95% confidence interval (CI) in the population with chronic migraine – if the proportion of participants with chronic tension type

For peer review only

headache is ≤ 15%.The hypothesis testing of the primary outcome will be two-sided at the 5% level and the main analysis will estimate the treatment effect using a multilevel model to account for clustering (the model used to design this main trial). We will also present results for the whole population (all headache types). If the proportion of chronic tension type headache is >15% then the primary analysis will be according to the whole population of chronic headache (chronic migraine and tension type headache). Our experience is that NICE, was specifically interested in data on specific headache types; excluding data that reported mixed population of people with chronic headaches. We will, therefore in addition to our primary analyses present the results (mean difference and 95% CI) for each of the two headache types with or without medication overuse headache separately, and present results for those with or without medication overuse separately to facilitate future meta-analyses and inform future condition specific guidelines. All analyses will be adjusted for the randomisation stratification factors (types of headache and geographical locality), sex, age and the baseline value of the dependent variable.

Similar analyses will be performed for all the other secondary outcomes. Pre-specified subgroup analyses using formal statistical tests for interaction will examine whether baseline anxiety, depression and severity are moderators of treatment effect.[46] We will assess the level of missingness in the primary outcome and if required, we will use appropriate multiple imputation techniques to impute data and estimate the treatment effect as a form of sensitivity analysis. A full statistical analysis plan, including all primary and secondary analyses, will be written and signed off prior to releasing allocation codes to analysts and others involved in developing the plan.

Using the data from the smartphone App we will generate a composite score for headache impact over the one-year of follow up as the function of headaches days x average duration x average severity. Presenting these data graphically will allow any early benefits or harms from the intervention to be identified.

Health Economic Evaluation

Our economic evaluation will be conducted alongside the trial and we will initially adopt a one-year time horizon from both an NHS and personal social services perspective and, separately for the purpose of a sensitivity analysis a broader societal perspective, to estimate the cost-utility of the intervention. Resource use data will be collected to explore the costs of the delivery of the intervention and to estimate the key cost drivers. This will mainly consist of visits to the GP practice, medication usage and any adverse events or length of stay in the hospital. In terms of costs to society, we will estimate time off work and any productivity losses associated with chronic headaches. Resource use information will be collected using self-completed postal questionnaires completed at four, eight and 12 months after randomisation, as well as the use of routine health service data collected from general practice records. Resource input will be valued using national estimates of unit costs such as the Prescription Cost Analysis database or the Unit Costs of Health and Social Care compendium.[47] Preference-based health-related quality of life outcomes will primarily be assessed through the completion of the EQ-5D-5L at each follow-up point.[48] Quality-adjusted life-years (QALYs) will be calculated as the area under the baseline-adjusted utility curve, and will be calculated using linear interpolation between baseline and follow-up utility scores.

The results of the economic evaluation will be presented using incremental cost-effectiveness ratios, expressed in terms of incremental cost per QALY gained, and cost-effectiveness acceptability curves generated via non-parametric bootstrapping.

More extensive economic modelling using decision-analytic methods will extend the target population, the time horizon to five years as the long-term natural history is unclear and the decision context, drawing on best available information from the literature together with stakeholder

For peer review only - http://bmjopen.bmj.com/site/about/guidelines.xhtml

consultations to supplement the trial data. Longer-term costs and consequences will be discounted to present values using nationally recommended discount rates recommended for health technology appraisal. We will use probabilistic sensitivity analysis to estimate the impact of uncertainty over model parameters. We will also use simple sensitivity analysis to assess the robustness of the results to changes in deterministic parameters such as medication dosages, costs, discount rate and time horizon for patients presenting with chronic headaches. We will also explore cost-effectiveness of the intervention by conducting subgroup analyses for the different headache types.

4. Monitoring, ethics and dissemination

Programme steering committee (PSC)

We have established a PSC to oversee the whole programme of work. The PSC functions specifically as a trial steering committee specifically for this trial. The PSC consists of seven members including two patient representative. The PSC will be responsible for oversight and monitoring and will consider any information from surveillance activity of other research in the field.

Data monitoring and Ethics Committee (DMEC)

A data monitoring and ethics committee has been convened consisting of an independent statistician, a trialist, a clinician and a lay member. Confidential reports that summarise the trial data and safety data will be reviewed by the DMC. The DMC will advise the PSC as to whether to continue, amend or terminate the trial based primarily on safety and efficacy considerations

Harms / Adverse Events (AE)

An adverse event or serious adverse event is any event that takes place on the way to, during, or on the way home from the intervention course. This includes the two days of the group course and the one to one nurse appointment. Our experience across multiple studies of group interventions is that adverse events that are directly attributable to the intervention are rare.

Events during the session, such as severe psychological disturbance, any mild or moderate levels of emotional distress as a result of discussing experiences of living with chronic headache, a fall during travel to and from the venue or any other adverse events, occurring during the delivery of the intervention will be reported to the trial co-coordinator by the intervention team. Any short-term increase in headaches as a consequence of medication withdrawal will be captured using the smartphone app (or paper version for those without access to a smartphone).

All adverse events will be managed in line with Warwick CTU's standard operating procedures.

Process Evaluation

A separate mixed-methods process evaluation is running alongside the trial. This will explore both the process of implementation the trial and the process of delivering and receiving the intervention. The process evaluation protocol is reported elsewhere.[49]

The fidelity of the delivery of the intervention will be assessed by this team in three ways, through: audio recording of the groups, facilitator reflection and participant feedback. This will assess facilitator competence, adherence to the course manual and trial procedures and participant interaction and engagement.

Patient and Public Involvement (PPI)

We have had substantial patient and public involvement in the feasibility study prior to finalising this protocol. Lay members were involved in the development of the classification interview, development

For peer review only - http://bmjopen.bmj.com/site/about/guidelines.xhtml

of the intervention, PROM selection, and conduct and management of the study via the independent programme steering and trial management group.

Our trial management group includes our lay co-applicants who are representatives of three leading UK migraine charities (The Migraine Trust, Migraine Action (before their merger with Migraine Trust), and National Migraine Centre).

We have developed a lay steering group who are and will be collaboratively involved during the study. At key points in the programme we will approach the lay steering group for input, this will include at the end of the trial when results are disseminated.

ETHICS AND DISSEMINATION

The University of Warwick (Research Impact Services, University of Warwick, Coventry CV4 7AL) are the Sponsor for the study.

Ethics approval was provided by: North West - Greater Manchester East Research Ethics Committee (REC REF: 16/NW/0890) The trial is conducted in accordance with the principles outlined in the Declaration of Helsinki and conforms to GCP and SOPs set-out by the WCTU. As reported above we have convened a PSC and DMC to oversee the trial.

All identifiable data is anonymised and treated as confidential. Participants are informed that they are free to withdraw at any time during any phase of the work.

The findings from this trial will be disseminated via a final report to the NIHR, presentations at conferences and publications in high quality peer-reviewed journals.

For the healthcare professionals involved in the study we will disseminate results of the study through the study website. We will also host a meeting to present the trial results to commissioners and clinicians. For the participants, we will provide a written lay summary of the findings and also publish these on a study specific website; with contact information should they wish to discuss the findings. Our charity partners will be involved with feedback to the organisations they represent.

CONCLUSION

At the time of writing (08/08/2019) a total of 736 participants have been randomised (380 to the intervention arm and 356 to the control arm) and the trial is now in the follow-up phase.
A comprehensive programme of preparatory work has allowed this substantial trial of an education and self-management intervention for people living with chronic headaches to be developed. There is a clear interest in taking part in the trial. Our follow up will be complete in early 2020 and the final results published in 2021.

For peer review only - http://bmjopen.bmj.com/site/about/guidelines.xhtml

REFERENCES

1. Global, regional, and national burden of neurological disorders during 1990-2015: a systematic analysis for the Global Burden of Disease Study 2015. The Lancet. Neurology 2017;**16**(11):877-97.
2. Henning V, Katsarava Z, Obermann M, Moebus S, Schramm S. Remission of chronic headache: Rates, potential predictors and the role of medication, follow-up results of the German Headache Consortium (GHC) Study. Cephalalgia : an international journal of headache 2018;**38**(3):551-60.
3. Kristoffersen ES, Lundqvist C, Russell MB. Illness perception in people with primary and secondary chronic headache in the general population. Journal of psychosomatic research 2019;**116**:83-92.
4. Westergaard ML, Lau CJ, Allesoe K, Gjendal ST, Jensen RH. Monitoring chronic headache and medication-overuse headache prevalence in Denmark. Cephalalgia : an international journal of headache 2019:333102419876909.
5. Seshia SS, Wober-Bingol C, Guidetti V. The classification of chronic headache: room for further improvement? Cephalalgia : an international journal of headache 2010;**30**(10):1268-70.
6. Steiner TJ, Scher AI, Stewart WF, Kolodner K, Liberman J, Lipton RB. The prevalence and disability burden of adult migraine in England and their relationships to age, gender and ethnicity. Cephalalgia : an international journal of headache 2003;**23**(7):519-27.
7. Stovner L, Hagen K, Jensen R, et al. The global burden of headache: a documentation of headache prevalence and disability worldwide. Cephalalgia : an international journal of headache 2007;**27**(3):193-210.
8. Castillo J, Munoz P, Guitera V, Pascual J. Epidemiology of chronic daily headache in the general population. Headache 1999;**39**(3):190-6.
9. Lu SR, Fuh JL, Chen WT, Juang KD, Wang SJ. Chronic daily headache in Taipei, Taiwan: prevalence, follow-up and outcome predictors. Cephalalgia : an international journal of headache 2001;**21**(10):980-6.
10. Wang SJ, Fuh JL, Lu SR, et al. Chronic daily headache in Chinese elderly: prevalence, risk factors, and biannual follow-up. Neurology 2000;**54**(2):314-9.
11. Mehuys E, Paemeleire K, Van Hees T, et al. Self-medication of regular headache: a community pharmacy-based survey. European journal of neurology 2012;**19**(8):1093-9.
12. Probyn K, Bowers H, Caldwell F, et al. Prognostic factors for chronic headache: A systematic review. Neurology 2017;**89**(3):291-301.
13. Nichols VP, Ellard DR, Griffiths FE, Kamal A, Underwood M, Taylor SJC. The lived experience of chronic headache: a systematic review and synthesis of the qualitative literature. BMJ open 2017;**7**(12):e019929.
14. Probyn K, Bowers H, Mistry D, et al. Non-pharmacological self-management for people living with migraine or tension-type headache: a systematic review including analysis of intervention components. BMJ open 2017;**7**(8):e016670.
15. Carnes D, Homer K, Underwood M, Pincus T, Rahman A, Taylor SJ. Pain management for chronic musculoskeletal conditions: the development of an evidence-based and theory-informed pain self-management course. BMJ open 2013;**3**(11):e003534.
16. Pinnock H, Parke HL, Panagioti M, et al. Systematic meta-review of supported self-management for asthma: a healthcare perspective. BMC medicine 2017;**15**(1):64.
17. Taylor SJC, Pinnock H, Epiphaniou E, et al. Health Services and Delivery Research. A rapid synthesis of the evidence on interventions supporting self-management for people with long-term conditions: PRISMS - Practical systematic Review of Self-Management Support for long-term conditions. Southampton (UK): NIHR Journals Library 2014.
18. Carville S, Padhi S, Reason T, Underwood M. Diagnosis and management of headaches in young people and adults: summary of NICE guidance. BMJ (Clinical research ed.) 2012;**345**:e5765.

For peer review only - http://bmjopen.bmj.com/site/about/guidelines.xhtml

19. Chan AW, Tetzlaff JM, Altman DG, et al. SPIRIT 2013 statement: defining standard protocol items for clinical trials. Annals of internal medicine 2013;**158**(3):200-7.
20. Louter MA, Ward TN, Terwindt GM. Chronic headache: What's in a name? Neurology 2017;**89**(3):224-25.
21. Potter R, Hee SW, Griffiths F, et al. Development and validation of a telephone classification interview for common chronic headache disorders. J Headache Pain 2019;**20**(1):2.
22. Taylor SJC, Carnes D, Homer K, et al. Programme Grants for Applied Research. Improving the self-management of chronic pain: COping with persistent Pain, Effectiveness Research in Self-management (COPERS). Southampton (UK): NIHR Journals Library, 2016.
23. Patel S, Potter R, Matharu M, et al. Development of an education and self-management intervention for chronic headache - CHESS trial (Chronic Headache Education and Self-management Study). J Headache Pain 2019;**20**(1):28.
24. Taylor SJ, Carnes D, Homer K, et al. Novel Three-Day, Community-Based, Nonpharmacological Group Intervention for Chronic Musculoskeletal Pain (COPERS): A Randomised Clinical Trial. PLoS medicine 2016;**13**(6):e1002040.
25. NICE. Headaches in over 12s: diagnosis and management - Clinical guideline [CG150], 2012.
26. Kosinski M, Bayliss MS, Bjorner JB, et al. A six-item short-form survey for measuring headache impact: the HIT-6. Quality of life research : an international journal of quality of life aspects of treatment, care and rehabilitation 2003;**12**(8):963-74.
27. Haywood KL, Mars TS, Potter R, Patel S, Matharu M, Underwood M. Assessing the impact of headaches and the outcomes of treatment: A systematic review of patient-reported outcome measures (PROMs). Cephalalgia : an international journal of headache 2018;**38**(7):1374-86.
28. Jhingran P, Osterhaus JT, Miller DW, Lee JT, Kirchdoerfer L. Development and validation of the Migraine-Specific Quality of Life Questionnaire. Headache 1998;**38**(4):295-302.
29. Martin BC, Pathak DS, Sharfman MI, et al. Validity and reliability of the migraine-specific quality of life questionnaire (MSQ Version 2.1). Headache 2000;**40**(3):204-15.
30. McKenna SP, Doward LC, Davey KM. The Development and Psychometric Properties of the MSQOL: A Migraine-Specific Quality-of-Life Instrument. Clinical drug investigation 1998;**15**(5):413-23.
31. Gandek B, Ware JE, Aaronson NK, et al. Cross-validation of item selection and scoring for the SF-12 Health Survey in nine countries: results from the IQOLA Project. International Quality of Life Assessment. Journal of clinical epidemiology 1998;**51**(11):1171-8.
32. Herdman M, Gudex C, Lloyd A, et al. Development and preliminary testing of the new five-level version of EQ-5D (EQ-5D-5L). Quality of life research : an international journal of quality of life aspects of treatment, care and rehabilitation 2011;**20**(10):1727-36.
33. Jenkinson C, Layte R, Lawrence K. Development and testing of the Medical Outcomes Study 36-Item Short Form Health Survey summary scale scores in the United Kingdom. Results from a large-scale survey and a clinical trial. Medical care 1997;**35**(4):410-6.
34. Zigmond AS, Snaith RP. The hospital anxiety and depression scale. Acta psychiatrica Scandinavica 1983;**67**(6):361-70.
35. Nicholas MK. The pain self-efficacy questionnaire: Taking pain into account. European journal of pain (London, England) 2007;**11**(2):153-63.
36. Miles CL, Pincus T, Carnes D, Taylor SJ, Underwood M. Measuring pain self-efficacy. The Clinical journal of pain 2011;**27**(5):461-70.
37. Osborne RH, Elsworth GR, Whitfield K. The Health Education Impact Questionnaire (heiQ): an outcomes and evaluation measure for patient education and self-management interventions for people with chronic conditions. Patient education and counseling 2007;**66**(2):192-201.
38. Hagen K, Linde M, Steiner TJ, Zwart JA, Stovner LJ. The bidirectional relationship between headache and chronic musculoskeletal complaints: an 11-year follow-up in the Nord-Trondelag Health Study (HUNT). European journal of neurology 2012;**19**(11):1447-54.

For peer review only

39. Jimenez-Sanchez S, Fernandez-de-Las-Penas C, Carrasco-Garrido P, et al. Prevalence of chronic head, neck and low back pain and associated factors in women residing in the Autonomous Region of Madrid (Spain). Gaceta sanitaria 2012;**26**(6):534-40.
40. Plesh O, Adams SH, Gansky SA. Self-reported comorbid pains in severe headaches or migraines in a US national sample. Headache 2012;**52**(6):946-56.
41. Yoon MS, Manack A, Schramm S, et al. Chronic migraine and chronic tension-type headache are associated with concomitant low back pain: results of the German Headache Consortium study. Pain 2013;**154**(3):484-92.
42. Parsons S, Carnes D, Pincus T, et al. Measuring troublesomeness of chronic pain by location. BMC musculoskeletal disorders 2006;**7**:34.
43. Cousins S, Ridsdale L, Goldstein LH, Noble AJ, Moorey S, Seed P. A pilot study of cognitive behavioural therapy and relaxation for migraine headache: a randomised controlled trial. Journal of neurology 2015;**262**(12):2764-72.
44. White K, Potter R, Patel S, et al. Chronic Headache Education and Self-management Study (CHESS) - a mixed method feasibility study to inform the design of a randomised controlled trial. Submitted. 2019;**19**(30).
45. Moerbeek M, Wong WK. Sample size formulae for trials comparing group and individual treatments in a multilevel model. Statistics in medicine 2008;**27**(15):2850-64.
46. Brookes ST, Whitely E, Egger M, Smith GD, Mulheran PA, Peters TJ. Subgroup analyses in randomized trials: risks of subgroup-specific analyses; power and sample size for the interaction test. Journal of clinical epidemiology 2004;**57**(3):229-36.
47. Authority NBS. Prescription Cost Analysis (PCA) data. Secondary Prescription Cost Analysis (PCA) data. https://www.nhsbsa.nhs.uk/prescription-data/dispensing-data/prescription-cost-analysis-pca-data.
48. van Hout B, Janssen MF, Feng YS, et al. Interim scoring for the EQ-5D-5L: mapping the EQ-5D-5L to EQ-5D-3L value sets. Value in health : the journal of the International Society for Pharmacoeconomics and Outcomes Research 2012;**15**(5):708-15.
49. Nichols VP, Ellard DR, Griffiths FE, Underwood M, Taylor SJC, Patel S. The CHESS trial: protocol for the process evaluation of a randomised trial of an education and self-management intervention for people with chronic headache. Trials 2019;**20**(1):323.

Figure legend

Figure 1 - Shows the recruitment flow chart

For peer review only

ABBREVIATIONS

Abbreviation	Full form
CHESS	Chronic Headache Education and Self-management Study
HIT6	Headache Impact Test
MOH	Medication Overuse Headaches
NICE	National Institute for Health and Care Excellence
RCT	Randomised Controlled Trial
GP	General Practitioner
PPI	Patient and Public Involvement
PROMs	Patient-Reported Outcome Measures
MSQ	Migraine-Specific Quality of Life Questionnaire
CHQLQ	Chronic Headache Quality of Life Questionnaire
SF12	Short Form 12-item Health Survey
EQ-5D-5L	EuroQoL
HADs	Hospital Anxiety and Depression Scale
PSEQ	Pain Self-Efficacy Questionnaire
HeiQ	Health Education Impact Questionnaire
ICC	intra-class correlation coefficient
DMEC	Data Monitoring and Ethics Committee
WCTU	Warwick Clinical Trials Unit
App	Application
CACE	Complier Averaged Causal Effect
CI	Confidence Interval
QALYs	Quality-Adjusted Life-Years
PSC	Programme Steering Committee
AE	Adverse Event
DCM	Definite Chronic Migraine
PCM	Probable Chronic Migraine
TTH	Tension Type Headache

DECLARATIONS

Ethics approval and consent to participate

Ethics approval was given on the 17th Of February 2017 by North West - Greater Manchester East Research Ethics Committee (REC REF: 16/NW/0890). Written consent was taken for participation.

Competing interests statements

MU is a director and shareholder of Clinvivo Ltd. Use of this company was specified in original application for funding to NIHR. MU has recused himself from all discussion regarding the use of the App in this study. All contracting processes have been in accord with University of Warwick financial regulations.

MU was Chair of the NICE accreditation advisory committee until March 2017 for which he received a fee. He is chief investigator or co-investigator on multiple previous and current research grants from the UK National Institute for Health Research, Arthritis Research UK and is a co-investigator on grants funded by Arthritis Australia and Australian NHMRC. He has received travel expenses for speaking at conferences from the professional organisations hosting the conferences He is part of an academic partnership with Serco Ltd related to return to work initiatives. He is an editor of the NIHR journal

For peer review only - http://bmjopen.bmj.com/site/about/guidelines.xhtml

series, and a member of the NIHR Journal Editors group for which he receives a fee. He has published multiple papers on chronic pain some of which are referenced in this paper.

MSM serves on the advisory board for Allergan, Medtronic, Novartis and TEVA and has received payment for the development of educational presentations from Allergan, electroCore, Medtronic, Novartis and TEVA.

SP and HS are directors of Health Psychology Services Ltd which in part provides psychological treatments for those with chronic pain.

Funding statement
This research was funded by the NIHR Programme Grants for Applied Research programme (RP-PG-1212-20018). The views expressed in this publication are those of the author(s) and not necessarily those of the NHS, the NIHR or the Department of Health.

Data access
The final trial dataset will be available for sharing with other research teams after publication of the main trial paper. All data will be anonymised and provided after assessment of any further research proposals and evidence of ethical approvals.

Authors contribution
SP - study concept and design, supported writing of the first draft of the manuscript and finalised the manuscript for submission.
DC – study concept and design, supported writing of the first draft of the manuscript, provided critical revisions to the manuscript
SE - study concept and design, provided critical revisions to the manuscript
DE - study concept and design, provided critical revisions to the manuscript
FG - study concept and design, provided critical revisions to the manuscript
KH - study concept and design, provided critical revisions to the manuscript
SWH - study concept and design, provided critical revisions to the manuscript
DM - study concept and design, provided critical revisions to the manuscript
HM - study concept and design, provided critical revisions to the manuscript
VN - study concept and design, provided critical revisions to the manuscript
SPe - study concept and design, provided critical revisions to the manuscript
TP - study concept and design, provided critical revisions to the manuscript
RP - study concept and design, provided critical revisions to the manuscript
HS - study concept and design, provided critical revisions to the manuscript
SJT - study concept and design, provided critical revisions to the manuscript
MU – conceived the original study design and supported writing of the first draft of the manuscript
KW - study concept and design, provided critical revisions to the manuscript
MSM - study concept and design, provided critical revisions to the manuscript

DC, SE, DE, FG, KH, SWH, HM, SPe, TP, HS, SJT, MU, MSM - original grant holders

All authors have read and approved the final manuscript.

Acknowledgements
The manuscript was written on behalf of the CHESS co-applicants, study team and PPI representatives.

For peer review only - http://bmjopen.bmj.com/site/about/guidelines.xhtml

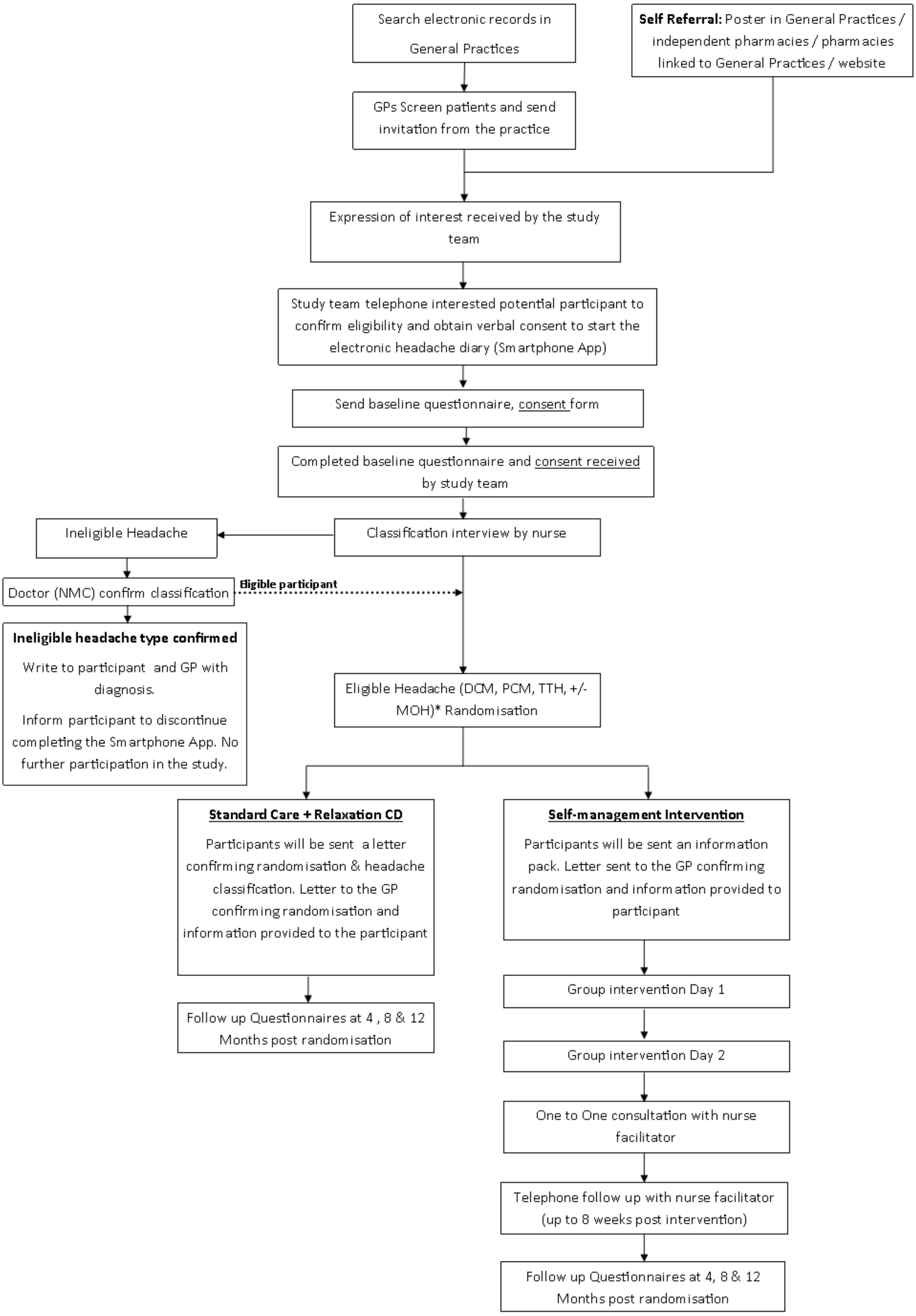

*DMC—Definite Chronic Migraine; PCM— Probable Chronic Migraine; TTH— Tension Type Headache; MOH— Medication Overuse Headache

Reporting checklist for protocol of a clinical trial.

Based on the SPIRIT guidelines.

Instructions to authors

Complete this checklist by entering the page numbers from your manuscript where readers will find each of the items listed below.

Your article may not currently address all the items on the checklist. Please modify your text to include the missing information. If you are certain that an item does not apply, please write "n/a" and provide a short explanation.

Upload your completed checklist as an extra file when you submit to a journal.

In your methods section, say that you used the SPIRITreporting guidelines, and cite them as:

Chan A-W, Tetzlaff JM, Altman DG, Laupacis A, Gøtzsche PC, Krleža-Jerić K, Hróbjartsson A, Mann H, Dickersin K, Berlin J, Doré C, Parulekar W, Summerskill W, Groves T, Schulz K, Sox H, Rockhold FW, Rennie D, Moher D. SPIRIT 2013 Statement: Defining standard protocol items for clinical trials. Ann Intern Med. 2013;158(3):200-207

		Reporting Item	Page Number
**Administrative information**			
Title	#1	Descriptive title identifying the study design, population, interventions, and, if applicable, trial acronym	1

For peer review only

Trial registration	#2a	Trial identifier and registry name. If not yet registered, name of intended registry	3
Trial registration: data set	#2b	All items from the World Health Organization Trial Registration Data Set	N/A
Protocol version	#3	Date and version identifier	3
Funding	#4	Sources and types of financial, material, and other support	22
Roles and responsibilities: contributorship	#5a	Names, affiliations, and roles of protocol contributors	1 & 2
Roles and responsibilities: sponsor contact information	#5b	Name and contact information for the trial sponsor	17
Roles and responsibilities: sponsor and funder	#5c	Role of study sponsor and funders, if any, in study design; collection, management, analysis, and interpretation of data; writing of the report; and the decision to submit the report for publication, including whether they will have ultimate authority over any of these activities	N/A
Roles and responsibilities: committees	#5d	Composition, roles, and responsibilities of the coordinating centre, steering committee, endpoint adjudication committee, data management team, and	15-17

For peer review only

		other individuals or groups overseeing the trial, if applicable (see Item 21a for data monitoring committee)	
**Introduction**			
Background and rationale	#6a	Description of research question and justification for undertaking the trial, including summary of relevant studies (published and unpublished) examining benefits and harms for each intervention	5
Background and rationale: choice of comparators	#6b	Explanation for choice of comparators	5
Objectives	#7	Specific objectives or hypotheses	5
Trial design	#8	Description of trial design including type of trial (eg, parallel group, crossover, factorial, single group), allocation ratio, and framework (eg, superiority, equivalence, non-inferiority, exploratory)	5
**Methods: Participants, interventions, and outcomes**			
Study setting	#9	Description of study settings (eg, community clinic, academic hospital) and list of countries where data will	6

For peer review only

		be collected. Reference to where list of study sites can be obtained	
Eligibility criteria	#10	Inclusion and exclusion criteria for participants. If applicable, eligibility criteria for study centres and individuals who will perform the interventions (eg, surgeons, psychotherapists)	6
Interventions: description	#11a	Interventions for each group with sufficient detail to allow replication, including how and when they will be administered	7
Interventions: modifications	#11b	Criteria for discontinuing or modifying allocated interventions for a given trial participant (eg, drug dose change in response to harms, participant request, or improving / worsening disease)	N/A
Interventions: adherance	#11c	Strategies to improve adherence to intervention protocols, and any procedures for monitoring adherence (eg, drug tablet return; laboratory tests)	8 & 16
Interventions: concomitant care	#11d	Relevant concomitant care and interventions that are permitted or prohibited during the trial	N/A
Outcomes	#12	Primary, secondary, and other outcomes, including the specific measurement variable (eg, systolic blood pressure), analysis metric (eg, change from baseline, final value, time to event), method of aggregation (eg, median, proportion), and time point for each outcome.	8-11

For peer review only

		Explanation of the clinical relevance of chosen efficacy and harm outcomes is strongly recommended	
Participant timeline	#13	Time schedule of enrolment, interventions (including any run-ins and washouts), assessments, and visits for participants. A schematic diagram is highly recommended (see Figure)	10 & FIG 1
Sample size	#14	Estimated number of participants needed to achieve study objectives and how it was determined, including clinical and statistical assumptions supporting any sample size calculations	11-12
Recruitment	#15	Strategies for achieving adequate participant enrolment to reach target sample size	12-13
**Methods: Assignment of interventions (for controlled trials)**			
Allocation: sequence generation	#16a	Method of generating the allocation sequence (eg, computer-generated random numbers), and list of any factors for stratification. To reduce predictability of a random sequence, details of any planned restriction (eg, blocking) should be provided in a separate document that is unavailable to those who enrol participants or assign interventions	13

For peer review only

Allocation concealment mechanism	#16b	Mechanism of implementing the allocation sequence (eg, central telephone; sequentially numbered, opaque, sealed envelopes), describing any steps to conceal the sequence until interventions are assigned	13
Allocation: implementation	#16c	Who will generate the allocation sequence, who will enrol participants, and who will assign participants to interventions	13
Blinding (masking)	#17a	Who will be blinded after assignment to interventions (eg, trial participants, care providers, outcome assessors, data analysts), and how	N/A
Blinding (masking): emergency unblinding	#17b	If blinded, circumstances under which unblinding is permissible, and procedure for revealing a participant's allocated intervention during the trial	N/A
**Methods: Data collection, management, and analysis**			
Data collection plan	#18a	Plans for assessment and collection of outcome, baseline, and other trial data, including any related processes to promote data quality (eg, duplicate measurements, training of assessors) and a description of study instruments (eg, questionnaires, laboratory tests) along with their reliability and validity,	13-14

For peer review only

		if known. Reference to where data collection forms can be found, if not in the protocol	
Data collection plan: retention	#18b	Plans to promote participant retention and complete follow-up, including list of any outcome data to be collected for participants who discontinue or deviate from intervention protocols	13-14
Data management	#19	Plans for data entry, coding, security, and storage, including any related processes to promote data quality (eg, double data entry; range checks for data values). Reference to where details of data management procedures can be found, if not in the protocol	14
Statistics: outcomes	#20a	Statistical methods for analysing primary and secondary outcomes. Reference to where other details of the statistical analysis plan can be found, if not in the protocol	14-15
Statistics: additional analyses	#20b	Methods for any additional analyses (eg, subgroup and adjusted analyses)	14-15
Statistics: analysis population and missing data	#20c	Definition of analysis population relating to protocol non-adherence (eg, as randomised analysis), and any statistical methods to handle missing data (eg, multiple imputation)	14-15

Methods: Monitoring

For peer review only

Data monitoring: formal committee	#21a	Composition of data monitoring committee (DMC); summary of its role and reporting structure; statement of whether it is independent from the sponsor and competing interests; and reference to where further details about its charter can be found, if not in the protocol. Alternatively, an explanation of why a DMC is not needed	15-16
Data monitoring: interim analysis	#21b	Description of any interim analyses and stopping guidelines, including who will have access to these interim results and make the final decision to terminate the trial	15-16
Harms	#22	Plans for collecting, assessing, reporting, and managing solicited and spontaneously reported adverse events and other unintended effects of trial interventions or trial conduct	16
Auditing	#23	Frequency and procedures for auditing trial conduct, if any, and whether the process will be independent from investigators and the sponsor	15-16
**Ethics and dissemination**			
Research ethics approval	#24	Plans for seeking research ethics committee / institutional review board (REC / IRB) approval	16-17

For peer review only

Protocol amendments	#25	Plans for communicating important protocol modifications (eg, changes to eligibility criteria, outcomes, analyses) to relevant parties (eg, investigators, REC / IRBs, trial participants, trial registries, journals, regulators)	16-17
Consent or assent	#26a	Who will obtain informed consent or assent from potential trial participants or authorised surrogates, and how (see Item 32)	12-13
Consent or assent: ancillary studies	#26b	Additional consent provisions for collection and use of participant data and biological specimens in ancillary studies, if applicable	N/A
Confidentiality	#27	How personal information about potential and enrolled participants will be collected, shared, and maintained in order to protect confidentiality before, during, and after the trial	17
Declaration of interests	#28	Financial and other competing interests for principal investigators for the overall trial and each study site	21-22
Data access	#29	Statement of who will have access to the final trial dataset, and disclosure of contractual agreements that limit such access for investigators	22
Ancillary and post trial care	#30	Provisions, if any, for ancillary and post-trial care, and for compensation to those who suffer harm from trial participation	N/A

For peer review only

Dissemination policy: trial results	#31a	Plans for investigators and sponsor to communicate trial results to participants, healthcare professionals, the public, and other relevant groups (eg, via publication, reporting in results databases, or other data sharing arrangements), including any publication restrictions	16-17
Dissemination policy: authorship	#31b	Authorship eligibility guidelines and any intended use of professional writers	22
Dissemination policy: reproducible research	#31c	Plans, if any, for granting public access to the full protocol, participant-level dataset, and statistical code	N/A

Appendices

Informed consent materials	#32	Model consent form and other related documentation given to participants and authorised surrogates	APPENDIX 1
Biological specimens	#33	Plans for collection, laboratory evaluation, and storage of biological specimens for genetic or molecular analysis in the current trial and for future use in ancillary studies, if applicable	N/A

None The SPIRIT checklist is distributed under the terms of the Creative Commons Attribution License CC-BY-ND 3.0. This checklist can be completed online using https://www.goodreports.org/, a tool made by the EQUATOR Network in collaboration with Penelope.ai

For peer review only
